# Dendritic cell proliferation by primary cilium in atopic dermatitis

**DOI:** 10.3389/fmolb.2023.1149828

**Published:** 2023-04-26

**Authors:** Manami Toriyama, Defri Rizaldy, Motoki Nakamura, Yukiko Atsumi, Michinori Toriyama, Fumitaka Fujita, Fumihiro Okada, Akimichi Morita, Hiroshi Itoh, Ken J. Ishii

**Affiliations:** ^1^ Graduate School of Pharmacological Sciences, Osaka University, Osaka, Japan; ^2^ Center for Vaccine and Adjuvant Research (CVAR), National Institutes of Biomedical Innovation, Health and Nutrition, Osaka, Japan; ^3^ Graduate School of Science and Technology, Nara Institute of Science and Technology, Nara, Japan; ^4^ School of Pharmacy, Institut Teknologi Bandung, Bandung, Indonesia; ^5^ Department of Geriatric and Environmental Dermatology, Graduate School of Medical Sciences, Nagoya City University, Nagoya, Japan; ^6^ School of Biological and Environmental Sciences, Kwansei Gakuin University, Hyogo, Japan; ^7^ Mandom Corporation, Osaka, Japan; ^8^ Division of Vaccine Science, The Institute of Medical Science, The University of Tokyo, Tokyo, Japan

**Keywords:** primary cilium, langerhans cell, dendritic cell, keratinocytes, atopic dermatitis, pdgf signaling

## Abstract

**Introduction:** Atopic dermatitis (AD) is a common allergic eczema that affects up to 10% of adults in developed countries. Immune cells in the epidermis, namely, Langerhans cells (LCs), contribute to the pathogenesis of AD, although their exact role(s) in disease remain unclear.

**Methods:** We performed immunostaining on human skin and peripheral blood mononuclear cells (PBMCs) and visualized primary cilium.

**Result and discussion:** We show that human dendritic cells (DCs) and LCs have a previously unknown primary cilium-like structure. The primary cilium was assembled during DC proliferation in response to the Th2 cytokine GM-CSF, and its formation was halted by DC maturation agents. This suggests that the role of primary cilium is to transduce proliferation signaling. The platelet-derived growth factor receptor alpha (PDGFRα) pathway, which is known for transducing proliferation signals in the primary cilium, promoted DC proliferation in a manner dependent on the intraflagellar transport (IFT) system. We also examined the epidermal samples from AD patients, and observed aberrantly ciliated LCs and keratinocytes in immature and proliferating states. Our results identify a potential relationship between the primary cilium and allergic skin barrier disorders, and suggest that targeting the primary cilium may contribute to treating AD.

## Introduction

The skin, the largest human tissue, plays immunologically important roles in homeostasis to protect the body from pathogens. Human skin is composed of three layers including the epidermis, the dermis, and the subcutaneous tissue. Keratinocytes (KCs) are the major cells in the epidermis, and their proliferation and differentiation are strictly regulated to maintain skin homeostasis (Barrientos et al., 2008). The outer layer of the epidermis is composed of layers of dead KCs, called the stratum corneum, which forms a brick-like physical barrier that protects the body from pathogens, toxins and other harmful substances in the environment (Madison, 2003). Immune cells such as the Langerhans cells (LCs), which have a similar role as the dendritic cells (DCs), also exist in the epidermis and maintain skin homeostasis by presenting antigens to activate T cells (Nestle et al., 2009; Pasparakis et al., 2014; Deckers et al., 2018). It is reported that LCs are derived from fetal liver, yolk sac and bone morrow, while DCs in the blood are derived from bone marrow in mice, and both share similar functions (Haase and Nestle, 2014; Ginhoux et al., 2006; Hoeffel et al., 2012). When LCs incorporate antigens, they immediately migrate toward the lymph nodes, then activate T cells (Nestle et al., 2009; Pasparakis et al., 2014; Deckers et al., 2018). It has long been accepted that LCs have a pivotal role in bridging innate and acquired immunity, which is necessary for skin homeostasis. Although accumulated evidence shows that LCs are necessary for homeostasis, LCs are also involved in the pathology of allergic skin diseases, such as atopic dermatitis (AD) (Callard and Harper, 2007; Dubrac et al., 2010; Nakajima et al., 2012).

The prevalence of AD has increased greatly in the past 30 years, and it is widely known that environmental factors such as mite antigens in house dust can trigger allergic disease. In both the early and chronic stages of AD, type 2 immune responses, which are characterized by the elevation of several type 2 cytokines and IgE production, are dominant (Gandhi et al., 2016). Chemokines secreted by KCs are upregulated in AD, recruiting Th2 cells which strongly induce Th2 responses (Nedoszytko et al., 2014). AD often features skin barrier disruption caused by abnormal proliferation of KCs, leading to dryness, itchiness, and invasion by pathogens such as *Staphylococcus aureus*. Topical steroids, tacrolimus, and moisturizers are employed in AD treatment, however due to its complicated pathogenesis, AD often recurs after initial improvement. In an important finding, Chorro *et al.* reported that proliferating LCs were significantly increased in AD patients (Chorro et al., 2009) and suggested that these LCs promoted Th2-dominant conditions (Elentner et al., 2009). As such, the regulation of LC/KC proliferation might be effective in AD treatment, but the mechanisms of LC/KC proliferation in AD are not well understood.

The primary cilium is a unique organelle which protrudes into the extracellular space from the cell surface and functions as a platform for signaling pathways (Goetz and Anderson, 2010). The intraflagellar transport (IFT) system is essential for axoneme elongation and ciliary protein transport (Pedersen and Rosenbaum, 2008). *IFT* gene mutations or disruption of this transport system disassemble the primary cilium, resulting in developmental defects, signaling defects and ciliopathy (Fliegauf et al., 2007; Nigg and Raff, 2009; Valente et al., 2014). The primary cilium is formed when cells are in the G0 or G1 phase of the cell cycle. In contrast, ciliary components regulate cell cycle progression and *vice versa* (Goto et al., 2013). Thus, the primary cilium formation and the cell cycle progress tightly regulate each other. It is widely thought that signaling pathways through the primary cilium regulate cell proliferation and differentiation in many types of cells and tissues.

Almost all types of cells, including stromal cells and immune cells, can assemble primary cilium. Ezratty et al. reported that mouse embryonic epidermal KCs have primary cilium, which regulates KC differentiation (Ezratty et al., 2011). In 2015, Prosser and Morrison reported that immortalized T and B cells have primary cilium (Prosser and Morrison, 2015). However, the precise roles of primary cilium in both adult skin and the immune system have not been explored until recently. Here, we found primary cilium in epidermal LCs derived from healthy adult donors and investigated its function. An *in vitro* culture system of primary blood monocytes, DCs and THP1 cell lines suggested the role of primary cilium in regulating DC proliferation. We further analyzed human skin samples of healthy donors and AD patients, and found that primary cilium assembly significantly increased in AD patient samples. Our data suggest that the primary cilium functions in DC proliferation and has physiological relevance in skin homeostasis.

## Results

### Human primary immune cells can assemble a primary cilium-like structure

**FIGURE 1:**
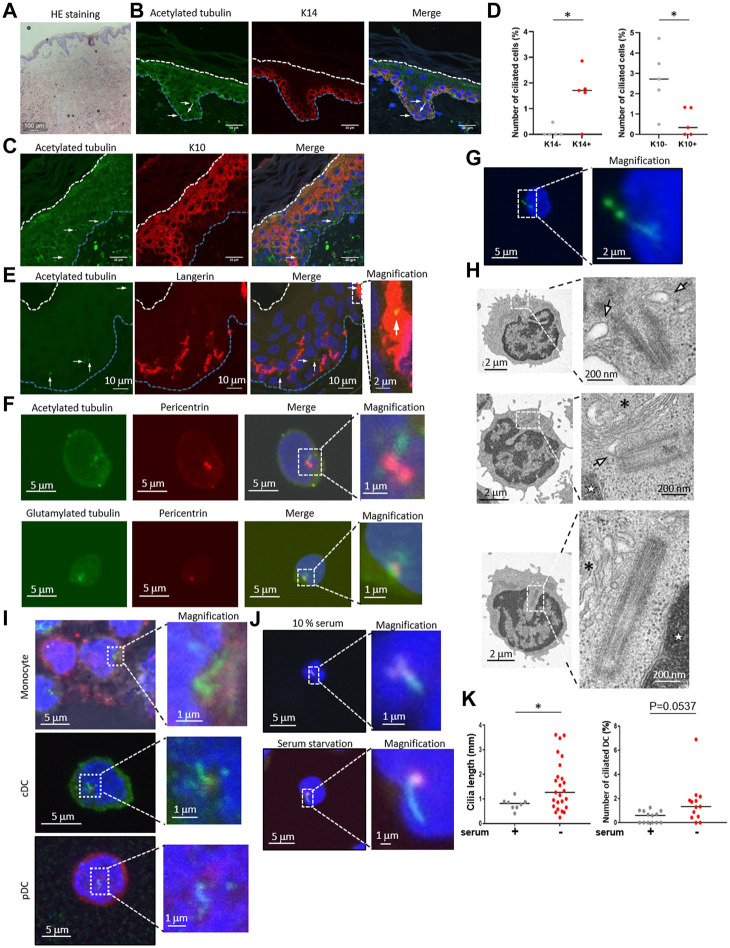
**(A)** Hematoxylin and eosin (HE) staining of healthy human skin. Scale bar: 100 μm. **(B,C)** Representative images of epidermal primary cilium-like structures (acetylated tubulin; green) in **(B)** the K14-positive basal layer (red), and in **(C)** the K10-positive stratum spinosum/granular layer (red). Nuclei were stained with hoechst 33342 (blue). The arrow indicates the primary cilium. The blue dotted line indicates the epidermal basement membrane. The white dotted line indicates the stratum orneum. The area within the dotted box was magnified and shown on the right. Five biological replicates (five individual donors) were examined. Scale bars: 20 μm and 2 μm, respectively. **(D)** The number of primary ciliated cells in the K14-positive basal layer and the K10-positive stratum spinosum/granular layer shown in **(C,D)**. *, p < 0.05 (Mann-Whitney U test). n = 5. K14-; 936 cells were observed and 2 cilia were identified. K14+; 1,248 cells were observed and 19 cilia were identified. K10-; 860 cells were observed and 22 cilia were identified. K10+; 1,203 cells were observed and 7 cilia were identified. **(E)** Representative immunostaining image of langerin-positive LCs (red) and primary cilium (acetylated tubulin; green) in healthy human epidermis. Nuclei were stained with hoechst 33342 (blue). The arrow indicates a primary cilium-like structure. The blue dotted line indicates the epidermal basement membrane. The white dotted line indicates the stratum corneum. The area within the dotted box was magnified and shown on the right. Scale bars: 10 μm and 2 μm, respectively. Five biological replicates (five individual donors) were examined, respectively. **(F)** Representative immunostaining image of acetylated tubulin or polyglutamylated tubulin (green) with centrosome (pericentrin; red) and nuclei (hoechst 33342; blue) in human PBMCs. The dotted box indicates the magnified area shown on the far right. Scale bars: 5 μm and 1 μm, respectively. Three biological replicates (3 donors) were examined. **(G)** Representative immunostaining image of human PBMCs expressing Arl13B-GFP (green). Nuclei were stained with hoechst 33342 (blue). Cells were electroporated with an Arl13B-GFP expression plasmid. Two days after transfection, cells were immunostained by using an anti-GFP antibody. The area within the dotted box was magnified and shown on the bottom. Scale bars: 5 μm and 2 μm, respectively. Three biological replicates (3 donors) were examined. **(H)** Electron microscope images of primary ciliumlike structures in PBMCs. The top two pictures show representative ciliary vesicle-like structures. The bottom picture shows centrosome elongation resembling axoneme extension. For electron microscope analysis, five biological replicates (five donors) were examined. The arrow indicates a ciliary vesicle-like structure. The asterisk indicates the Golgi apparatus. The star indicates the nucleus. The area within the dotted box was magnified and shown on the right. Scale bars: 2 μm and 200 nm, respectively. **(I)** Monocytes, pDCs and cDCs were isolated from human PBMCs using flow cytometry, then cells were immunostained with acetylated tubulin (green), and pericentrin (red). Nuclei were stained with hoechst 33342 (blue). Three biological replicates (3 donors) were examined. The area within the dotted box was magnified and shown on the right. Scale bars: 5 μm and 1 μm, respectively. **(J)** Human cDCs isolated from PBMCs with magnetic beads were cultured in media supplemented with 10% FBS or 0.5% FBS (serum starvation) for 16 h. Cells were immunostained with acetylated tubulin (green) and pericentrin (red). Serum+; 1,772 cells were observed and 8 cilia were identified. Serum-; 1829 cells were observed and 25 cilia were identified. **(K)** The number of primary ciliated cDCs and the cilium length shown in **(J)** were measured. Thirteen biological replicates (13 donors) were examined respectively. The bar indicates the median value. *, p < 0.05 (Mann-Whitney U test).

To examine the primary cilium distribution in human skin, we obtained healthy adult human skin samples (Figure 1A) and visualized the primary cilium in the epidermis and the dermis by staining with a primary cilium marker, acetylated tubulin, using fluorescent confocal microscopy. We hypothesized that dermal fibroblasts have primary cilium, because it was reported that fibroblasts in the human adult heart are ciliated (Villalobos et al., 2019). As expected, we found ciliated vimentin-positive cells in the dermis, suggesting that most ciliated cells were dermal fibroblasts (Supplementary Figure S1A). We also found primary cilium-like structures in the K14-positive epidermal basal area where proliferating KCs are populous (Figure 1B). Primary cilium-like structures were also detected in the K10-positive stratum spinosum and the granular layer where differentiated and matured KCs are present, but at a lower frequency than in the basal layer (Figures 1C, D). We hypothesized that most of the ciliated epidermal cells were KCs because KCs are the major cell type in the epidermis, and many of the ciliated cells were positively stained with K14, a specific marker for KCs (Figures 1B–D).

Although we speculated that most epidermal ciliated cells were KCs, we investigated the possibility of immune cells having primary cilium, because immune cells such as LCs are also frequently present in the epidermis. We immunostained the epidermis with langerin, a LC marker, and with acetylated tubulin. When observing the primary cilium in epidermal LCs, we unexpectedly found that some LCs in the epidermis were positively merged with acetylated tubulin, indicating primary cilium (Figure 1E). We also investigated acetylated tubulin in langerin-positive cells in the dermis, however, we could not find any primary cilium-like structures in these cells. We found that the entire cytosol of dermal langerin-positive cells was strongly stained with an acetylated tubulin, even though the primary cilium was detected in langerin-negative dermal cells (Supplementary Figure S2). We next investigated CD4^+^ and CD8^+^ T cell distribution in skin derived from five healthy donors, and we found these cells only in the dermis, not in the epidermis (Supplementary Figure S3). As with dermal LCs, we did not detect primary cilium-like structures in dermal CD4^+^ and CD8^+^ T cells (Supplementary Figure S3). These results suggest that epidermal LCs can assemble primary cilium.

Primary cilium function in immune cells has not been investigated, although some immune cells can assemble primary cilium in vitro (Prosser and Morrison, 2015). As our data suggested that primary LCs could assemble primary cilium in the healthy human epidermis, we next investigated if human dendritic cells in blood are ciliated. LCs are regarded as being similar to DCs in the epidermis and have a similar function as conventional DCs (cDCs). To investigate whether immune cells in the blood, especially cDCs, are ciliated, we isolated peripheral blood mononuclear cells (PBMCs), a mixture of immune cells, from human peripheral blood, then immunostained them with acetylated or polyglutamylated tubulin to visualize the primary cilium. We found that nearly 2% of PBMC cells had primary cilium-like structures, showing a single protrusion stained with stabilized tubulin that extended from the centrosome marker, pericentrin, of each cell (Figure 1F).

To investigate whether primary cilium markers other than stabilized tubulin could be detected in PBMCs, we overexpressed Arl13B-GFP and detected it with an anti-GFP antibody. Arl13B is known as a specific marker for primary cilium and is required for cilium formation and maintenance (Larkins et al., 2011). We observed a GFP signal localized to a single linear structure resembling primary cilium (Figure 1G), similar to the results with stabilized tubulin (Figure 1F). Please note that endogenous Arl13B was not detected as a single linear structure in PBMCs and LCs even though acetylated tubulin was detected as like primary cilium-like structure in PBMC (data not shown). To further investigate primary cilium-like structures in PBMCs, we used transmission electron microscopy (TEM). We observed a vesicle–centrosome interaction which resembles a ciliary vesicle (Figure 1H). Also, centrosome elongation resembling axoneme extension, which is found in early primary cilium elongation, was observed (Figure 1H). Although no structures resembling a ciliary sheath have been observed so far, our immunostaining and TEM results suggested that human PBMCs can assemble primary cilium.

**FIGURE 2:**
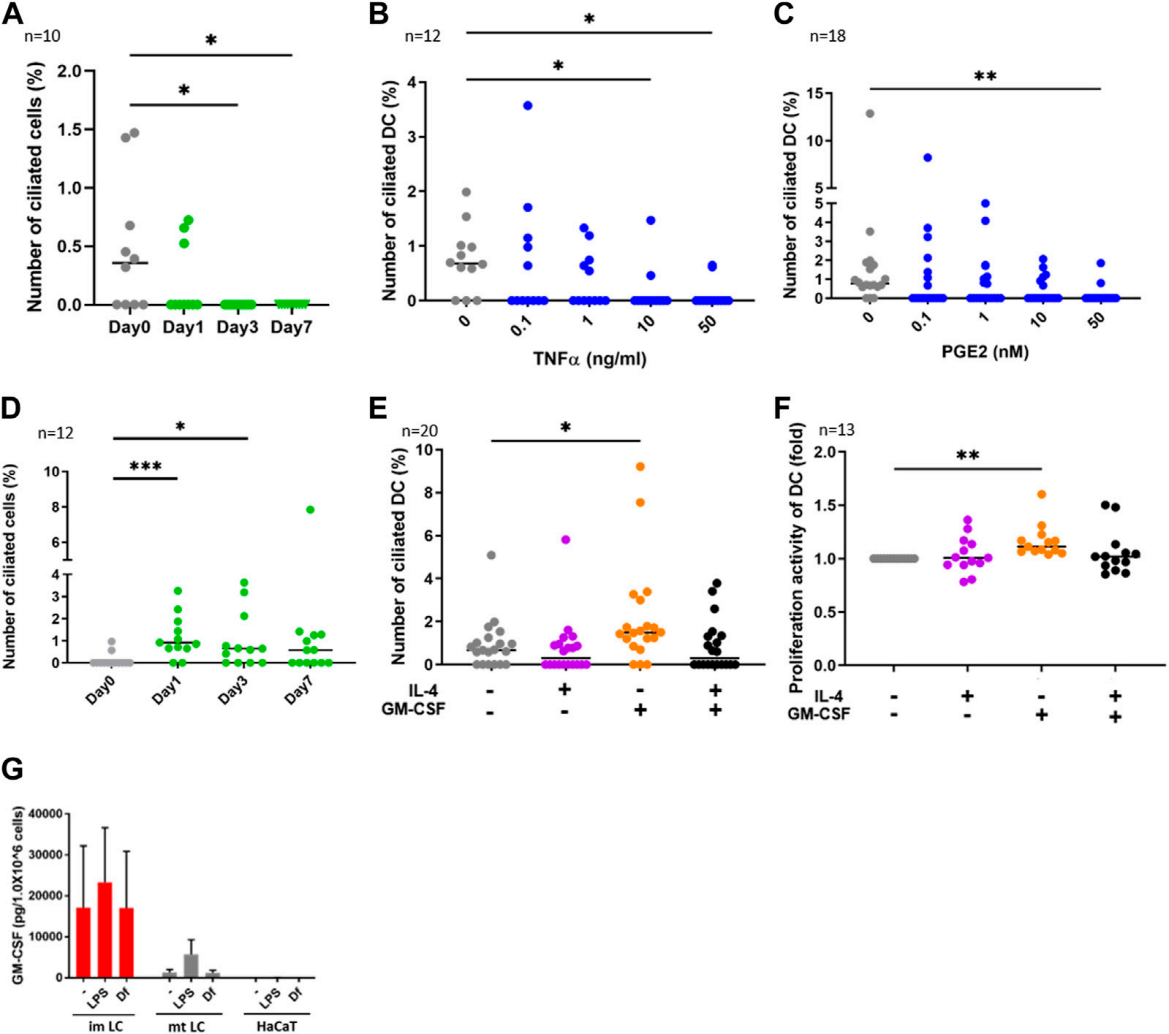
**(A)** CD14^+^ monocytes were isolated from human PBMCs with magnetic beads, then cells were differentiated into DCs by stimulating with 50 ng/ml GM-CSF, 50 ng/mL IL-4 and 50 ng/ mL TNFα.The percentages of primary ciliated cells are shown in the graph. The bar indicates the median value. *, *p* < 0.05 (Kruskal-Wallis and Dunn’s multiple comparison). n = 10 biological replicates (10 donors). Day 0; 1950 cells were observed and 5 cilia were identified. The number of observed cells and primary cilium are as follows: Day 0; 5 cilia/1,950 cells. Day 1; 3 cilia/1,620 cells. Day 3; 0 cilium/2919 cells. Day 7; 0 cilium/989 cells. **(B,C)** Human cDCs isolated from PBMCs were stimulated with **(B)** TNFα or **(C)** PGE2 for 24 h. The percentages of ciliated cDCs are shown in the graph. The bar indicates the median value. *, *p* < 0.05, **, *p* < 0.01 (Kruskal-Wallis and Dunn’s multiple comparison). n = 12 biological replicates (12 donors) and n = 18 biological replicates (18 donors), respectively. The number of observed cells and primary cilium are as follows: 0 ng/mL TNFα; 14 cilia/1,518 cells. 0.1 ng/mL TNFα; 8 cilia/ 1,519 cells. 1 ng/mL TNFα; 5 cilia/1,407 cells. 10 ng/mL TNFα; 2 cilia/1,570 cells. 50 ng/mL TNFα; 2 cilia/1,487 cells. 0 nM PGE2; 15 cilia/1,605 cells. 0.1 nM PGE2; 6 cilia/1,553 cells. 1 nM PGE2; 6 cilia/1,628 cells. 10 nM PGE2; 5 cilia/1,524 cells. 50 nM PGE2; 2 cilia/1,551 cells. **(D)** CD14^+^ monocytes were isolated from human PBMCs with magnetic beads, then cells were differentiated into DCs by stimulating with 50 ng/ml GM-CSF and 50 ng/mL IL-4. The percentages of primary ciliated cells are shown in the graph. The bar indicates the median value. *, *p* < 0.05, ***, *p* < 0.001 (Kruskal-Wallis and Dunn’s multiple comparison). n = 12 biological replicates (12 donors). The number of observed cells and primary cilium are as follows: Day 0; 3 cilia/1,302 cells. Day 1; 15 cilia/1,541 cells. Day 3; 12 cilia/1,724 cells. Day 7; 11 cilia/1,596 cells. **(E)** cDCs isolated from PBMCs were stimulated with 50 ng/mL IL-4 and 50 ng/ml GM-CSF for 24 h. The percentages of primary ciliated cells were counted and graphed. The bar indicates the median value. *, *p* < 0.05, (Kruskal-Wallis and Dunn’s multiple comparison). n = 20 biological replicates (20 donors). **(F)** Five thousand cDCs isolated from PBMCs were cultured with 50 ng/mL IL-4, 50 ng/ml GM-CSF in RPMI 1640 containing 0.5% FBS and 10% CCK8/WST-1 buffer for 48 h. The cell number was calculated by measuring the absorbance. To generate the calibration curve, the absorbance values of 3,000, 5,000, and 10,000 cells were measured. Cell numbers in each sample were calculated from a standard curve, and relative cell numbers were calculated by dividing by the untreated sample value. The bar indicates the median value. **, *p* < 0.01 (Kruskal-Wallis and Dunn’s multiple comparison). n = 13 biological replicates (13 donors). **(G)** Average GM-CSF expression measured by ELISA. Immature LCs (imLCs), mature LCs (mtLCs) and HaCaT cells were stimulated with 10 μg/mL LPS or 10 μg/mL Df for 24 h in media containing 0.5% FBS. Mixtures of culture supernatant and cell lysate were used for the assay. The error bar shows the SEM. n = 5 biological replicates (5 donors). Kruskal-Wallis and Dunn’s multiple comparison were performed.

To identify specific types of ciliated immune cells in PBMC, we isolated monocytes, cDCs, plasmacytoid dendritic cells (pDCs), CD4^+^ T cells, CD8^+^ T cells, natural killer cells, and B cells from human PBMCs by flow cytometry, then immunostained them with acetylated tubulin (Figure 1I, Supplementary Figure S4, Supplementary Figure S5). Except for the CD8^+^ T cells, all of the cells had primary cilium-like structures (Figure 1I, Supplementary Figure S5). Having observed the presence of primary cilium-like structures in human epidermal LCs, we further investigated primary cilium formation in cDCs *in vitro*. Comparison of the frequency of ciliated cDCs isolated from PBMCs and from healthy epidermal LCs was as follows: median rate of ciliated LCs: 0.5235%; median rate of ciliated cDCs isolated by magnetic separation: 0.6961%; median of cDCs isolated by cell sorter: 1.204% (Figures 2B, C, E; Figure 4D).

Since primary cilium formation is strongly associated with cell cycle progress, and serum starvation promotes primary cilium formation in many types of cells by inducing cell cycle arrest (Tang et al., 2013; Orhon et al., 2015), we examined if human primary cDCs use a similar mechanism to elongate primary cilium. Serum starvation by culturing in 0.5% serum for 16 h significantly elongated primary cilium compared to culturing in 10% serum (Figures 1J, K). The frequency of ciliated cells tended to be increased compared to cells treated in 10% serum, but the difference was not statistically significant (Figures 1J, K). This result suggests that human primary cDCs have similar machinery as other cells to elongate primary cilium. In summary, our results suggest the presence of primary cilium in human cDCs and human epidermal LCs.

### cDC maturation decreases primary cilium formation

We next investigated the function of primary cilium in cDCs. DCs can be differentiated from monocytes *in vitro* (Nair et al., 2012). To analyze whether monocyte-derived DCs are ciliated *in vitro*, we isolated CD14^+^ monocytes from human PBMCs and cultured them for 7 days by stimulating with GM-CSF, IL-4 and TNFα to induce differentiation into mature DCs. We found that the frequency of primary cilium in monocytes significantly decreased with time (Figure 2A). Cell viability at day 7 was 80%, suggesting that the decrease in primary cilium was not caused by cell death (data not shown). To ask if DC maturation decreases primary cilium formation, we stimulated primary cDCs with the maturation inducer TNFα (Figure 2B) or prostaglandin E2 (PGE2, Figure 2C) for 24 h. We observed that a high concentration of either TNFα and PGE2 significantly reduced the percentage of primary ciliated cells (Figures 2B,C). These data suggest that maturation of cDCs decreased primary cilium formation. We next induced immature DCs by culturing CD14^+^ monocytes with GM-CSF and IL-4, without TNFα. In contrast to monocyte-derived mature DCs, nearly 1% (median) of monocyte-derived immature DCs at day 7 were ciliated, similar to the proportion in primary cDCs (Figures 2B–D). The percentage of primary ciliated cells did not differ significantly from day 1 to day 7 (Figure 2D, statistics not shown). Cell viability at day 7 was 82.4%, and there were no significant differences between TNFα treated monocytes (80%) at day 7 (data not shown). These results raised the possibility again that primary cilium formation was inhibited while monocytes differentiated into mature DCs.

As we found that the frequency of primary cilium in cytokine-treated monocytes (monocyte-derived immature DCs) at day 1 and day 3 was higher than that in untreated (day 0) monocytes (Figure 2D), we next asked if IL-4 or GM-CSF promoted primary cilium formation in DCs. We found that GM-CSF treatment for 24 h significantly promoted primary cilium formation in primary cDCs (Figure 2E). However, IL-4 itself, or co-stimulation of IL-4 with GM-CSF, did not affect primary cilium formation. It is widely known that immature or precursor cells proliferate more strongly than mature cells (Ruijtenberg and van den Heuvel, 2016), so we next investigated the effect of cytokines on proliferation activity in cDCs. We cultured primary cDCs with GM-CSF or IL-4 for 24 h and analyzed cell growth by a CCK8/WST-1 assay. We found that GM-CSF increased the proliferation activity of primary cDCs, but IL-4, or co-treatment of GM-CSF with IL-4, did not (Figure 2F). These findings raised the possibility that primary cilium formation was correlated with DC proliferation, and GM-CSF is a candidate to promote primary cilium formation in cDCs.

GM-CSF is a Th2 cytokine that promotes DC proliferation, and its expression is elevated in KCs in AD (Pastore et al., 1997). House dust mite antigen derived from *Dermatophagoides farinae* (*Df*) is a risk factor to trigger AD (Bumbacea et al., 2020). We next asked if epidermal cells such as KCs and LCs secrete GM-CSF in response to extracellular stimuli. We stimulated epidermal cells with mite antigen *Df*, or lipopolysaccharide (LPS), which activates Toll-like receptor 4 (TLR4) and induces strong Th1 responses in cDCs. Since primary epidermal cells, including LCs and KCs, often change their morphology and maturation marker expression after their isolation from tissues, we induced immature and mature LCs by stimulating CD14^+^ monocytes with cytokines, then measured GM-CSF expression in these and in human immortalized KCs (HaCaT). We found that monocyte-derived immature LCs expressed GM-CSF more strongly than mature LCs and HaCaT, however the difference was not statistically significant (Figure 2G). As we found that GM-CSF was expressed without any stimulation in monocyte-derived immature LCs, we suggest that immature LCs spontaneously express GM-CSF (Figure 2G). In contrast, IL-4 was not detected in monocyte-derived immature and mature LCs, while HaCaT expressed IL-4 spontaneously (Supplementary Figure S6). These results suggest that immature LCs in the epidermis are the main producers of GM-CSF.

### *Dermatophagoides farinae* (*Df*) antigen tends to promote primary cilium formation in DCs

**FIGURE 3:**
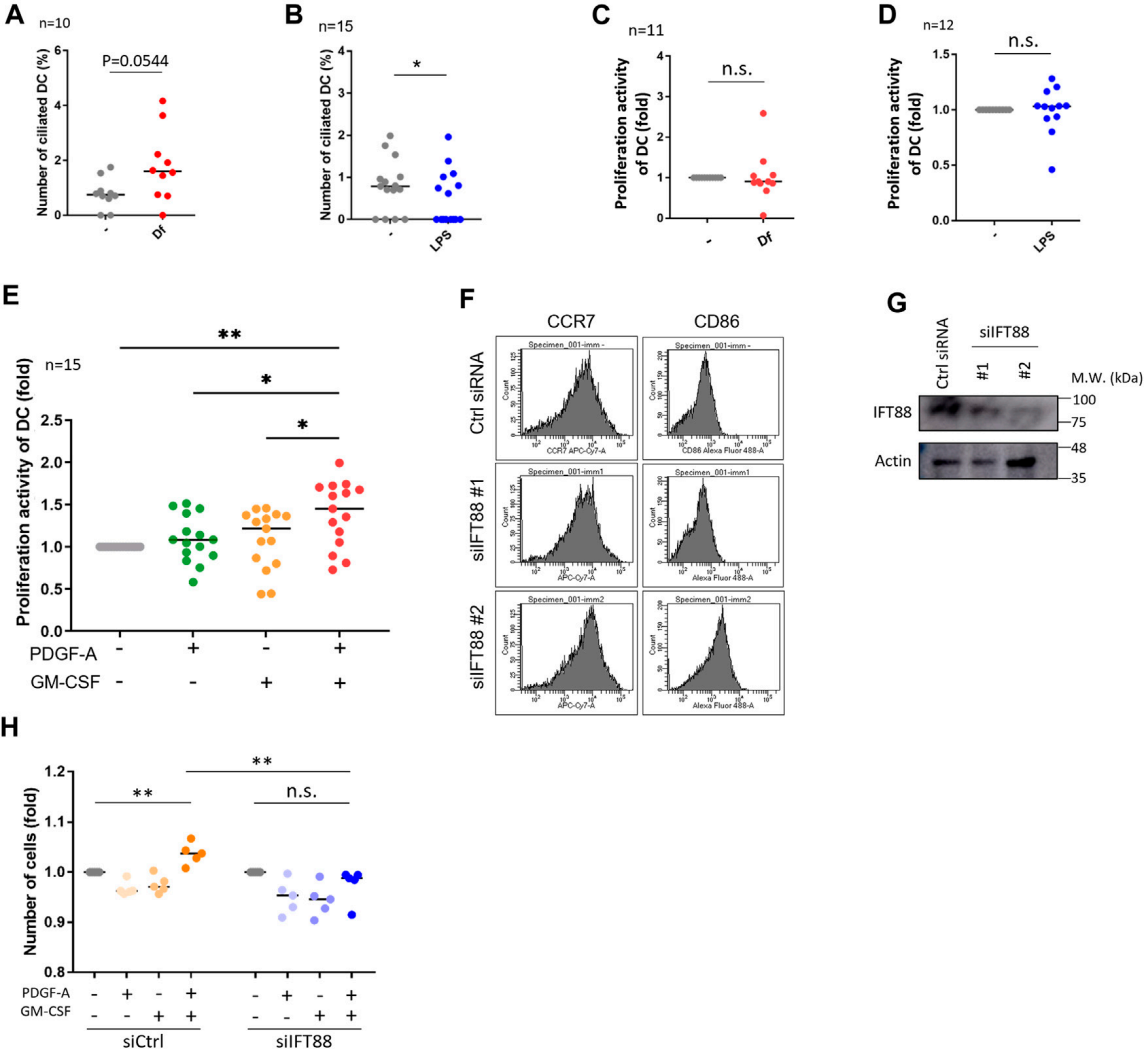
IFT88 downregulation increases maturation marker expression with attenuating proliferation activity. **(A)** cDCs were stimulated with 1 μg/mL Df for 48 h. After immunostaining of acetylated tubulin, the percentage of primary ciliated cells was determined. n = 10 biological replicates (10 donors). The number of observed cells and primary cilium are as follows: no treatment; 8 cilia/1,074 cells. Df; 17 cilia/1,055 cells. The bar indicates the median value. **, *p* < 0.01 (Mann-Whitney U test). **(B)** cDCs were stimulated with 1 <g/mL LPS for 24 h. After immunostaining of acetylated tubulin, primary ciliated cDCs were counted and their numbers graphed. n = 15 biological replicates (15 donors). The number of observed cells and primary cilium are as follows: no treatment; 13 cilia/1,679 cells. LPS; 7 cilia/1687 cells. The bar indicates the median rate. *, *p* < 0.05, Mann-Whitney U test. **(C)** Five thousand cDCs were stimulated with 1 μg/mL Df for 48 h, then the cell number was calculated by the CCK8/WST-1 assay. To generate the calibration curve, the absorbance values of 3,000, 5,000, and 10,000 cells were measured. Cell numbers in each sample were calculated from the standard curve. Relative cell numbers were calculated by dividing by the untreated sample value. The bar indicates the median value. The Mann-Whitney U test was performed. n = 11 biological replicates (11 donors). **(D)** Five thousand cDCs were stimulated with 1 μg/mL LPS for 48 h, then relative cell numbers were calculated by performing the CCK8/WST-1 assay. The bar indicates the median value. The Mann Whitney U test was performed. n = 12 biological replicates (12 donors). **(E)** Five thousand cDCs were stimulated with 10 ng/mL PDGF-A or 10 ng/ml GM-CSF for 48 h. Relative numbers of cDCs were determined by the CCK8/WST-1 assay. For the negative control of PDGF-A stimulation, PDGF-A solvent (4 mM HCl, 0.1% BSA) was added. The bar indicates the median value. Kruskal-Wallis and Dunn’s multiple comparison was used for statistical analysis. *, *p* < 0.05, **, *p* < 0.01, n = 15 biological replicates (15 donors). **(F)** Representative expression of maturation markers in DCs. To differentiate immature DCs, THP1 cells were cultured with 100 ng/ml GM-CSF and 100 ng/mL IL-4 for 5 days. On day 5, cells were electroporated with 20 nM siRNA targeting IFT88 or control siRNA. Two days after electroporation, the expression of cell markers was analyzed by flow cytometry. n = 3 biological replicates. **(G)** Representative image of IFT88 in THP1-derived immature DC detected by Western blotting. n = 3 biological replicates (3 donors). **(H)** Immature DCs derived from THP1 were electroporated with 20 nM siRNA. Two days after electroporation, 5,000 cells were stimulated with 10 ng/mL PDGF-A or 10 ng/ml GM-CSF for 48 h. A CCK8/WST-1 assay was performed to calculate the cell number. The bar indicates the median value. **, *p* < 0.01 (one-way ANOVA, Tukey’s multiple comparison). n = 5 biological replicates.

We then investigated if mite antigen *Df* or LPS regulate primary cilium formation in cDCs. We stimulated primary cDCs with LPS and house dust mite antigen *Df*. *Df* antigen slightly, but not significantly, increased the population of ciliated cDCs (Figure 3A). In contrast, LPS significantly decreased the population of primary ciliated cDCs (Figure 3B). We next analyzed if these immunostimulants affect the proliferation activity of cDCs. Contrary to expectations, LPS and *Df* treatment did not affect proliferation activity in cDCs (Figures 3C, D).

HaCaT showed different responses to immunostimulants compared to cDCs. LPS stimulation significantly decreased HaCaT proliferation, while *Df* increased it (Supplementary Figure S7A). The proliferation marker Ki67 increased after stimulation with Df in HaCaT (Supplementary Figures S7B, C). Importantly, we could not observe any primary cilium in primary KCs or in HaCaT culture *in vitro* despite stimulation with several reagents including *Df*, LPS, GM-CSF and IL-4 (data not shown). From these data, we hypothesized that *Df* is one of the candidates regulating primary ciliation in cDCs and KC proliferation.

### Downregulation of the primary cilium component *IFT88* decreases proliferation activity promoted by PDGFRα signaling in THP1-derived DCs

Platelet-derived growth factor receptor A (PDGFRα) is highly and specifically localized in the primary cilium (Schneider et al., 2005). Platelet-derived growth factor A (PDGF-A), a specific ligand for PDGFRα, promotes fibroblast and KC proliferation during wound healing of the skin (Barrientos et al., 2008). We detected PDGFRα expression in primary cDCs and HaCaT using Western blotting (Supplementary Figures S7B, S8B, D). To identify the types of cells secreting PDGF-A, we performed an ELISA assay by using a mixture of culture supernatant and cell lysate from monocyte-derived immature and mature LCs, and from HaCaT. HaCaT spontaneously expressed PDGF-A, whereas monocyte-derived LCs did not. This result suggests that KCs can secrete PDGF-A (Supplementary Figure S8A).

As we detected the expression of PDGFRα in primary cDCs and HaCaT, we then analyzed the effect of PDGFRα signaling on proliferation in these cells. Co-treatment of primary cDCs and HaCaT with PDGF-A and GM-CSF significantly increased their proliferation activity, while stimulation with the individual factors GM-CSF or PDGF-A did not affect proliferation (Figure 3E, Supplementary Figure S8C). Cilium formation in cells were increased in GM-CSF treated group (Supplementary Figures S8D, E). Furthermore, a trend towards an increase in primary cilia formation rate was observed with GM-CSF = PDGF-A compared to unstimulated cells (Supplementary Figures S8D, E). HaCaT stimulated with GMCSF or PDGF-A individually did not change Ki67 expression, but co-treatment of GM-CSF and PDGF-A tended to increase it although no significant differences were observed (Supplementary Figures S8F, G).

PDGFRα is upregulated during primary cilium formation, and upregulation and activation of PDGFRα signaling are blocked in *Tg737* ^orpk^ (*IFT88*/*Polaris*) mutant MEF cells (Schneider et al., 2005). Therefore, we investigated if primary cilium disruption by *IFT88* knockdown causes proliferation defects. We performed a knockdown experiment by inducing siRNA targeting *IFT88*. We first tried to downregulate *IFT88* in primary cDCs, however, knockdown experiments using electroporation or other reagents were unsuccessful, so we used THP1-derived immature or mature DCs. THP1 cells were differentiated into immature DCs by stimulating them with 100 ng/ml GM-CSF and 100 ng/mL IL-4, or were differentiated into mature DCs by stimulating them with 200 ng/ml GM-CSF, 100 ng/mL IL-4, 20 ng/mL TNFα, and 200 ng/mL ionomycin for 5 days. At day 3, cells were electroporated with siRNA and cultured for 2 more days, then maturation markers were analyzed by using flow cytometry (Figure 3F; Supplementary Figure S9). The knockdown of IFT88 increased DC maturation markers, cluster of differentiation 86 (CD86) and C-C chemokine receptor type 7 (CCR7) in THP1-derived immature DCs, while these were unchanged in THP1-derived mature DCs (Figures 3F, G; Supplementary Figure S9). The knockdown of IFT88 also decreased proliferation activity promoted by co-stimulation with PDGF-A and GM-CSF (Figure 3H), suggesting a role of IFT88 in the PDGFRα signaling regulation in DC proliferation. It has been reported that treatment with 4 mMchloral hydrate (CH) suppresses primary cilia formation (Masyuk et al., 2006). Treatment of primary monocyte-derived immature DCs with 4 mM CH increased the population of CCR7high, suggesting that inhibition of primary cilium formation promotes DC maturation (Supplementary Figure S10).

### Primary cilium and immature LCs are increased in the atopic dermatitis epidermis

**FIGURE 4:**
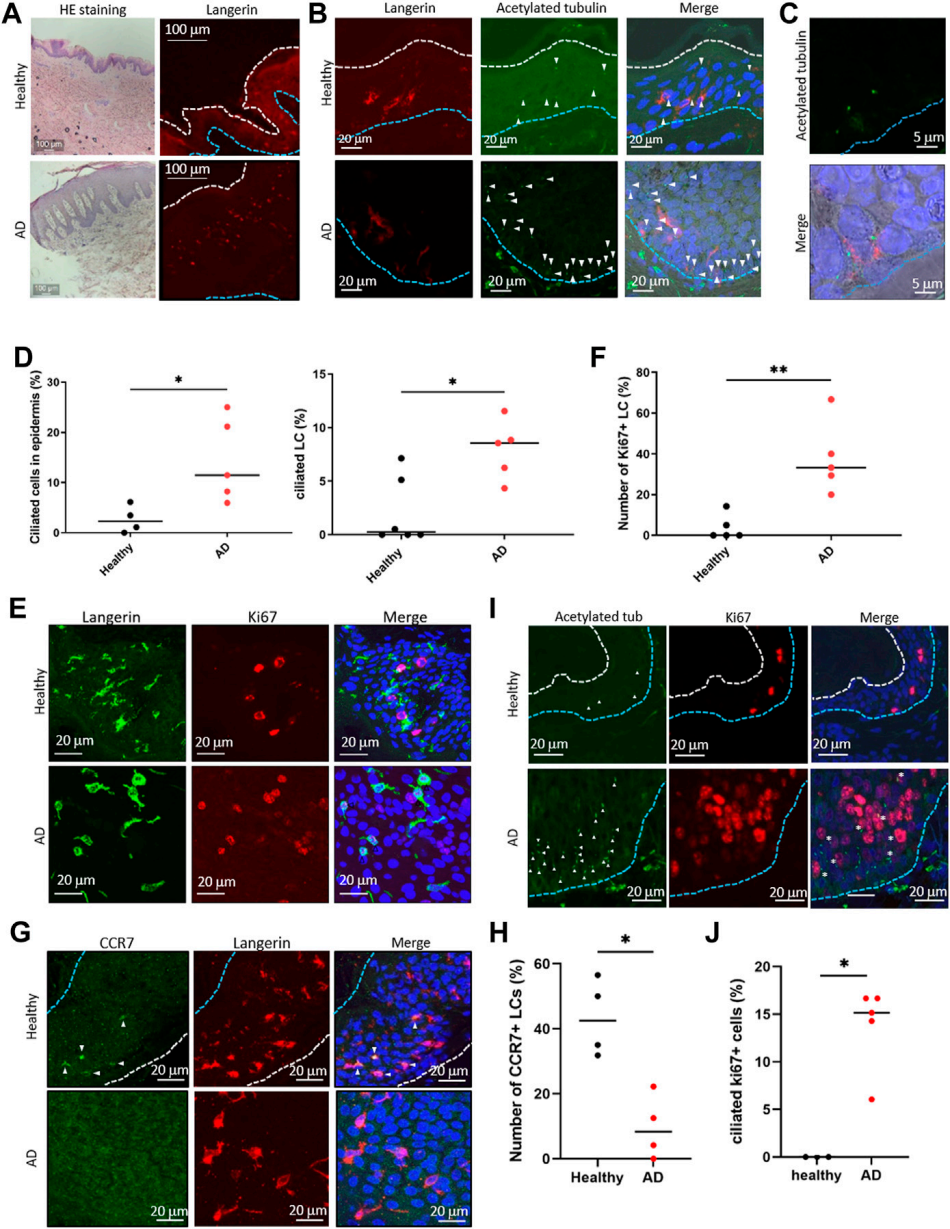
**(A)** (Left) Representative image of HE staining of the human epidermis. (Right) Representative image of langerin in the epidermis. The blue dotted line indicates the basal membrane. The white dotted line indicates the stratum corneum. Three biological replicates (3 donors) were examined. The upper panels show healthy skin. The lower panels show atopic dermatitis (AD) skin. Scale bar: 100 μm. **(B)** Langerin (red) and acetylated tubulin (green) were immunostained in the human epidermis. Nuclei were stained with hoechst 33342 (blue). The blue dotted line indicates the basal membrane. The arrow indicates the primary cilium. Scale bar: 20 μm. Four biological replicates (4 donors) for healthy samples and 5 biological replicates (5 donors) for AD samples were examined. **(C)** Magnified image of primary ciliated LCs in the AD patient epidermis. Langerin is shown in red. Acetylated tubulin is shown in green. Nuclei were stained with hoechst 33342 (blue). Scale bar: 5 μm. **(D)** (Left) The percentage of primary ciliated epidermal cells, and (right) the percentage of primary ciliated LCs in the healthy or AD epidermis. Healthy samples: n = 4 or n = 6, AD samples: n = 5. The bar indicates the median value. *, p < 0.05 (Mann-Whitney U test). **(E)** Representative images of langerin (green) and Ki67 (red) in the epidermis. Nuclei were stained with hoechst 33342 (blue). Scale bar: 20 μm. The number of observed cells and primary cilium are as follows: ciliated cells in healthy skin; 22 cilia/358 cells. ciliated cells in AD; 76 cilia/662 cells. ciliated LCs in healthy skin; 4 cilia/288 cells. ciliated LCs in AD; 15 cilia/179 cells. **(F)** Quantitation of data shown in **(E)**. Ki67-positive LCs in the healthy and AD epidermis were counted and graphed. Healthy samples: n = 5, AD samples: n = 5. The number of observed cells and primary cilium are as follows: healthy; 3 cilia/37 cells. AD; 17 cilia/42 cells. **, *p* < 0.01 (Mann-Whitney U test). **(G)** Immunostaining of CCR7 (green) and langerin (red) in the human epidermis. Nuclei were stained with hoechst 33342 (blue). The blue dotted line indicates the basal membrane. The white dotted line indicates the stratum corneum. The arrow indicates CCR7-positive LC. Four biological replicates (4 donors) were examined. Scale bar: 20 μm. **(H)** Quantitation of the data shown in panel G. CCR7-positive LCs in the healthy and AD epidermis were counted and graphed. Healthy individual samples: n = 4, AD skin samples: n = 4. The number of observed cells and primary cilium are as follows: healthy; 22 cilia/119 cells. AD; 6 cilia/66 cells. *, p < 0.05 (Mann-Whitney U test). **(I)** Immunostaining of acetylated tubulin (green) and Ki67 (red) in epidermis derived from healthy donors or AD patients. Nuclei were stained with hoechst 33342 (blue). The blue dotted line indicates the basal membrane. The white dotted line indicates the stratum corneum. The arrow indicates the primary cilium. The asterisk shows Ki67-positive primary ciliated cells. Scale bar: 20 μm. **(J)** Quantitation of the data shown in panel **(I)**. Acetylated tubulin-positive, Ki67-positive cells in the healthy and AD epidermis were counted and graphed. Healthy individual samples: n = 3,ADskin samples: n = 5. The number of observed cells and primary cilium are as follows: healthy; no cilium/23 cells. AD; 21 cilia/155 cells. *, *p* < 0.05 (Mann-Whitney U test).

LCs proliferate significantly in the epidermis of patients with AD (Chorro et al., 2009). To ask if the atopic condition alters primary cilium formation in LCs, we investigated the presence of primary cilium in the human AD epidermis. As widely reported, hyperkeratosis was found in the AD epidermis, and LCs were present (Figure 4A). When comparing the AD epidermis with the healthy epidermis, the frequency of primary cilium in both langerin-negative cells and LCs significantly increased, especially in the basal area (Figures 4B–D). To further examine the relationship between proliferation and primary cilium formation, we investigated the number of Ki67-positive proliferating cells in AD. The expression level of ki67 increases as the stage progresses from G1 phase and reaches ts highest level in the M phase. As reported previously, Ki67-positive LCs increased in AD (Chorro et al., 2009) (Figures 4E, F). We also found that the Ki67-positive population in langerinnegative cells was increased (Figure 4E). Most of the Ki67-positive cells were found in both the healthy and atopic epidermis, but not in the dermis (Supplementary Figure S11A).]

Given the proliferation activity and promotion of primary cilium formation in LCs in AD, we next investigated an LC maturation marker, CCR7, in the epidermis. In the healthy epidermis, nearly 40% (median) of LCs were positively stained with CCR7, although atopic LCs with CCR7 were significantly low (Figures 4G,H). We found that langerin-positive cells in the atopic dermis were positively stained with CCR7 (Supplementary Figure S11B). These results suggest that atopic epidermal LCs are immature with forming primary cilium. In addition, we found that some ciliated cells were positively stained with Ki67 in AD, although none were positive in healthy skin (Figures 4I, J). We could not determine the specific cell cycle phase that cells were in, however we suggested that the increase of proliferative cells with primary cilium is a pathological phenotype in AD (Figure 4G).

### The percentage of ciliated cells correlates with loricrin expression

**FIGURE 5:**
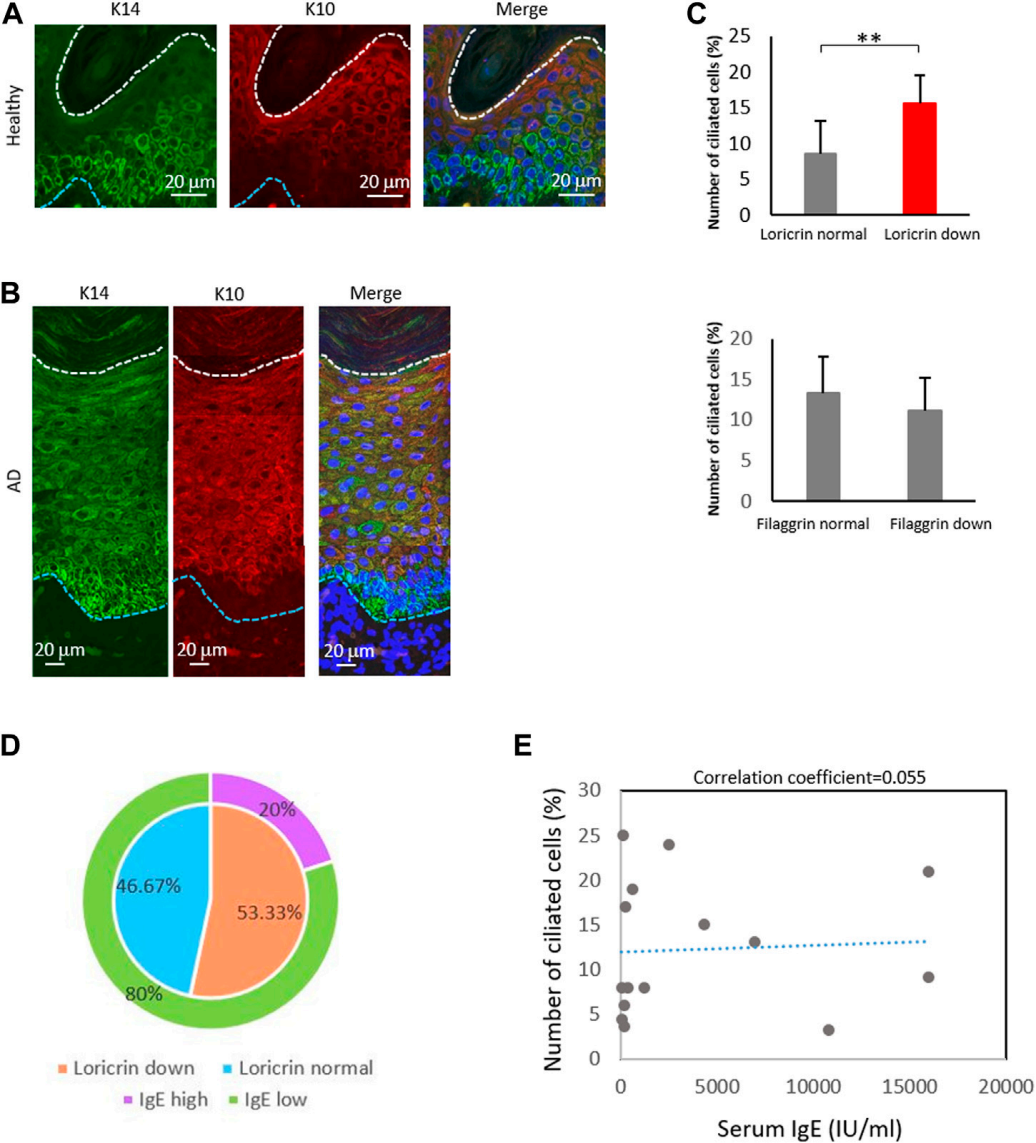
The percentage of ciliated cells negatively correlates with loricrin expression. **(A,B)** Immunostaining of K14 (green) and K10 (red) in **(A)** healthy epidermis, and in **(B)** atopic 975 epidermis. Nuclei were stained with hoechst 33342 (blue). The blue dotted line indicates the basal membrane. The white dotted line indicates the stratum corneum. Three biological replicates (3 donors) were examined. Scale bar: 20 μm. **(C)** Correlation between the number of primary ciliated cells and the epidermal barrier proteins loricrin (top) and filaggrin (bottom). AD epidermis samples derived from 15 patients were immunostained with either loricrin or filaggrin, with acetylated tubulin. Patients were classified into 2 groups based on the expression level of barrier proteins. The average percentages of primary ciliated cells in both groups were calculated. **, *p* < 0.01 (Student’s t-test). **(D)** The percentage of AD patients showing loricrin normal, loricrin low, IgE low, and IgE high. n = 15 donors. **(E)** Correlation between the number of ciliated cells and the serum IgE level in AD patients. n = 15 donors.

Given the high percentage of ciliated langerin-negative cells in the AD epidermis, we also explored the expression of KC maturation markers. In the healthy epidermis, K14, a marker for immature KCs, was highly expressed near the basal layer and stratum spinosum. In contrast, K10, a marker of mature KCs, was highly expressed only in the stratum granulosum (Figure 5A; Figures 1B,C). In the atopic epidermis, in contrast, K14 was highly expressed in the basal layer and moderately expressed throughout the stratum spinosum and stratum granulosum (Figure 5B). K10 was strongly expressed even in the stratum spinosum, along with K14 (Figure 5B).

We next investigated the other markers of KC differentiation, loricrin and filaggrin, by immunostaining (Supplementary Figure S12). These proteins are necessary for skin barrier formation, and both are decreased in more than 20% of AD patients, leading to barrier disruption and increasing the risk of AD (Palmer et al., 2006; Kim et al., 2008; O’Regan et al., 2008). We observed that the percentage of ciliated cells was significantly increased in patients with low levels of loricrin, but was not correlated with filaggrin expression (Figure 5C). Interestingly, when we analyzed the correlation between IgE levels and loricrin expression, as well as between IgE levels and primary cilium formation rate, there were no correlations found (Figures 5D, E). Because IgE levels generally correlate with allergic severity, our data suggest that the abnormal formation of primary cilium is the cause of the decrease in loricrin in AD, but is not the result of allergic responses. In summary, our immunostaining results suggested excessive proliferation and abnormal primary cilium formation in the AD epidermis, which in turn caused hyperproliferation and sustenance of immature LCs and KCs in AD.

### LCs secrete chemokines at higher levels than KCs

**FIGURE 6:**
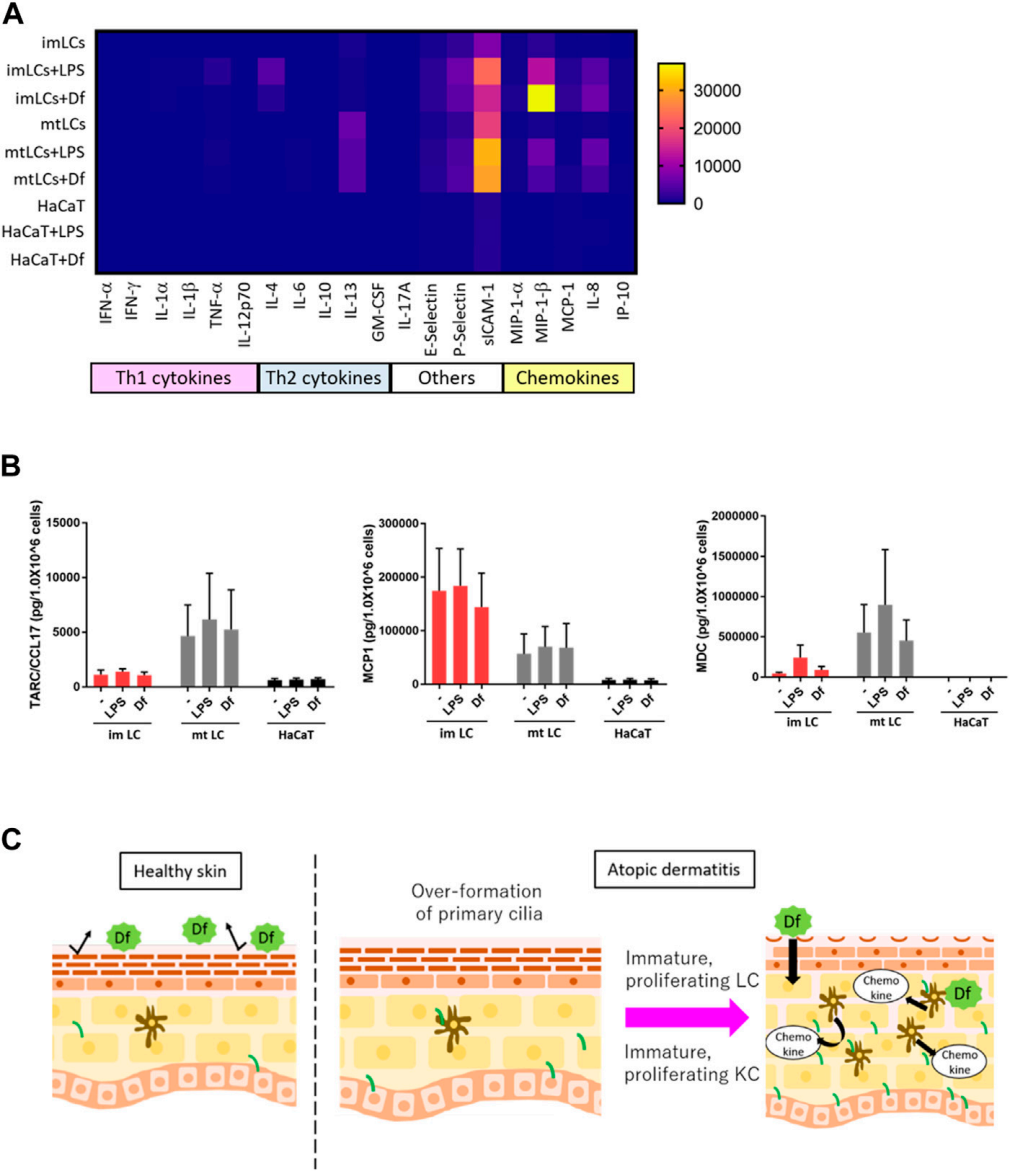
LCs secrete chemokines at higher levels than KCs. **(A)** Heatmap of cytokine and chemokine expression measured by a cytokine/chemokine multiplex array. CD14^+^ monocyte-derived immature LCs (imLCs), monocyte-derived mature LCs (mtLCs) and HaCaT were stimulated with 10 μg/mL LPS or 10 μg/mL *Df* for 24 h. **(B)** Average levels of Th2 chemokine expression. (Left) MCP1 (middle) TARC/CCL17, and (right) MDC were measured by ELISA. Cells were stimulated with 10 μg/mL LPS and 10 μg/mL *Df* in media containing 0.5% FBS for 24 h. Mixtures of culture supernatant and cell lysate were used for the assay. Error bar: SEM. n = 5 donors. **(C)** Graphical model based on this research. In healthy conditions, the epidermal barrier protects from allergens. In atopic dermatitis, primary cilium formation is promoted by unknown factors. Excessive formation of primary cilium induces epidermal cell proliferation, leading to immature conditions, which exacerbate disease; immature proliferating LCs secrete higher levels of cytokines than mature LCs. Primary ciliated KCs show an immature state, leading to epidermal barrier disruption.

Our findings suggested that ciliated cDCs and LCs were immature and were highly proliferating. We next asked the physiological relevance of immature LCs staying in the epidermis, and whether immature LCs secrete Th2 cytokines or chemokines to recruit Th2 cells. Th2 T cells strongly infiltrate the atopic epidermis and secrete Th2 cytokines (Nedoszytko et al., 2014). To examine cytokine and chemokine secretion in epidermal cells, we performed multiple cytokine/chemokine arrays. We stimulated monocyte-derived immature LCs, monocyte-derived mature LCs, and HaCaT with *Df* or LPS and analyzed cytokine/chemokine profiles (Figure 6A). We found that, in general, LCs expressed Th2 cytokines, adhesion molecules and chemokines at higher levels than HaCaT (Figure 6A). Some of these cytokines and chemokines are highly expressed in AD (Kimata and Lindley, 1994; Kaburagi et al., 2001; Wolkerstorfer et al., 2003). The expression of Th1 cytokines in all cells was relatively low compared to Th2 cytokines, adhesion molecules and chemokines (Figure 6A).

As we found that LPS and *Df* stimulation promoted the expression of IL-4, E-selectin, P-selectin, sICAM-1, MIP-1β, and IL-8 in LCs, we speculated that extracellular stimuli might promote the expression of other biomarkers for AD, including MDC, TARC, and MCP1(Fujii et al., 2004; Holm et al., 2021). We employed a single ELISA assay to detect these chemokines, and we found that MCP1 was highly secreted by immature LCs relative to mature LCs and HaCaT, however the difference was not statistically significant (Figure 6A). In contrast, TARC/CCL17 was expressed by mature LCs, but not so much by immature LCs or HaCaT (Figure 6B). MDC was highly expressed in both immature and mature LCs but was not detected in HaCaT (Figure 6C). These data suggest the important role of LCs but not KCs in secreting Th2 cytokines and chemokines to recruit Th2 cells. In the atopic epidermis, immature LCs were highly prevalent (Figure 4H). Our results raise the possibility that immature LCs are the main releasers of some Th2 chemokines in AD, which exacerbate disease. Of note, a related manuscript and data from our group were uploaded to the preprint server BioRxiv (Toriyama et al., 2020).

## Discussion

We identified primary cilium in the human epidermis and in immune cells derived from human PBMCs (Figure 1), and primary cilium formation in the epidermis was greatly increased in AD (Figure 4). Ki67-positive ciliated cells were highly prevalent in the AD epidermis (Figures 4I, J), so we hypothesized a relationship between primary cilium formation and epidermal cell proliferation in AD. Primary cilium formation is generally inhibited in the G2/M and S phases (Goto et al., 2013), and as expected, we did not find Ki67-positive ciliated cells in the healthy epidermis (Figures 4I, J). These interesting findings raised the possibility that regulation mechanisms for ciliogenesis, cell cycle and proliferation may be abnormal in AD and might represent a pathological phenotype of AD.We used TEM to investigate whether primary cilium is formed in PBMCs. Although structures resembling ciliary vesicles were observed (Figure 1H), structures such as ciliary sheath, ciliary pocket, and ciliary membrane were not observed. Instead, structures resembling elongated centrioles, which are often observed in cancer cells, were observed (Figure 1H) (Marteil et al., 2018). It has been reported that elongated centrioles are positive for centrin by immunostaining (Marteil et al., 2018). In our immunostaining images, pericentrin did not appear to be elongated structure. Therefore, the structures detected by acetylated tubulin were considered to correspond to the axoneme of the primary cilium and suggested to have a different structure from elongated centrioles. However, it is still unknown whether the structures confirmed by TEM protrude outside the cell, and this needs to be elucidated in future studies. If the receptors localized in the primary cilium membrane of DCs are identified, it may be possible to use antibodies recognizing these extracellular domains to determine whether primary cilium protrudes outside the cell.

To elucidate factors promoting primary ciliogenesis, we treated cDCs with IL-4, GM-CSF, LPS and *Df*. Our data suggested that GM-CSF, which is overproduced in atopic skin (Pastore et al., 1997; Rafatpanah et al., 2003), promoted primary cilium formation and proliferation in cDCs, however the increase of their median values of cDCs ciliation/proliferation was not as high as that in AD epidermis. We found an increase of between 0.68% (untreated) to 1.49% (GM-CSF treated) in ciliated cDCs, and a 0.2-fold increase in GM-CSF treated cDC proliferation. However, the percentages of ciliated LCs were 8.5% (AD) and 0.26% (healthy), and the percentages of Ki67-positive LCs were 33.3% (AD) and 0% (healthy), respectively (Figures 2E, F; Figure 4D). The non-significant increase of the median cDC percentages might have been caused by the use of pan-cDC rather than a specific cell population of cDCs responding to GM-CSF in our analysis. We have not answered the question of which population of cDCs could respond to GM-CSF to form primary cilium. Other characteristics of ciliated cDCs, for example, stemness marker expression and/or specific marker expression to determine isotypes, are unknown. Clarification of ciliated cDC characteristics by single-cell RNA sequencing will be carried out in the future.

To address whether extracellular stimuli induce primary cilium formation, we stimulated cDCs and HaCaT with LPS and *Df*. *Df* is a major allergen in house dust and is capable of inducing severe AD (Katoh et al., 2004; Oshio et al., 2009). Stimulation of human skin-derived DCs with LPS upregulated IL-12 mRNA and the secretion of IFNγ and IL-13, which are upregulated in AD (Leyva-Castillo et al., 2021). Other reports indicated that *Staphylococcus aureus* infections activate TLR4, which worsens AD (Takeuchi et al., 1999; Kobayashi et al., 2015; Ogonowska et al., 2020). Contrary to our expectations, these stimuli did not promote ciliogenesis and proliferation in cDCs (Figures 3A–D), although HaCaT proliferation increased with *Df* treatment (Supplementary Figure S7). These results suggested that *Df* may induce keratosis without ciliogenesis in KCs. As our ELISA experiments suggested that HaCaT produce PDGF-A constitutively (Supplementary Figure S8A), *Df* penetration to the epidermis may induce LC proliferation by stimulating PDGF-A production by overproliferated KCs. We have not identified the trigger of AD directly, however, we propose that GM-CSF, *Df* and PDGF-A are candidates to initiate AD by regulating primary cilium formation and proliferation of epidermal cells.

Interestingly, the prevalence of AD and asthma in ciliopathy patients, along with other phenotypes caused by primary cilium defects, is higher than in normal cohorts (Moore et al., 2005; Aldahmesh et al., 2014). An interesting report about the relationship between Bardet-Biedl Syndrome (BBS) and autoimmune disease development was published recently (Tsyklauri et al., 2021). BBS is one of the typical ciliopathies caused by mutations in cilium-related genes. In the literature, the prevalence of autoimmune diseases, including arthritis and type 1 diabetes mellitus, was significantly higher among individuals with BBS compared to the healthy population. The prevalence of psoriasis was also slightly, but not significantly, increased (*p* = 0.06) (Tsyklauri et al., 2021). This report showed no significant difference in the number of lymphocytes, monocytes and eosinophils between BBS patients and a healthy cohort, although immature B cell populations were increased in BBS model mice. This finding still raises an enigmatic question of why BBS patients, with minimal differences in immune cell numbers, frequently developed autoimmune diseases. Future investigations are required to answer this question.

Another recent finding clarified that single nucleotide polymorphisms (SNPs) in *KIF3A*, which is required for primary cilium maintenance, are associated with AD (Stevens et al., 2020). In this report, *KIF3A*-deficient mice had skin barrier dysfunction leading to AD (Stevens et al., 2020). Although the primary cilium distribution was not determined in previous reports in AD patients and in mice, it is known that *KIF3A* disruption usually leads to the disassembly of primary cilium (Lin et al., 2003). There is a discrepancy between this report and our data showing that primary cilium formation is elevated in the AD epidermis. Therefore, it is important to note the limitations of our study. We have not evaluated the physiological or pathological function(s) of primary cilium in the epidermis. The signaling pathway through cilium, as well as the signaling strength, should be investigated using an *in vivo* model in the future.

We found that primary cilium was abundantly observed in the basal layer, suggesting a relationship between KC proliferation, maturation and cilium formation (Figures 1B, C; Figure 5C). We attempted to investigate the function of primary cilium in KCs as in cDCs, however we could not study the function of primary cilium in KCs in detail due to technical challenges. Unexpectedly, we found that the purchased HaCaT and the primary KCs were not ciliated *in vitro*, although the epidermal KCs in donor tissue were ciliated (data not shown; Figure 1; Figure 4). The differences between epidermal KCs and isolated KCs are unknown, however, 3D cell-cell interaction, the expression level of primary cilium markers, culture media conditions, cell densities, or endogenous environmental factors including growth factors or inflammatory agents, may be involved in primary cilium formation in KCs. We also have not identified genetic alterations of cilium-related genes other than *KIF3A* in AD patients, so further experiments will be required.

Proliferation and maturation are highly inversely correlated. We demonstrated that the knockdown of IFT88 in THP1-derived DCs promoted maturation by attenuating proliferation activity promoted by PDGFRα signaling (Figures 3F, H). These results raised a hypothesis that PDGFRα signaling in the primary cilium regulates cell proliferation and inhibits maturation. We tried to identify the expression of PDGFRα in both healthy and AD skin, however the experiment was unsuccessful (data not shown). Furthermore, we could not detect PDGFRα localization in the primary cilium in cDCs, even though these cells expressed PDGFRα as detected by Western blotting (Supplementary Figures S7B, S8B, S8D). Interestingly, we found constitutive expression of PDGF-A in HaCaT (Supplementary Figure S8A). This result raises the possibility that PDGF-A expression is higher in the AD epidermis because the number of KCs is increased.

The relationship between pathology and PDGF-A expression will be the focal point of our next research. Imatinib, a PDGFRα antagonist, treats asthma by decreasing mast cell activation in patients; imatinib also decreases MCP1 expression in mice (Berlin and Lukacs, 2005; Cahill et al., 2017). Although its effectiveness in the treatment of AD has not been elucidated, these reports suggest the importance of PDGFRα signaling in allergic disorders. Furthermore, methotrexate, a folate antagonist, successfully treated AD (Shaffrali et al., 2003; Zoller et al., 2008). Previously, it was reported that the folate metabolic pathway is required for primary cilium formation (Toriyama et al., 2017). These observations support our idea that the regulation of primary cilium or PDGFRα signaling could be utilized in the treatment of AD.

On the basis of our novel findings here and of our previous report (Toriyama et al., 2020), we propose that primary cilium in LCs or in KCs has an important role in skin homeostasis by regulating proliferation, and elevated primary cilium formation may cause AD. Excessive primary cilium formation in KCs sustains immaturity, impairing barrier function by reducing loricrin expression. While this article was being prepared, we reported an increase of primary cilium in AD epidermis and suggested that primary cilium regulated KC maturation (Rizaldy et al., 2021). Besides KCs, ciliated immature LCs strongly recognize antigens and immunostimulants that pass through the skin barrier, leading LCs to secrete GM-CSF. GM-CSF then promotes primary cilium formation in LCs with autocrine/paracrine regulation, leading to strong proliferation signals such as PDGF. Chemokines secreted from immature LCs strongly recruit Th2 T cells, which causes a vicious cycle of AD (Figure 6D). Overall, our findings indicate that primary cilium functions in maintaining skin homeostasis.

## Materials and methods

### Human skin biopsy samples

Biopsy specimens were obtained from 15 adult patients diagnosed with AD and from 5 healthy donors. All donors were over 20 years of age, and provided written informed consent prior to sampling according to the Declaration of Helsinki. This study was approved by the institutional ethics committees of Nagoya City University Graduate School of Medical Sciences and of Osaka University. The approved protocol number is 60-18-0003.

### Isolation of primary cDCs and CD14^+^ monocytes

Human whole peripheral blood was purchased from the Japanese Red Cross Society according to the *Guidelines on the Use of Donated Blood in R&D, etc* *.* Blood from 103 healthy donors was obtained into a heparinized syringe. To isolate PBMCs, blood samples were diluted with an equal volume of PBS. A 35-mL aliquot of diluted blood was layered over 15 mL of Ficoll-Paque Plus density gradient medium (Cytiva) in Leucosep 50-mL tubes (Greiner Bio-One). The blood mixture was centrifuged at 500 *g* for 20 min at room temperature without braking. The PBMC fraction was carefully collected with a pipet, then washed with Roswell Park Memorial Institute 1640 (RPMI 1640) media supplemented with 10% FBS. After centrifugation at 500 *g* for 10 min at room temperature, red blood cells were lysed with Ammonium-Chloride-Potassium (ACK) buffer (150 mM NH_4_Cl, 10 mM KHCO_3_, 0.1 mM EDTA in PBS) in a conical tube at room temperature for 10 min. After washing of PBMC with RPMI 1640 containing 10% FBS, CD14^+^ monocytes were isolated with magnetic CD14 microbeads, for human (130-050-201, Miltenyi Biotec). The CD14^+^ fraction was used as CD14^+^ monocytes. cDCs were isolated with a magnetic Blood Dendritic Cell Isolation Kit II for human (130-091-379, Miltenyi Biotec), from the CD14-negative fraction.

### Cell culture

HaCaT were cultured in DMEM supplemented with 10% FBS, 1% penicillin and 1% streptomycin. THP1 were maintained in RPMI 1640 supplemented with 10% FBS, 1% penicillin and 1% streptomycin. HaCaT and THP1 were passaged every 3 days when they reached 80% confluence.

Immature and mature LCs were differentiated from CD14^+^ monocytes. To induce immature LCs, CD14^+^ monocytes were cultured in RPMI 1640 supplemented with 10 ng/mL IL-4 (100-09, Shenandoah), 100 ng/mL GM-CSF (100-08, Shenandoah), and 10 ng/mL TGFβ (240-B, R&D Systems) for 5 days. To induce mature LCs, CD14^+^ monocytes were cultured for 5 days in RPMI 1640 supplemented with 10 ng/mL IL-4, 100 ng/mL GM-CSF, 10 ng/mL TGFβ, and 20 ng/mL TNFα (MAN0003622, Gibco). A half volume of fresh medium was added every 2 days.

In order to visualize Arl13B-GFP positive primary cilium, primary cDCs were electroporated with Arl13B-GFP plasmids by using the Neon Transfection System (Thermo Fisher Scientific; protocol settings were: pulse voltage 1500 V, pulse width 30 ms, pulse number 1). Cells were cultured for 2 days in antibiotic-free RPMI 1640 media containing 10% FBS, and GFP signals were detected after immunostaining with a GFP antibody.

### Isolation of monocytes, pDCs, cDCs, CD4^+^ T cells, CD8^+^ T cells, NK cells and B cells by flow cytometry

To isolate pDCs, cDCs, and monocytes, human PBMCs were labeled with Alexa 647 anti-human CD11c (301620, clone 3.9, BioLegend), anti-human HLA-DR (Class III) PE-Texas conjugate (MHLDR17, Life Technology), BV421 anti-human CD14 (325627, clone HCD14, BioLegend), PE-Cy7 anti-human CD123 (560826, clone 7G3, BD Biosciences), APC-Fire 750 anti-human CD8a (301065, clone RPA-T8, BioLegend), APC-Fire 750 anti-human CD20 (302357, clone 2H7, BioLegend), APC-H7 anti-human CD3 (560275, clone SK7, BD Pharmingen) and Live/Dead Fixable Aqua Dead Cell Stain Kit (L34966, Invitrogen). Labeled cells were fixed with 4% PFA in PBS for 5 min, then cell marker expression was analyzed and cells were isolated with BD FACSAria II (BD Biosciences). The gating strategy is shown in Supplementary Figure S4.

To isolate CD4^+^ T, CD8^+^ T, NK, and B cells, human PBMCs were labeled with APC-H7 anti-human CD3 (560275, BD Bioscience), PE-Cy7 anti-human CD4 (317413, Biolegend), PE anti-human CD56 (355503, Biolegend), FITC anti-human CD8 (300905, Biolegend), APC anti-human CD19 (302211, Biolegend), and Live/Dead Fixable Aqua Dead Cell Stain Kit (L34966, Invitrogen). Labeled cells were fixed with 4% PFA in PBS for 5 min, then expression markers were analyzed and cells were isolated with BD FACSAria II (BD Biosciences). The gating strategy is shown in Supplementary Figure S4.

### Knockdown of *IFT88* in THP1-derived DCs

THP1 cells were stimulated with100 ng/ml GM-CSF and 100 ng/mL IL-4 for 5 days to differentiate into immature DCs. For differentiation into mature DCs, THP1 cells were stimulated with 200 ng/ml GM-CSF, 100 ng/mL IL-4, 20 ng/mL TNFα, and 200 ng/mL ionomycin for 5 days. After 5 days of differentiation, cells were electroporated with 20 nM siRNA targeting human *IFT88* (Life Technologies), or siRNA Control (AM4611, Invitrogen) with the Neon Transfection System (Thermo Fisher Scientific). The protocol settings were: pulse voltage 1680 V, pulse width 20 ms, pulse number 1. The target sequences of siRNAs were as follows:

si*IFT88* (human) #1;

Sense: 5'-CCAAGUGUCAAUAAGCAAAtt

Antisense: 5'-UUUGCUUAUUGAACACUUGGaa

si*IFT88* (human) #2:

Sense: 5'-GGUAGCUAGUUGUUUCAGAtt;

Antisense: 5'-UCUGAAACAACUAGCUACCat.

Differentiated THP1 cells were labeled with FITC anti-human CD86 (374203, Biolegend), APC-Cy7 anti-human CCR7 (353211, Biolegend), APC anti-human CD11c (301620, Biolegend), Texas Red-PE anti-human HLA-DR (MHLDR17, Life Technologies), and Live/Dead Fixable Aqua Dead Cell Stain Kit (L34966, Invitrogen). Labeled cells were fixed with 4% PFA for 5 min, then cell markers were analyzed with a BD FACSAria II cell sorter (BD Biosciences). The gating strategy is shown in Supplementary Figure S9.

### ELISA

One day before the experiment, 2.0 × 10^5^ HaCaT cells were cultured in 48-well plates. Cells were stimulated with 10 μg/mL LPS or 10 μg/mL *Df* for 24 h in 100 µL DMEM containing 0.5% FBS for 24 h. In 96-well plates, 2.0 × 10^4^ monocyte-derived LCs were cultured and stimulated with 10 μg/mL LPS or 10 μg/mL *Df* for 24 h in 100 μL of RPMI 1640 containing 0.5% FBS for 24 h. After the 24-h stimulation, 0.5% Triton X-100 was added directly into the cell cultures and mixtures of cell suspension and lysate were collected. The mixtures were centrifuged at 14,000 rpm at 4°C for 5 min to remove cell debris. The mixtures of cell lysate and supernatant were used for the ELISA assay following the kit protocol.

Cytokine/chemokine multiplex array was performed using the Inflammation 20-Plex Human ProcartaPlex Panel (EPX200-12185-901, Invitrogen) and analyzed with a Bio-Plex 200 (BioRad) following manufacturer protocols. ELISA kits for CCL17 (DY364), MCP1 (DCP00), MDC (DMD00), IL-4 (D4050), and GM-CSF (DY215) were purchased from R&D Systems. PDGF-A ELISA kits (EHPDGF) were purchased from Thermo Fisher Scientific. Absorbance (excitation, 450 nm) was measured with a microplate reader (Infinite F200 Pro, Tecan). For reference, background absorbance was measured at 560 nm.

### Immunostaining

Paraffin-embedded human skin tissues were deparaffinized in xylene 2 times for 10 min each. The samples were then immersed in 100% ethanol, 95% ethanol, and 2 lots of deionized water for 10 min consecutively. After rehydration, antigens were retrieved. The samples were immersed in 1 mM EDTA in deionized water and boiled in a microwave for 15 min. Samples were then incubated with blocking buffer (10% FBS, 0.1% Triton X-100 in PBS) at room temperature for 1 h. PBMCs, cDCs, T cells, NK cells, and B cells isolated by FACS were fixed with 4% PFA for 30 min at room temperature. Cells were mounted on MAS-coated slide glass (Matsunami). Cells were incubated with blocking buffer (10% FBS, 0.1% Triton X-100 in PBS) at room temperature for 1 h. After blocking and permeabilization, all samples were incubated with primary antibody. The antibodies were as follows: anti-acetylated α-tubulin clone 6-11B-1 antibody (Sigma, T7451, 1/1,000 dilution), antipolyglutamylated tubulin clone GT335 antibody (AdipoGen, AG-20B-0020-C100, 1/1,000 dilution), anti-langerin clone EPR15863 antibody (Abcam, ab192027, 1/1,000 dilution), antivimentin clone EPR3776 antibody (Abcam, ab92547, 1/1,000 dilution), anti-Ki67 clone 8D5 antibody (Cell Signaling Technology, #9449, 1/1,000 dilution), anti-Ki67 clone SP6 antibody (Abcam, ab16667, 1/1,000 dilution), anti-K10 antibody (Covance, PRB-159P, 1/1,000 dilution), anti-K14 clone LL002 antibody (abcam, ab7800, 1/1,000 dilution), anti-CCR7 clone 150503 antibody (R&D Systems, MAB197, 1/100 dilution), antipericentrin antibody (Bethyl, A301-348A, 1/1,000 dilution), and anti-GFP clone B-2 antibody (Santa Cruz Biotechnology, SC-9996, 1/1,000 dilution).

All primary antibodies were diluted in PBS and incubated at 4 C overnight. After washing with wash buffer (0.1% Tween-20 in PBS) 3 times, secondary antibodies containing Hoechst 33342 (Thermo Fisher Scientific, H3570) were incubated for 2 h at room temperature. Secondary antibodies were as follows: Alexa 488-conjugated IgG (ab150077, ab150113), Alexa 594-conjugated IgG (ab150076, 150116); both were purchased from Thermo Fisher Scientific. After washing with wash buffer, samples were mounted on slide glass with the ProLong Gold antifade reagent (Thermo Fisher Scientific, P36934). Immunofluorescence images were captured with an Olympus confocal microscope FV1200 (Olympus) equipped with a ×100 objective lens, or a Carl Zeiss LSM980 or LSM710 confocal microscope (Carl Zeiss) equipped with 100x or ×60 objective lenses. Ciliated cells were counted and statistically analyzed using the GraphPad Prism 9 software.

### Proliferation assay

In 96-well plates, 3,000 HaCaT cells or 5,000 primary cDCs were cultured in 100 µL of medium containing 0.5% FBS, 10 µL of CCK8/WST-1 buffer (Cell Counting Kit-8, Dojindo), and stimulants. To measure the absorbance at 450 nm, we used a microplate reader (Infinite F200 Pro). For the reference, background absorbance was measured at 560 nm. We generated a calibration curve by measuring the absorbance of samples containing 3,000, 5,000, and 10,000 cells, respectively. We calculated the cell numbers in each samples by employing a standard curve and calculated the relative cell number by dividing by the untreated sample value.

### Western blotting

Western blotting was performed according to standard protocols (Toriyama et al., 2012; Toriyama et al., 2017). Cells were lysed with RIPA buffer (50 mM Tris-HCl (pH 7.5), 150 mM NaCl, 1% Non-idet P40 substitute, 0.5% Sodium deoxycholate, 0.1% SDS), then the lysate was transferred to a 10% SDS-PAGE polyacrylamide gel. After electrophoresis, proteins were transferred to a PVDF membrane followed by incubation with primary antibodies overnight at 4°C. The details of the antibodies are as follows: anti-IFT88 antibody (Proteintech, 13967-1-AP, 1/1000 dilution), anti-Ki67 clone 8D5 antibody (Cell Signaling Technology, #9449, 1/1000 dilution), anti-PDGFRα clone 16A1 antibody (Abcam, ab96569, 1/100 dilution), anti-β-Actin clone C4 antibody (Santa Cruz Biotechnology, sc-47778, 1/1000 dilution). Bands were visualized with an Amersham Imager 600 (GE Healthcare) after incubation with the ECL Western blotting detection reagent (BioRad, RPN2109) or ECL Prime (BioRad, RPN2232).

### Electron microscopy

Cells were fixed with 2% glutaraldehyde in 0.1 M phosphate buffer (PB), pH 7.4, at 4°C overnight. The fixed samples were washed 3 times with 0.1 MPB for 30 min each, and were post-fixed with 2% OsO_4_ in 0.1 MPB at 4 °C for 1 h. The samples were then dehydrated in a graded ethanol solution at 50% and 70% for 5 min each at 4 °C, at 90% for 5 min at room temperature, and 3 rounds of dehydration with 100% ethanol for 5 min each at room temperature. The samples were infiltrated with propylene oxide (PO) 2 times for 5 min each and put into a 70:30 mixture of PO and resin (Quetol-812; Nisshin EM Co.) for 10 min. Then, the samples were exposed to the open air overnight to allow the PO to evaporate. The samples were transferred to fresh 100% resin and polymerized at 60 °C for 48 h. The polymerized resins were ultrathin-sectioned at 70 nm with a diamond knife on an ultramicrotome (Ultracut UCT; Leica). The sections were then mounted on copper grids. They were stained with 2% uranyl acetate at room temperature for 15 min, washed with distilled water, and then secondary-stained with lead stain solution (Sigma-Aldrich) at room temperature for 3 min. The grids were observed with a transmission electron microscope (JEM-1400 Plus; JEOL Ltd.) at an acceleration voltage of 100 kV. Digital images (3296 × 2472 pixels) were taken with a CCD camera (EM-14830RUBY2; JEOL Ltd.).

## Data Availability

The original contributions presented in the study are included in the article/Supplementary Material, further inquiries can be directed to the corresponding authors.

## Abbreviations

AD, atopic dermatitis; cDCs, conventional DCs; CD68, cluster of differentiation 68; CCR7, C-C chemokine receptor type 7; DCs, dendritic cells; Df, Dermatophagoides farina; HaCaT, human keratinocyte cell lines; IFT, intraflagellar transport; KCs, keratinocytes; LCs, Langerhans cells; MCP1/CCL2, monocyte chemotactic protein 1; MDC/CCL22, macrophage-derived chemokine; PBMCs, peripheral blood mononuclear cells; pDCs, plasmacytoid dendritic cells; PGE2, prostaglandin E2; PDGF-A, platelet-derived growth factor A; RPMI 1640, Roswell Park Memorial Institute 1640; TARC/CCL17, thymus and activation-regulated chemokine.
