# Corrigendum: Dendritic cell proliferation by primary cilium in atopic dermatitis

**DOI:** 10.3389/fmolb.2023.1215185

**Published:** 2023-08-30

**Authors:** Manami Toriyama, Defri Rizaldy, Motoki Nakamura, Yukiko Atsumi, Michinori Toriyama, Fumitaka Fujita, Fumihiro Okada, Akimichi Morita, Hiroshi Itoh, Ken J. Ishii

**Affiliations:** ^1^ Graduate School of Pharmacological Sciences, Osaka University, Osaka, Japan; ^2^ Center for Vaccine and Adjuvant Research (CVAR), National Institutes of Biomedical Innovation, Health and Nutrition, Osaka, Japan; ^3^ Graduate School of Science and Technology, Nara Institute of Science and Technology, Nara, Japan; ^4^ School of Pharmacy, Institut Teknologi Bandung, Bandung, Indonesia; ^5^ Department of Geriatric and Environmental Dermatology, Graduate School of Medical Sciences, Nagoya City University, Nagoya, Japan; ^6^ School of Biological and Environmental Sciences, Kwansei Gakuin University, Hyogo, Japan; ^7^ Mandom Corporation, Osaka, Japan; ^8^ Division of Vaccine Science, The Institute of Medical Science, The University of Tokyo, Tokyo, Japan

**Keywords:** primary cilium, langerhans cell, dendritic cell, keratinocytes, atopic dermatitis, pdgf signaling

In the published article, “Ginhoux, F. et al. (2006) Langerhans cells arise from monocytes *in vivo*, Nature Immunology, 7(3), 265–273. 10.1038/ni1307” “Hoeffel, G. et al. (2012) Adult Langerhans cells derive predominantly from embryonic fetal liver monocytes with a minor contribution of yolk sac-derived macrophages, The Journal of experimental medicine, 209(6), 1167–1181. 10.1084/jem.20120340” was not cited in the article. The citation has now been inserted in the **Introduction** and should read:

It is reported that LCs are derived from fetal liver, yolk sac and bone morrow, while DCs in the blood are derived from bone marrow in mice, and both share similar functions (Ginhoux et al., 2006; Hoeffel et al., 2012; **Haase and Nestle, 2014**). In the published article, “Marteil, G. et al. (2018) Over-elongation of centrioles in cancer promotes centriole amplification and chromosome missegregation, Nature Communications, 9(1), 1258. 10.1038/s41467-018-03641-x” was not cited in the article. The citation has now been inserted in the **Discussion**, Paragraph Number 2 and should read:

“We used TEM to investigate whether primary cilium is formed in PBMCs. Although structures resembling ciliary vesicles were observed (**Figure 1H**), structures such as ciliary sheath, ciliary pocket, and ciliary membrane were not observed. Instead, structures resembling elongated centrioles, which are often observed in cancer cells, were observed (**Figure 1H**) (Marteil et al., 2018). It has been reported that elongated centrioles are positive for centrin by immunostaining (Marteil et al., 2018).”

In the published article, “Masyuk, A.I. et al. (2006) Cholangiocyte cilia detect changes in luminal fluid flow and transmit them into intracellular Ca^2+^ and cAMP signaling, Gastroenterology, 131(3), 911–920. 10.1053/j.gastro.2006.07.003” was not cited in the article. The citation has now been inserted in **Results**, **Downregulation of the primary cilium component IFT88 decreases proliferation activity promoted by PDGFRα signaling in THP1-derived DCs**, Paragraph Number 3 and should read:

“It has been reported that treatment with 4 mM chloral hydrate (CH) suppresses primary cilia formation (Masyuk et al., 2006). Treatment of primary monocyte-derived immature DCs with 4 mM CH increased the population of CCR7^high^, suggesting that inhibition of primary cilium formation promotes DC maturation (Supplementary Figure S10).”

In the published article, there was an error in the legend for **Figure 1** as published. The corrected legend appears below.

“**FIGURE 1** | **(A)** Hematoxylin and eosin (HE) staining of healthy human skin. Scale bar: 100 μm. **(B,C)** Representative images of epidermal primary cilium-like structures (acetylated tubulin; green) in **(B)** the K14-positive basal layer (red), and in **(C)** the K10-positive stratum spinosum/granular layer (red). Nuclei were stained with hoechst 33342 (blue). The arrow indicates the primary cilium. The blue dotted line indicates the epidermal basement membrane. The white dotted line indicates the stratum corneum. The area within the dotted box was magnified and shown on the right. Five biological replicates (five individual donors) were examined. Scale bars: 20 μm and 2 μm, respectively. **(D)** The number of primary ciliated cells in the K14-positive basal layer and the K10-positive stratum spinosum/granular layer shown in **(C,D)**. *, *p* < 0.05 (Mann-Whitney U test). *n* = 5. K14-; 936 cells were observed and 2 cilia were identified. K14+; 1,248 cells were observed and 19 cilia were identified. K10-; 860 cells were observed and 22 cilia were identified. K10+; 1,203 cells were observed and 7 cilia were identified. **(E)** Representative immunostaining image of langerin-positive LCs (red) and primary cilium (acetylated tubulin; green) in healthy human epidermis. Nuclei were stained with hoechst 33342 (blue). The arrow indicates a primary cilium-like structure. The blue dotted line indicates the epidermal basement membrane. The white dotted line indicates the stratum corneum. The area within the dotted box was magnified and shown on the right. Scale bars: 10 μm and 2 μm, respectively. Five biological replicates (five individual donors) were examined, respectively. **(F)** Representative immunostaining image of acetylated tubulin or polyglutamylated tubulin (green) with centrosome (pericentrin; red) and nuclei (hoechst 33342; blue) in human PBMCs. The dotted box indicates the magnified area shown on the far right. Scale bars: 5 μm and 1 μm, respectively. Three biological replicates (3 donors) were examined. **(G)** Representative immunostaining image of human PBMCs expressing Arl13B-GFP (green). Nuclei were stained with hoechst 33342 (blue). Cells were electroporated with an Arl13B-GFP expression plasmid. Two days after transfection, cells were immunostained by using an anti-GFP antibody. The area within the dotted box was magnified and shown on the bottom. Scale bars: 5 μm and 2 μm, respectively. Three biological replicates (3 donors) were examined. **(H)** Electron microscope images of primary cilium-like structures in PBMCs. The top two pictures show representative ciliary vesicle-like structures. The bottom picture shows centrosome elongation resembling axoneme extension. For electron microscope analysis, five biological replicates (five donors) were examined. The arrow indicates a ciliary vesicle-like structure. The asterisk indicates the Golgi apparatus. The star indicates the nucleus. The area within the dotted box was magnified and shown on the right. Scale bars: 2 μm and 200 nm, respectively. **(I)** Monocytes, pDCs and cDCs were isolated from human PBMCs using flow cytometry, then cells were immunostained with acetylated tubulin (green), and pericentrin (red). Nuclei were stained with hoechst 33342 (blue). Three biological replicates (3 donors) were examined. The area within the dotted box was magnified and shown on the right. Scale bars: 5 μm and 1 μm, respectively. **(J)** Human cDCs isolated from PBMCs with magnetic beads were cultured in media supplemented with 10% FBS or 0.5% FBS (serum starvation) for 16 h. Cells were immunostained with acetylated tubulin (green) and pericentrin (red). Serum+; 1,772 cells were observed and 8 cilia were identified. Serum-; 1829 cells were observed and 25 cilia were identified. **(K)** The number of primary ciliated cDCs and the cilium length shown in **(J)** were measured. Thirteen biological replicates (13 donors) were examined respectively. The bar indicates the median value. *, *p* < 0.05 (Mann-Whitney U test).”

In the published article, there was an error in the legend for **Figure 2** as published. The corrected legend appears below.

“**FIGURE 2** | **(A)** CD14^+^ monocytes were isolated from human PBMCs with magnetic beads, then cells were differentiated into DCs by stimulating with 50 ng/ml GM-CSF, 50 ng/mL IL-4 and 50 ng/mL TNFα.The percentages of primary ciliated cells are shown in the graph. The bar indicates the median value. *, *p* < 0.05 (Kruskal-Wallis and Dunn’s multiple comparison). *n* = 10 biological replicates (10 donors). Day 0; 1950 cells were observed and 5 cilia were identified. The number of observed cells and primary cilium are as follows: Day 0; 5 cilia/1,950 cells. Day 1; 3 cilia/1,620 cells. Day 3; 0 cilium/2919 cells. Day 7; 0 cilium/989 cells. **(B,C)** Human cDCs isolated from PBMCs were stimulated with **(B)** TNFα or **(C)** PGE2 for 24 h. The percentages of ciliated cDCs are shown in the graph. The bar indicates the median value. *, *p* < 0.05, **, *p* < 0.01 (Kruskal-Wallis and Dunn’s multiple comparison). *n* = 12 biological replicates (12 donors) and *n* = 18 biological replicates (18 donors), respectively. The number of observed cells and primary cilium are as follows: 0 ng/mL TNFα; 14 cilia/1,518 cells. 0.1 ng/mL TNFα; 8 cilia/1,519 cells. 1 ng/mL TNFα; 5 cilia/1,407 cells. 10 ng/mL TNFα; 2 cilia/1,570 cells. 50 ng/mL TNFα; 2 cilia/1,487 cells. 0 nM PGE2; 15 cilia/1,605 cells. 0.1 nM PGE2; 6 cilia/1,553 cells. 1 nM PGE2; 6 cilia/1,628 cells. 10 nM PGE2; 5 cilia/1,524 cells. 50 nM PGE2; 2 cilia/1,551 cells. **(D)** CD14^+^ monocytes were isolated from human PBMCs with magnetic beads, then cells were differentiated into DCs by stimulating with 50 ng/ml GM-CSF and 50 ng/mL IL-4. The percentages of primary ciliated cells are shown in the graph. The bar indicates the median value. *, *p* < 0.05, ***, *p* < 0.001 (Kruskal-Wallis and Dunn’s multiple comparison). *n* = 12 biological replicates (12 donors). The number of observed cells and primary cilium are as follows: Day 0; 3 cilia/1,302 cells. Day 1; 15 cilia/1,541 cells. Day 3; 12 cilia/1,724 cells. Day 7; 11 cilia/1,596 cells. **(E)** cDCs isolated from PBMCs were stimulated with 50 ng/mL IL-4 and 50 ng/ml GM-CSF for 24 h. The percentages of primary ciliated cells were counted and graphed. The bar indicates the median value. *, *p* < 0.05, (Kruskal-Wallis and Dunn’s multiple comparison). *n* = 20 biological replicates (20 donors). **(F)** Five thousand cDCs isolated from PBMCs were cultured with 50 ng/mL IL-4, 50 ng/ml GM-CSF in RPMI 1640 containing 0.5% FBS and 10% CCK8/WST-1 buffer for 48 h. The cell number was calculated by measuring the absorbance. To generate the calibration curve, the absorbance values of 3,000, 5,000, and 10,000 cells were measured. Cell numbers in each sample were calculated from a standard curve, and relative cell numbers were calculated by dividing by the untreated sample value. The bar indicates the median value. **, *p* < 0.01 (Kruskal-Wallis and Dunn’s multiple comparison). *n* = 13 biological replicates (13 donors). **(G)** Average GM-CSF expression measured by ELISA. Immature LCs (imLCs), mature LCs (mtLCs) and HaCaT cells were stimulated with 10 μg/mL LPS or 10 μg/mL Df for 24 h in media containing 0.5% FBS. Mixtures of culture supernatant and cell lysate were used for the assay. The error bar shows the SEM. *n* = 5 biological replicates (5 donors). Kruskal-Wallis and Dunn’s multiple comparison were performed.”

In the published article, there was an error in the legend for **Figure 3** as published. The corrected legend appears below.

“**FIGURE 3** | IFT88 downregulation increases maturation marker expression with attenuating proliferation activity. **(A)** cDCs were stimulated with 1 μg/mL Df for 48 h. After immunostaining of acetylated tubulin, the percentage of primary ciliated cells was determined. *n* = 10 biological replicates (10 donors). The number of observed cells and primary cilium are as follows: no treatment; 8 cilia/1,074 cells. Df; 17 cilia/1,055 cells. The bar indicates the median value. **, *p* < 0.01 (Mann-Whitney U test). **(B)** cDCs were stimulated with 1 μg/mL LPS for 24 h. After immunostaining of acetylated tubulin, primary ciliated cDCs were counted and their numbers graphed. *n* = 15 biological replicates (15 donors). The number of observed cells and primary cilium are as follows: no treatment; 13 cilia/1,679 cells. LPS; 7 cilia/1687 cells. The bar indicates the median rate. *, *p* < 0.05, Mann-Whitney U test. **(C)** Five thousand cDCs were stimulated with 1 μg/mL Df for 48 h, then the cell number was calculated by the CCK8/WST-1 assay. To generate the calibration curve, the absorbance values of 3,000, 5,000, and 10,000 cells were measured. Cell numbers in each sample were calculated from the standard curve. Relative cell numbers were calculated by dividing by the untreated sample value. The bar indicates the median value. The Mann-Whitney U test was performed. *n* = 11 biological replicates (11 donors). **(D)** Five thousand cDCs were stimulated with 1 μg/mL LPS for 48 h, then relative cell numbers were calculated by performing the CCK8/WST-1 assay. The bar indicates the median value. The Mann Whitney U test was performed. *n* = 12 biological replicates (12 donors). **(E)** Five thousand cDCs were stimulated with 10 ng/mL PDGF-A or 10 ng/ml GM-CSF for 48 h. Relative numbers of cDCs were determined by the CCK8/WST-1 assay. For the negative control of PDGF-A stimulation, PDGF-A solvent (4 mM HCl, 0.1% BSA) was added. The bar indicates the median value. Kruskal-Wallis and Dunn’s multiple comparison was used for statistical analysis. *, *p* < 0.05, **, *p* < 0.01, *n* = 15 biological replicates (15 donors). **(F)** Representative expression of maturation markers in DCs. To differentiate immature DCs, THP1 cells were cultured with 100 ng/ml GM-CSF and 100 ng/mL IL-4 for 5 days. On day 5, cells were electroporated with 20 nM siRNA targeting IFT88 or control siRNA. Two days after electroporation, the expression of cell markers was analyzed by flow cytometry. *n* = 3 biological replicates. **(G)** Representative image of IFT88 in THP1-derived immature DC detected by Western blotting. *n* = 3 biological replicates (3 donors). **(H)** Immature DCs derived from THP1 were electroporated with 20 nM siRNA. Two days after electroporation, 5,000 cells were stimulated with 10 ng/mL PDGF-A or 10 ng/ml GM-CSF for 48 h. A CCK8/WST-1 assay was performed to calculate the cell number. The bar indicates the median value. **, *p* < 0.01 (one-way ANOVA, Tukey’s multiple comparison). *n* = 5 biological replicates.”

In the published article, there was an error in the legend for **Figure 4** as published. The corrected legend appears below.

“**FIGURE 4** | **(A)** (Left) Representative image of HE staining of the human epidermis. (Right) Representative image of langerin in the epidermis. The blue dotted line indicates the basal membrane. The white dotted line indicates the stratum corneum. Three biological replicates (3 donors) were examined. The upper panels show healthy skin. The lower panels show atopic dermatitis (AD) skin. Scale bar: 100 μm. **(B)** Langerin (red) and acetylated tubulin (green) were immunostained in the human epidermis. Nuclei were stained with hoechst 33342 (blue). The blue dotted line indicates the basal membrane. The arrow indicates the primary cilium. Scale bar: 20 μm. Four biological replicates (4 donors) for healthy samples and 5 biological replicates (5 donors) for AD samples were examined. **(C)** Magnified image of primary ciliated LCs in the AD patient epidermis. Langerin is shown in red. Acetylated tubulin is shown in green. Nuclei were stained with hoechst 33342 (blue). Scale bar: 5 μm. **(D)** (Left) The percentage of primary ciliated epidermal cells, and (right) the percentage of primary ciliated LCs in the healthy or AD epidermis. Healthy samples: *n* = 4 or *n* = 6, AD samples: *n* = 5. The bar indicates the median value. *, *p* < 0.05 (Mann-Whitney U test). **(E)** Representative images of langerin (green) and Ki67 (red) in the epidermis. Nuclei were stained with hoechst 33342 (blue). Scale bar: 20 μm. The number of observed cells and primary cilium are as follows: ciliated cells in healthy skin; 22 cilia/358 cells. ciliated cells in AD; 76 cilia/662 cells. ciliated LCs in healthy skin; 4 cilia/288 cells. ciliated LCs in AD; 15 cilia/179 cells. **(F)** Quantitation of data shown in **(E)**. Ki67-positive LCs in the healthy and AD epidermis were counted and graphed. Healthy samples: *n* = 5, AD samples: *n* = 5. The number of observed cells and primary cilium are as follows: healthy; 3 cilia/37 cells. AD; 17 cilia/42 cells. **, *p* < 0.01 (Mann-Whitney U test). **(G)** Immunostaining of CCR7 (green) and langerin (red) in the human epidermis. Nuclei were stained with hoechst 33342 (blue). The blue dotted line indicates the basal membrane. The white dotted line indicates the stratum corneum. The arrow indicates CCR7-positive LC. Four biological replicates (4 donors) were examined. Scale bar: 20 μm. **(H)** Quantitation of the data shown in panel G. CCR7-positive LCs in the healthy and AD epidermis were counted and graphed. Healthy individual samples: *n* = 4, AD skin samples: *n* = 4. The number of observed cells and primary cilium are as follows: healthy; 22 cilia/119 cells. AD; 6 cilia/66 cells. *, *p* < 0.05 (Mann-Whitney U test). **(I)** Immunostaining of acetylated tubulin (green) and Ki67 (red) in epidermis derived from healthy donors or AD patients. Nuclei were stained with hoechst 33342 (blue). The blue dotted line indicates the basal membrane. The white dotted line indicates the stratum corneum. The arrow indicates the primary cilium. The asterisk shows Ki67-positive primary ciliated cells. Scale bar: 20 μm. **(J)** Quantitation of the data shown in panel **(I)**. Acetylated tubulin-positive, Ki67-positive cells in the healthy and AD epidermis were counted and graphed. Healthy individual samples: *n* = 3, AD skin samples: *n* = 5. The number of observed cells and primary cilium are as follows: healthy; no cilium/23 cells. AD; 21 cilia/155 cells. *, *p* < 0.05 (Mann-Whitney U test).”

In the published article, there was an error in the legend for **Figure 5** as published. The corrected legend appears below.

“**FIGURE 5** | The percentage of ciliated cells negatively correlates with loricrin expression. **(A,B)** Immunostaining of K14 (green) and K10 (red) in **(A)** healthy epidermis, and in **(B)** atopic 975 epidermis. Nuclei were stained with hoechst 33342 (blue). The blue dotted line indicates the basal membrane. The white dotted line indicates the stratum corneum. Three biological replicates (3 donors) were examined. Scale bar: 20 μm. **(C)** Correlation between the number of primary ciliated cells and the epidermal barrier proteins loricrin (top) and filaggrin (bottom). AD epidermis samples derived from 15 patients were immunostained with either loricrin or filaggrin, with acetylated tubulin. Patients were classified into 2 groups based on the expression level of barrier proteins. The average percentages of primary ciliated cells in both groups were calculated. **, *p* < 0.01 (Student’s t-test). **(D)** The percentage of AD patients showing loricrin normal, loricrin low, IgE low, and IgE high. *n* = 15 donors. **(E)** Correlation between the number of ciliated cells and the serum IgE level in AD patients. *n* = 15 donors.”

In the published article, there was an error in the legend for **Figure 6** as published. The corrected **title** appears below.

“**FIGURE 6** | LCs secrete chemokines at higher levels than KCs.”

In the published article, there was an error in Supplementary Figures S1–S12. The Supplementary Material has been updated.

In the published article, there were a number of textual errors.

A correction has been made to **Introduction**, Paragraph Number 1. This sentence previously stated:

“KCs ultimately form the stratum corneum, which forms a physical barrier against pathogens (**Madison, 2003**). Immune cells such as the Langerhans cells (LCs), which have a similar role as the dendritic cells (DCs), also exist in the epidermis and maintain skin homeostasis by presenting antigens to activate T cells (**Pasparakis et al., 2014; Nestle et al., 2009; Deckers et al., 2018**). When LCs incorporate antigens, they immediately migrate toward the lymph nodes, then activate T cells (**Nestle et al., 2009; Pasparakis et al., 2014; Deckers et al., 2018**).”

The corrected sentence appears below:

“The outer layer of the epidermis is composed of layers of dead KCs, called the stratum corneum, which forms a brick-like physical barrier that protects the body from pathogens, toxins and other harmful substances in the environment (**Madison, 2003**). Immune cells such as the Langerhans cells (LCs), which have a similar role as the dendritic cells (DCs), also exist in the epidermis and maintain skin homeostasis by presenting antigens to activate T cells (**Nestle et al., 2009; Pasparakis, Haase and Nestle, 2014; Deckers, Hammad and Hoste, 2018**). It is reported that LCs are derived from fetal liver, yolk sac and bone morrow, while DCs in the blood are derived from bone marrow in mice, and both share similar functions (**Haase and Nestle, 2014**; Ginhoux et al., 2006; Hoeffel et al., 2012). When LCs incorporate antigens, they immediately migrate toward the lymph nodes, then activate T cells (**Nestle et al., 2009; Pasparakis, Haase and Nestle, 2014; Deckers, Hammad and Hoste, 2018**).”

In the published article, there was an error.

A correction has been made to **Introduction**, Paragraph Number 3. This sentence previously stated:

“The intraflagellar transport (IFT) system is essential for axonome elongation and ciliary protein transport (**Pedersen and Rosenbaum, 2008**).”

The corrected sentence appears below:

“The intraflagellar transport (IFT) system is essential for axoneme elongation and ciliary protein transport (**Pedersen and Rosenbaum, 2008**).”

In the published article, there was an error.

A correction has been made to **Results**, *Human primary immune cells can assemble a primary cilium-like structure*, Paragraph Number 1. This sentence previously stated:

“We also found primary cilium-like structures in the K14-positive epidermal basal area where proliferating keratinocytes are populous (**Figure 1B**). Primary cilium-like structures were also detected in the K10-positive stratum spinosum and the granular layer, but at a lower frequency than in the basal layer (**Figures 1C, D**).”

The corrected sentence appears below:

“We also found primary cilium-like structures in the K14-positive epidermal basal area where proliferating KCs are populous (**Figure 1B**). Primary cilium-like structures were also detected in the K10-positive stratum spinosum and the granular layer where differentiated and matured KCs are present, but at a lower frequency than in the basal layer (**Figures 1C, D**).”

In the published article, there was an error.

A correction has been made to **Results**, *Human primary immune cells can assemble a primary cilium-like structure*, Paragraph Number 2. This sentence previously stated:

“We found that the entire cytosol of dermal langerin-positive cells was strongly stained with an acetylated tubulin, showing like background, even though the primary cilium was detected in langerin-negative dermal cells (Supplementary Figure S2).”

The corrected sentence appears below:

“We found that the entire cytosol of dermal langerin-positive cells was strongly stained with an acetylated tubulin, even though the primary cilium was detected in langerin-negative dermal cells (Supplementary Figure S2).”

In the published article, there was an error.

A correction has been made to **Results**, *Human primary immune cells can assemble a primary cilium-like structure*, Paragraph Number 3. This sentence previously stated:

“Primary cilium function in immune cells has not been investigated, although some immune cells can assemble primary cilium *in vitro* (18). As our data suggested that primary LCs could assemble primary cilium in the healthy human epidermis, we next investigated if human dendritic cells in blood are ciliated. LCs are regarded as being similar to DCs in the epidermis and have a similar function as conventional DCs (cDCs). To investigate whether immune cells in the blood, especially cDCs, are ciliated, we isolated peripheral blood mononuclear cells (PBMCs), a mixture of immune cells, from human peripheral blood, then immunostained them with acetylated or glutamylated tubulin to visualize the primary cilium. We found that nearly 2% of PBMC cells had primary cilium-like structures, showing a single protrusion stained with stabilized tubulin that extended from the centrosome marker, pericentrin, of each cell (**Figure 1F**).”

The corrected sentence appears below:

“Primary cilium function in immune cells has not been investigated, although some immune cells can assemble primary cilium *in vitro* (**Prosser and Morrison, 2015**). As our data suggested that primary LCs could assemble primary cilium in the healthy human epidermis, we next investigated if human dendritic cells in blood are ciliated. LCs are regarded as being similar to DCs in the epidermis and have a similar function as conventional DCs (cDCs). To investigate whether immune cells in the blood, especially cDCs, are ciliated, we isolated peripheral blood mononuclear cells (PBMCs), a mixture of immune cells, from human peripheral blood, then immunostained them with acetylated or polyglutamylated tubulin to visualize the primary cilium. We found that nearly 2% of PBMC cells had primary cilium-like structures, showing a single protrusion stained with stabilized tubulin that extended from the centrosome marker, pericentrin, of each cell (**Figure 1F**).”

In the published article, there was an error.

A correction has been made to **Results**, *Human primary immune cells can assemble a primary cilium-like structure*, Paragraph Number 4. This sentence previously stated:

“To further investigate primary cilium-like structures in PBMCs, we used transmission electron microscopy. We observed a vesicle–centrosome interaction which resembles a ciliary vesicle (**Figure 1H**). Also, centrosome elongation resembling axoneme extension, which is found in early primary cilium elongation, was observed (**Figure 1H**). These results suggested that human PBMCs can assemble primary cilium.”

The corrected sentence appears below:

“Please note that endogenous Arl13B was not detected as a single linear structure in PBMCs and LCs even though acetylated tubulin was detected as like primary cilium-like structure in PBMC (data not shown). To further investigate primary cilium-like structures in PBMCs, we used transmission electron microscopy (TEM). We observed a vesicle–centrosome interaction which resembles a ciliary vesicle (**Figure 1H**). Also, centrosome elongation resembling axoneme extension, which is found in early primary cilium elongation, was observed (**Figure 1H**). Although no structures resembling a ciliary sheath have been observed so far, our immunostaining and TEM results suggested that human PBMCs can assemble primary cilium.”

In the published article, there was an error.

A correction has been made to **Results**, *cDC maturation decreases primary cilium formation*, Paragraph Number 3. This sentence previously stated:

“As we found that GM-CSF was expressed without any stimulation in monocyte-derived immature LCs, we suggested that immature LCs spontaneously expressed GM-CSF (**Figure 2G**). In contrast, IL-4 was not detected in monocyte-derived immature and mature LCs, while HaCaT expressed IL-4 spontaneously (Supplementary Figure S6). These results suggest that immature LCs in the epidermis are the main producers of GM-CSF.”

The corrected sentence appears below:

“As we found that GM-CSF was expressed without any stimulation in monocyte-derived immature LCs, we suggest that immature LCs spontaneously express GM-CSF (**Figure 2G**). In contrast, IL-4 was not detected in monocyte-derived immature and mature LCs, while HaCaT expressed IL-4 spontaneously (Supplementary Figure S6). These results suggest that immature LCs in the epidermis are the main producers of GM-CSF.”

In the published article, there was an error.

A correction has been made to **Results**, *Dermatophagoides farinae (Df) antigen tends to promote primary cilium formation in DCs*, Paragraph Number 2. This sentence previously stated:

“The proliferation marker Ki67 increased after stimulation with Df in HaCaT (Supplementary Figure S7B).”

The corrected sentence appears below:

“The proliferation marker Ki67 increased after stimulation with Df in HaCaT (Supplementary Figures S7B, C).”

In the published article, there was an error.

A correction has been made to **Results**, *Downregulation of the primary cilium component IFT88 decreases proliferation activity promoted by PDGFRα signaling in THP1-derived DCs*, Paragraph Number 2. This sentence previously stated:

“As expected from the proliferation activity of cDCs, stimulation with GM-CSF or PDGF-A individually did not change Ki67 expression, but co-treatment of GM-CSF and PDGF-A tended to increase it (Supplementary Figures S8B, D).”

The corrected sentence appears below:

“Cilium formation in cells were increased in GM-CSF treated group (Supplementary Figures S8D, E). Furthermore, a trend towards an increase in primary cilia formation rate was observed with GM-CSF + PDGF-A compared to unstimulated cells (Supplementary Figures S8D, E). HaCaT stimulated with GM-CSF or PDGF-A individually did not change Ki67 expression, but co-treatment of GM-CSF and PDGF-A tended to increase it although no significant differences were observed (Supplementary Figures S8F, G).”

In the published article, there was an error.

A correction has been made to **Results**, *Downregulation of the primary cilium component IFT88 decreases proliferation activity promoted by PDGFRα signaling in THP1-derived DCs*, Paragraph Number 3. This sentence previously stated:

“THP1 cells were differentiated into immature DCs by stimulating them with 100 ng/ml GM-CSF and 100 ng/mL IL-4, or were differentiated into mature DCs by stimulating them with 200 ng/ml GM-CSF, 100 ng/mL IL-4, 20 ng/mL TNFα, and 200 ng/mL ionomycin for 5 days. At day 3, cells were electroporated with siRNA and cultured for more 2 days, then maturation markers were analyzed by using flow cytometry (**Figure 3F**; Supplementary Figure S9). The knockdown of IFT88 increased DC maturation markers, cluster of differentiation 86 (CD86) and C-C chemokine receptor type 7 (CCR7) in THP1-derived immature DCs, while these were unchanged in THP1-derived mature DCs (**Figure 3F**; Supplementary Figure S9). In addition, we found that Ki67 expression was reduced in IFT88 downregulated cells (**Figure 3G**). The knockdown of IFT88 also decreased proliferation activity promoted by co-stimulation with PDGF-A and GM-CSF **(Figure 3H**), suggesting a role of PDGFRα signaling in the primary cilium in DC proliferation.”

The corrected sentence appears below:

“THP1 cells were differentiated into immature DCs by stimulating them with 100 ng/ml GM-CSF and 100 ng/mL IL-4, or were differentiated into mature DCs by stimulating them with 200 ng/ml GM-CSF, 100 ng/mL IL-4, 20 ng/mL TNFα, and 200 ng/mL ionomycin for 5 days. At day 3, cells were electroporated with siRNA and cultured for 2 more days, then maturation markers were analyzed by using flow cytometry (**Figure 3F**; Supplementary Figure S9). The knockdown of *IFT88* increased DC maturation markers, cluster of differentiation 86 (CD86) and C-C chemokine receptor type 7 (CCR7) in THP1-derived immature DCs, while these were unchanged in THP1-derived mature DCs (**Figures 3F, G**; Supplementary Figure S9). The knockdown of *IFT88* also decreased proliferation activity promoted by co-stimulation with PDGF-A and GM-CSF (**Figure 3H**), suggesting a role of IFT88 in the PDGFRα signaling regulation in DC proliferation. It has been reported that treatment with 4 mM chloral hydrate (CH) suppresses primary cilia formation (Masyuk et al., 2006). Treatment of primary monocyte-derived immature DCs with 4 mM CH increased the population of CCR7^high^, suggesting that inhibition of primary cilium formation promotes DC maturation (Supplementary Figure S10).”

In the published article, there was an error.

A correction has been made to **Results**, *Primary cilium and immature LCs are increased in the atopic dermatitis epidermis*, Paragraph Number 1. This sentence previously stated:

“To further examine the relationship between proliferation and primary cilium formation, we investigated the number of Ki67-positive proliferating cells in AD. Ki67 expression is highly increased in the S and M phases of the cell cycle. As reported previously, Ki67-positive LCs increased in AD (**Chorro et al., 2009**) (**Figures 4E, F**). We also found that the Ki67-positive population in langerin-negative cells was increased (**Figure 4E**). Most of the Ki67-positive cells were found in both the healthy and atopic epidermis, but not in the dermis (Supplementary Figure S10A).”

The corrected sentence appears below:

“To further examine the relationship between proliferation and primary cilium formation, we investigated the number of Ki67-positive proliferating cells in AD. The expression level of ki67 increases as the stage progresses from G1 phase and reaches its highest level in the M phase. As reported previously, Ki67-positive LCs increased in AD (**Chorro et al., 2009**) (**Figures 4E, F**). We also found that the Ki67-positive population in langerin-negative cells was increased (**Figure 4E**). Most of the Ki67-positive cells were found in both the healthy and atopic epidermis, but not in the dermis (Supplementary Figure S11A).]”

In the published article, there was an error.

A correction has been made to **Results**, *Primary cilium and immature LCs are increased in the atopic dermatitis epidermis*, Paragraph Number 2–3. This sentence previously stated:

“We found that langerin-positive cells in the atopic dermis were positively stained with CCR7 (Supplementary Figure S10B). These results suggest that atopic epidermal LCs are immature with forming primary cilium.

Unexpectedly, some ciliated cells were positively stained with Ki67 in AD, although none were positive in healthy skin (**Figures 4I, J**). Primary cilium is generally formed in the G0 or G1 phases of the cell cycle, and they are disassembled in proliferating cells especially in the G2 to M phases (**Goto et al., 2013**). We could not determine the specific cell cycle phase that cells were in, however we observed the strong expression of Ki67 even in ciliated epidermal cells in AD, suggesting that proliferative cells pathologically possess primary cilium in AD (**Figure 4G**).”

The corrected sentence appears below:

“We found that langerin-positive cells in the atopic dermis were positively stained with CCR7 (Supplementary Figure S11B). These results suggest that atopic epidermal LCs are immature with forming primary cilium. In addition, we found that some ciliated cells were positively stained with Ki67 in AD, although none were positive in healthy skin (**Figures 4I, J**). We could not determine the specific cell cycle phase that cells were in, however we suggested that the increase of proliferative cells with primary cilium is a pathological phenotype in AD (**Figure 4G**).”

In the published article, there was an error.

A correction has been made to **Results**, *The percentage of ciliated cells correlates with loricrin expression*, Paragraph Number 2. This sentence previously stated:

“We next investigated the other markers of KC differentiation, loricrin and filaggrin, by immunostaining (Supplementary Figure S11).”

The corrected sentence appears below:

“We next investigated the other markers of KC differentiation, loricrin and filaggrin, by immunostaining (Supplementary Figure S12).”

In the published article, there was an error.

A correction has been made to **Discussion**, Paragraph Number 1–2. This sentence previously stated:

“We identified primary cilium in the human epidermis and in immune cells derived from human PBMCs (**Figure 1**), and primary cilium formation in the epidermis was greatly increased in AD (**Figure 4**). Ki67-positive ciliated cells were highly prevalent in the AD epidermis (**Figures 4I, J**), so we hypothesized a relationship between primary cilium formation and epidermal cell proliferation in AD. Primary cilium formation is generally inhibited in the G2/M and S phases (**Goto et al., 2013**), and as expected, we did not find Ki67-positive ciliated cells in the healthy epidermis (**Figures 4I, J**). These interesting findings raised the possibility that regulation mechanisms for ciliogenesis, cell cycle and proliferation may be abnormal in AD and might represent a pathological phenotype of AD.”

The corrected sentence appears below:

“We identified primary cilium in the human epidermis and in immune cells derived from human PBMCs (**Figure 1**), and primary cilium formation in the epidermis was greatly increased in AD (**Figure 4**). Ki67-positive ciliated cells were highly prevalent in the AD epidermis (**Figures 4I, J**), so we hypothesized a relationship between primary cilium formation and epidermal cell proliferation in AD. Primary cilium formation is generally inhibited in the G2/M and S phases (**Goto et al., 2013**), and as expected, we did not find Ki67-positive ciliated cells in the healthy epidermis (**Figures 4I, J**). These interesting findings raised the possibility that regulation mechanisms for ciliogenesis, cell cycle and proliferation may be abnormal in AD and might represent a pathological phenotype of AD.We used TEM to investigate whether primary cilium is formed in PBMCs. Although structures resembling ciliary vesicles were observed (**Figure 1H**), structures such as ciliary sheath, ciliary pocket, and ciliary membrane were not observed. Instead, structures resembling elongated centrioles, which are often observed in cancer cells, were observed (**Figure 1H**) (**Marteil et al., 2018**). It has been reported that elongated centrioles are positive for centrin by immunostaining (**Marteil et al., 2018**). In our immunostaining images, pericentrin did not appear to be elongated structure. Therefore, the structures detected by acetylated tubulin were considered to correspond to the axoneme of the primary cilium and suggested to have a different structure from elongated centrioles. However, it is still unknown whether the structures confirmed by TEM protrude outside the cell, and this needs to be elucidated in future studies. If the receptors localized in the primary cilium membrane of DCs are identified, it may be possible to use antibodies recognizing these extracellular domains to determine whether primary cilium protrudes outside the cell.”

In the published article, there was an error.

A correction has been made to **Discussion**, Paragraph Number 7. This sentence previously stated:

“Proliferation and maturation are highly inversely correlated. We demonstrated that the knockdown of IFT88 in THP1-derived DCs promoted maturation by attenuating proliferation activity promoted by PDGFRα signaling (**Figures 3F–H**). These results suggest that PDGFRα signaling in the primary cilium regulates cell proliferation and inhibits maturation.”

The corrected sentence appears below:

“Proliferation and maturation are highly inversely correlated. We demonstrated that the knockdown of IFT88 in THP1-derived DCs promoted maturation by attenuating proliferation activity promoted by PDGFRα signaling (**Figures 3F, H**). These results raised a hypothesis that PDGFRα signaling in the primary cilium regulates cell proliferation and inhibits maturation.”

In the published article, there was an error.

A correction has been made to **Discussion**. This sentence previously stated:

“While this article was being prepared, an increase of primary cilium in AD epidermis was reported and the authors suggested that primary cilium regulated KC maturation (**Rizaldy et al., 2021**).”

The corrected sentence appears below:

“While this article was being prepared, we reported an increase of primary cilium in AD epidermis and suggested that primary cilium regulated KC maturation (**Rizaldy et al., 2021**).”

In the published article, there was an error.

A correction has been made to **Materials and methods**, *Immunostaining*, Paragraph Number 1. This sentence previously stated:

“The antibodies were as follows: anti-acetylated α-tubulin clone 6-11B-1 antibody (Sigma, T7451, 1/1,000 dilution), anti-langerin clone EPR15863 antibody (Abcam, ab192027, 1/1,000 dilution), anti-vimentin clone EPR3776 antibody (Abcam, ab92547, 1/1,000 dilution), anti-Ki67 clone 8D5 antibody (Cell Signaling Technology, #9449, 1/1,000 dilution), anti-Ki67 clone SP6 antibody (Abcam, ab16667, 1/1,000 dilution), anti-K10 antibody (Covance, PRB-159P, 1/1,000 dilution), anti-K14 clone LL002 antibody (abcam, ab7800, 1/1,000 dilution), anti-CCR7 clone 150503 antibody (R&D Systems, MAB197, 1/100 dilution), anti-pericentrin antibody (Bethyl, A301-348A, 1/1,000 dilution), and anti-GFP clone B-2 antibody (Santa Cruz Biotechnology, SC-9996, 1/1,000 dilution).”

The corrected sentence appears below:

“The antibodies were as follows: anti-acetylated α-tubulin clone 6-11B-1 antibody (Sigma, T7451, 1/1,000 dilution), anti-polyglutamylated tubulin clone GT335 antibody (AdipoGen, AG-20B-0020-C100, 1/1,000 dilution), anti-langerin clone EPR15863 antibody (Abcam, ab192027, 1/1,000 dilution), anti-vimentin clone EPR3776 antibody (Abcam, ab92547, 1/1,000 dilution), anti-Ki67 clone 8D5 antibody (Cell Signaling Technology, #9449, 1/1,000 dilution), anti-Ki67 clone SP6 antibody (Abcam, ab16667, 1/1,000 dilution), anti-K10 antibody (Covance, PRB-159P, 1/1,000 dilution), anti-K14 clone LL002 antibody (abcam, ab7800, 1/1,000 dilution), anti-CCR7 clone 150503 antibody (R&D Systems, MAB197, 1/100 dilution), anti-pericentrin antibody (Bethyl, A301-348A, 1/1,000 dilution), and anti-GFP clone B-2 antibody (Santa Cruz Biotechnology, SC-9996, 1/1,000 dilution).”

The authors apologize for this error and state that this does not change the scientific conclusions of the article in any way. The original article has been updated.
