# Supplementary material for: Dendritic cell proliferation by primary cilium in atopic dermatitis
Source: Front Mol Biosci. 2023 Apr 26;10:1149828. doi: 10.3389/fmolb.2023.1149828 (PMC10169737; doi:10.3389/fmolb.2023.1149828)
Supplement: Supplementary file 1 [file DataSheet1.docx]

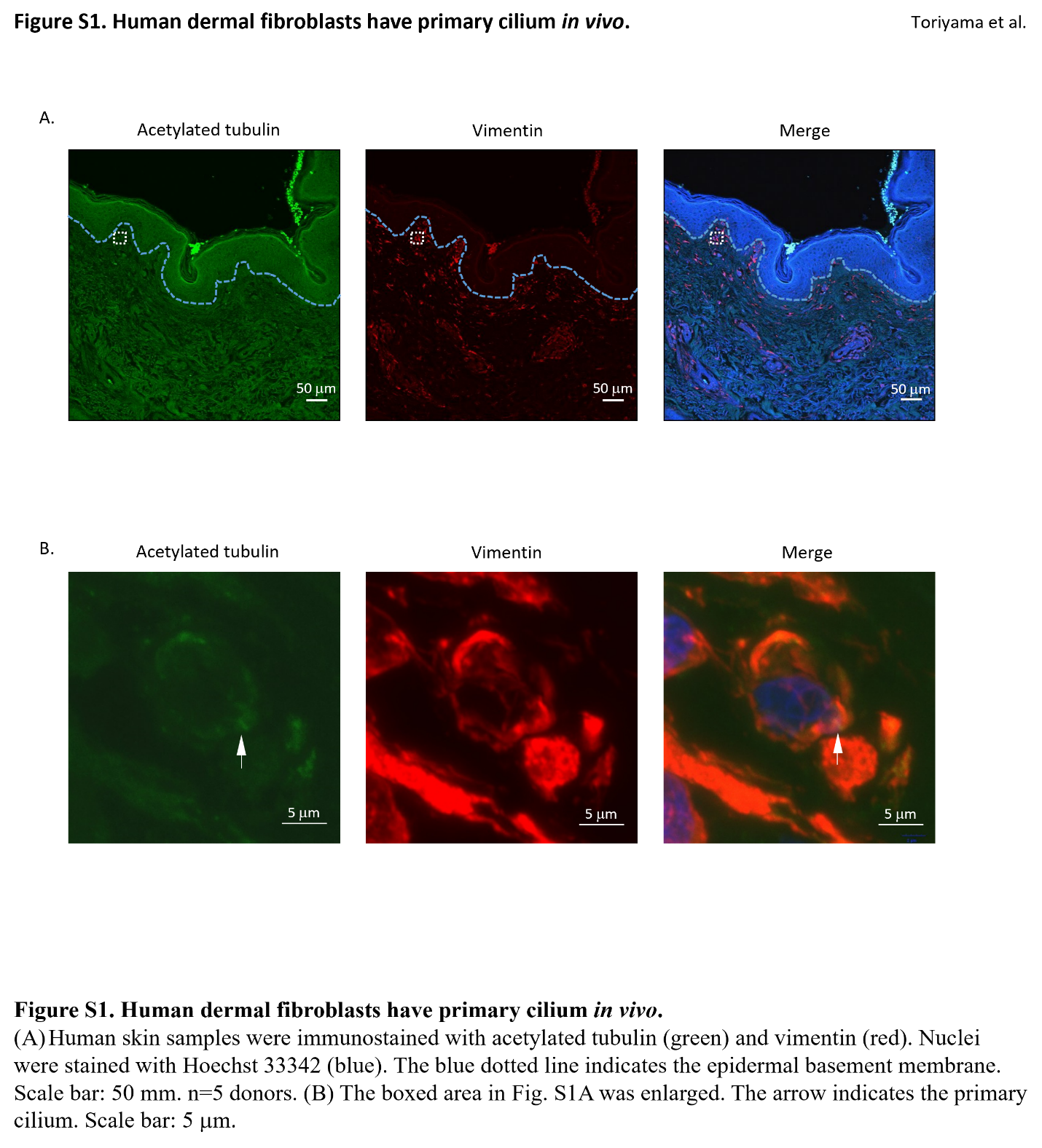


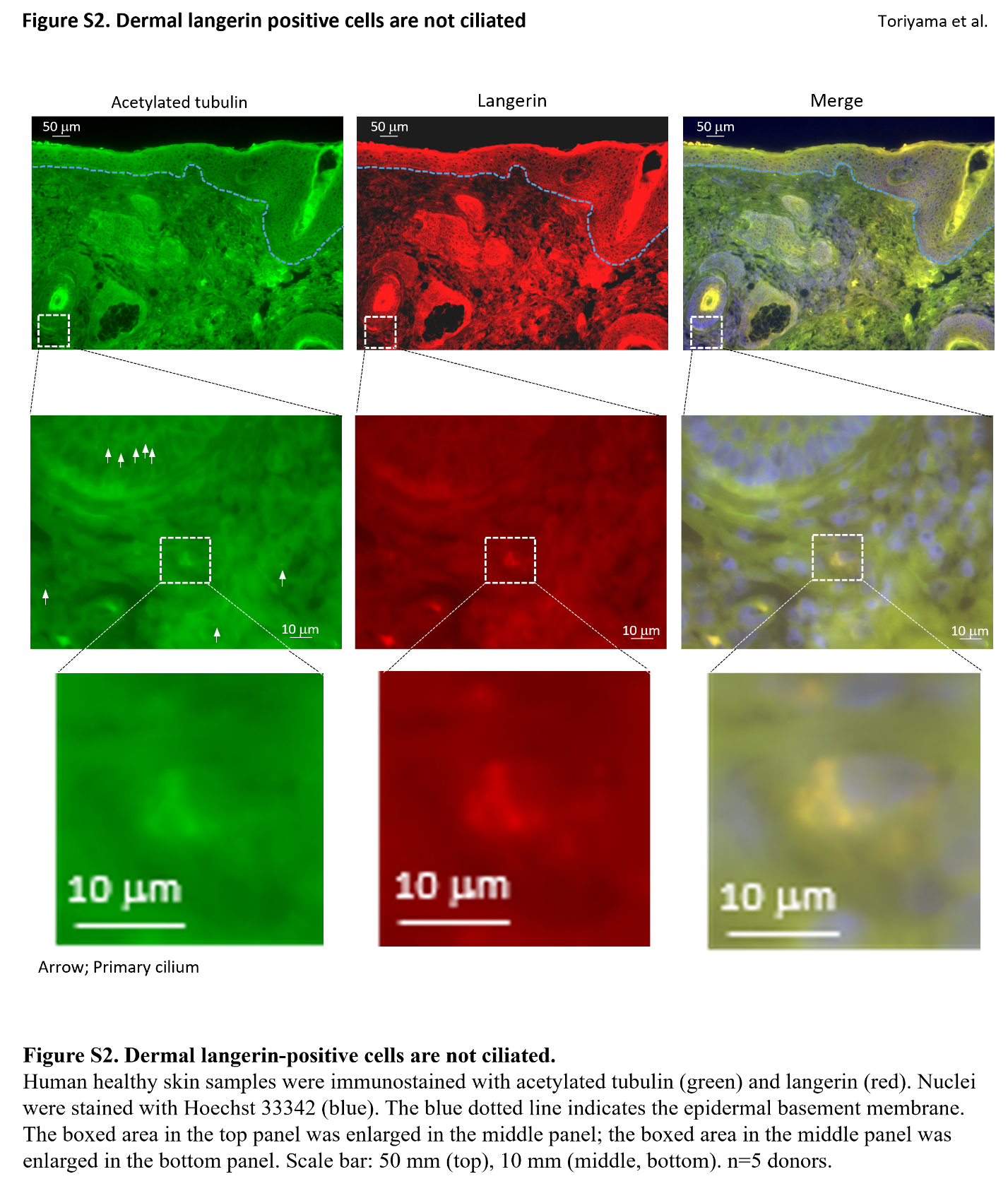


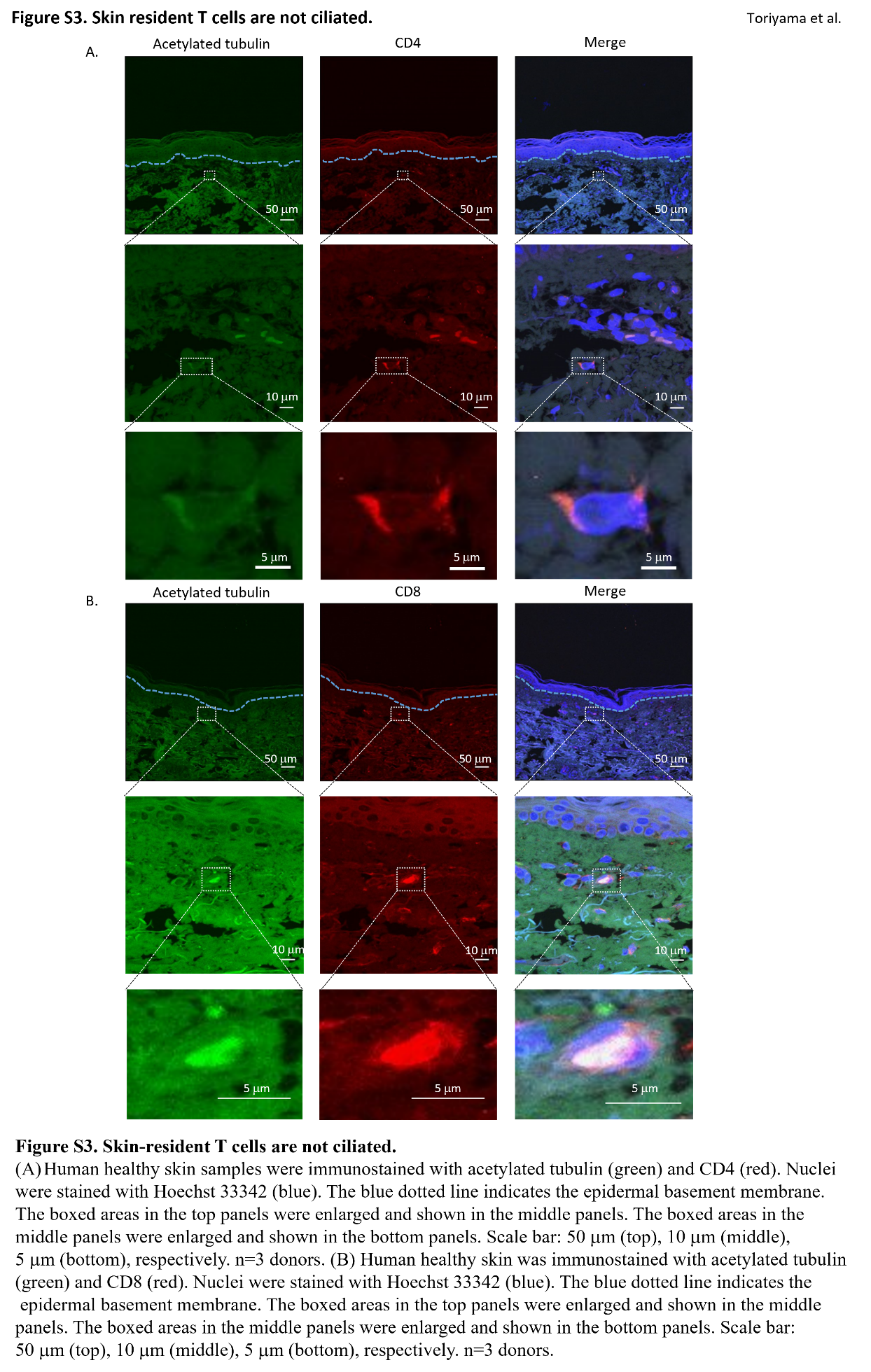


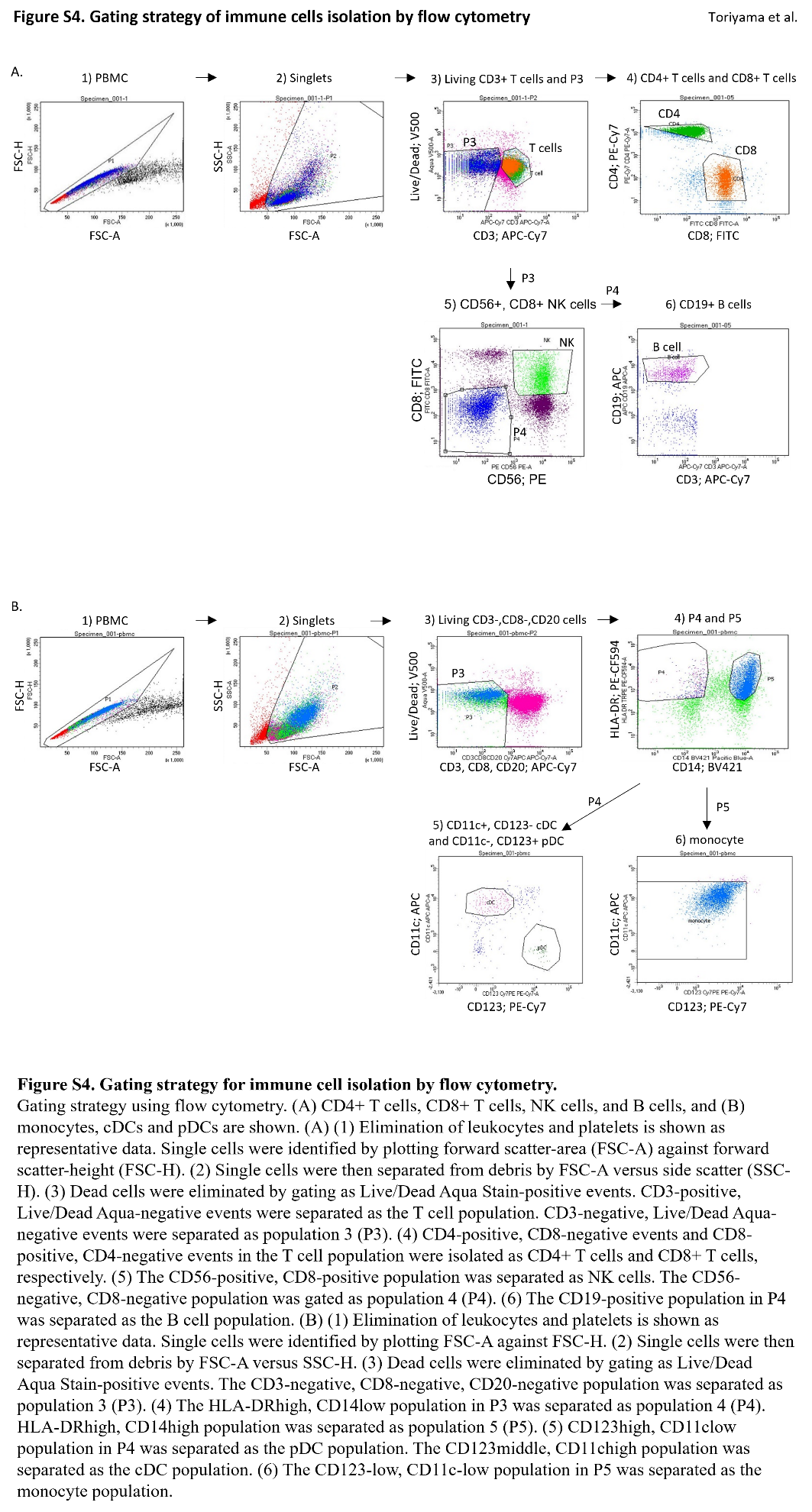


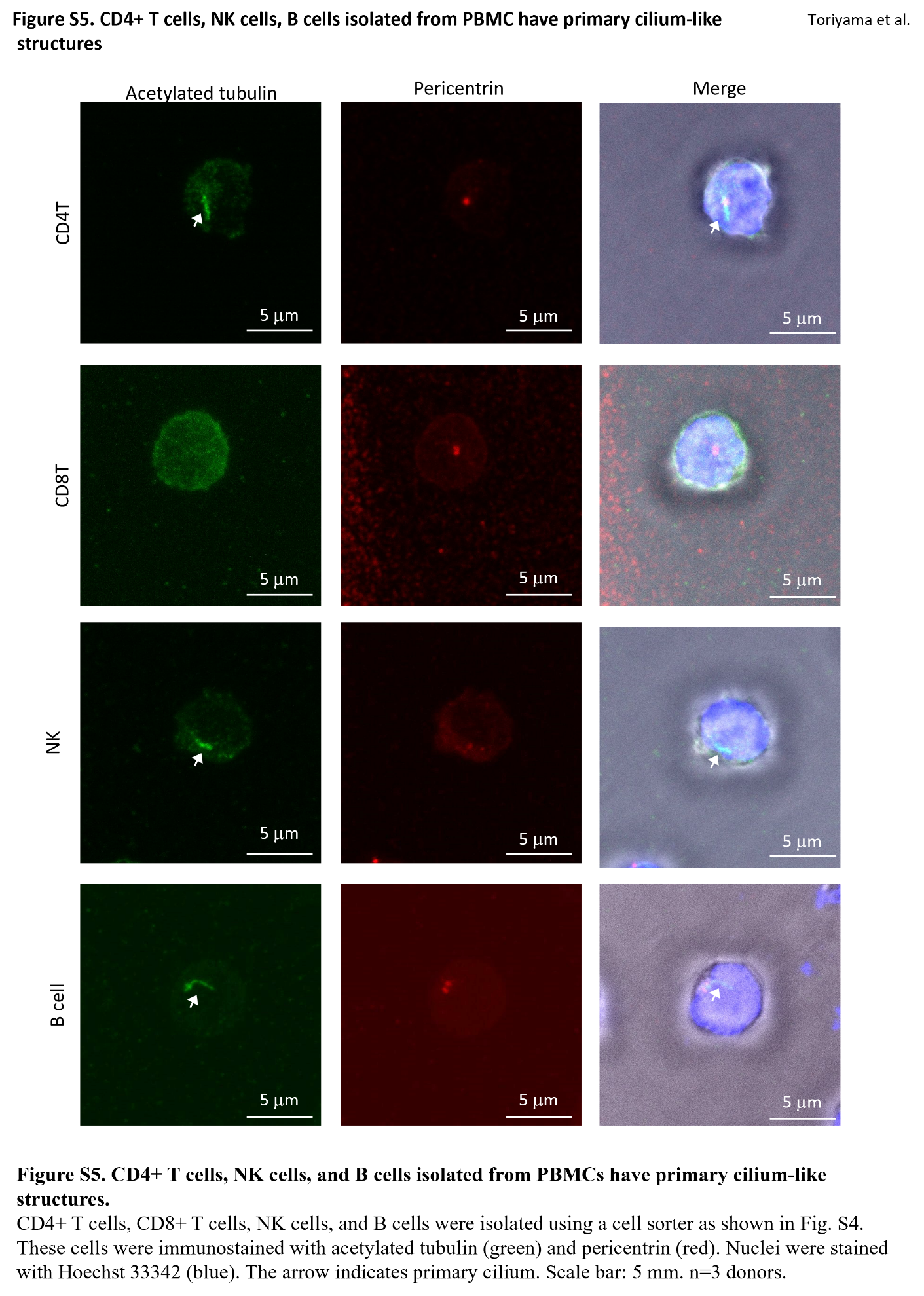


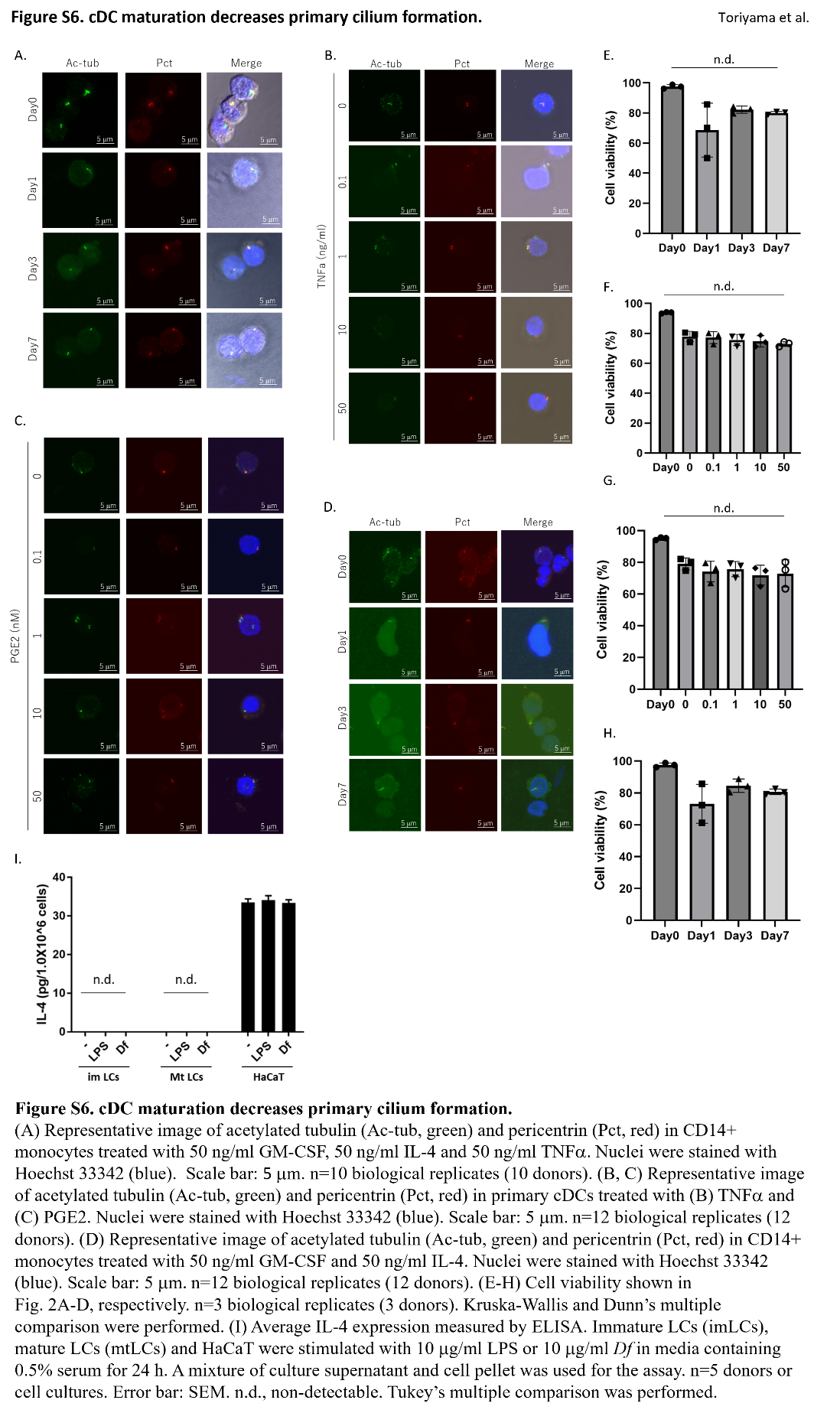


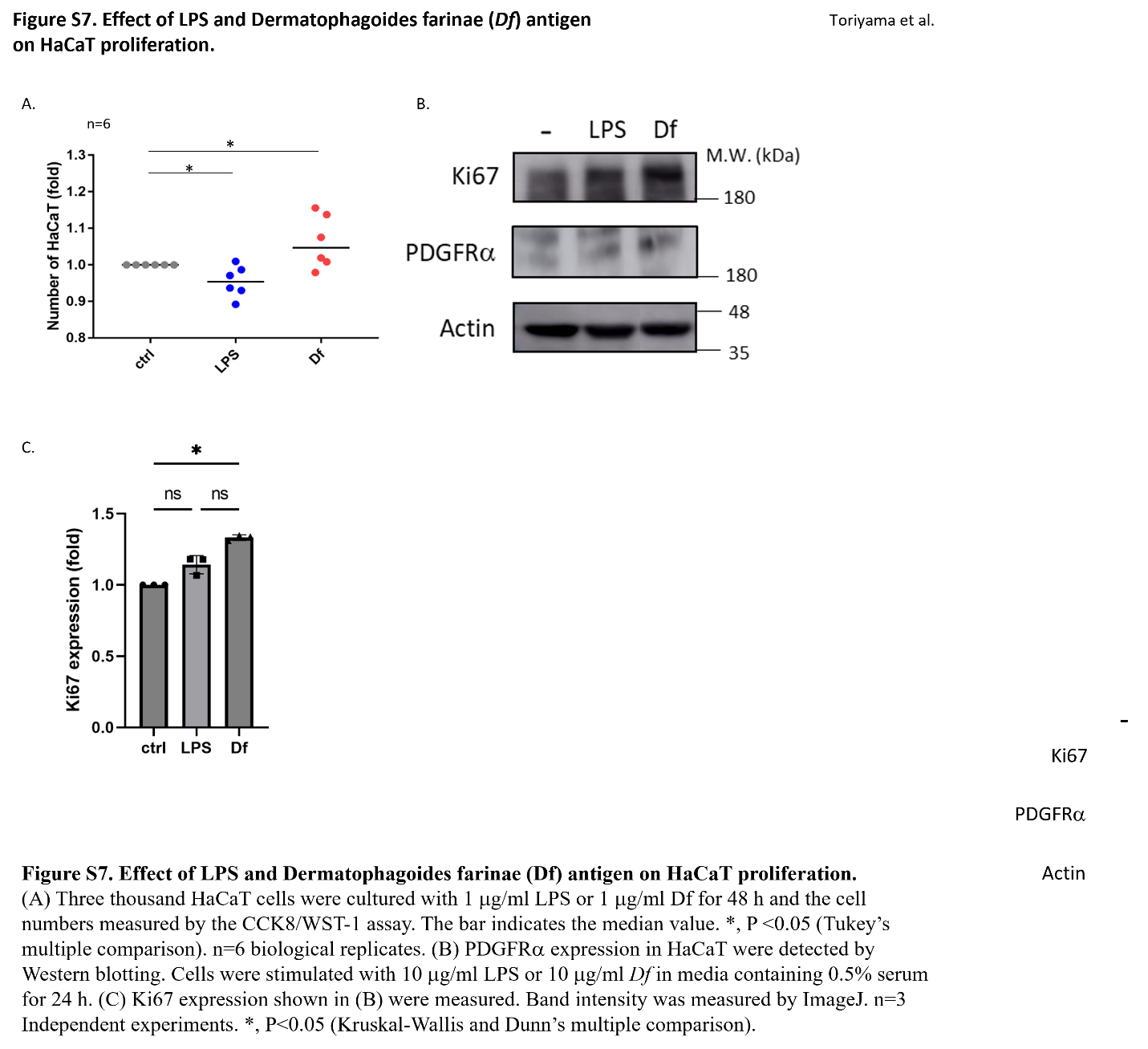


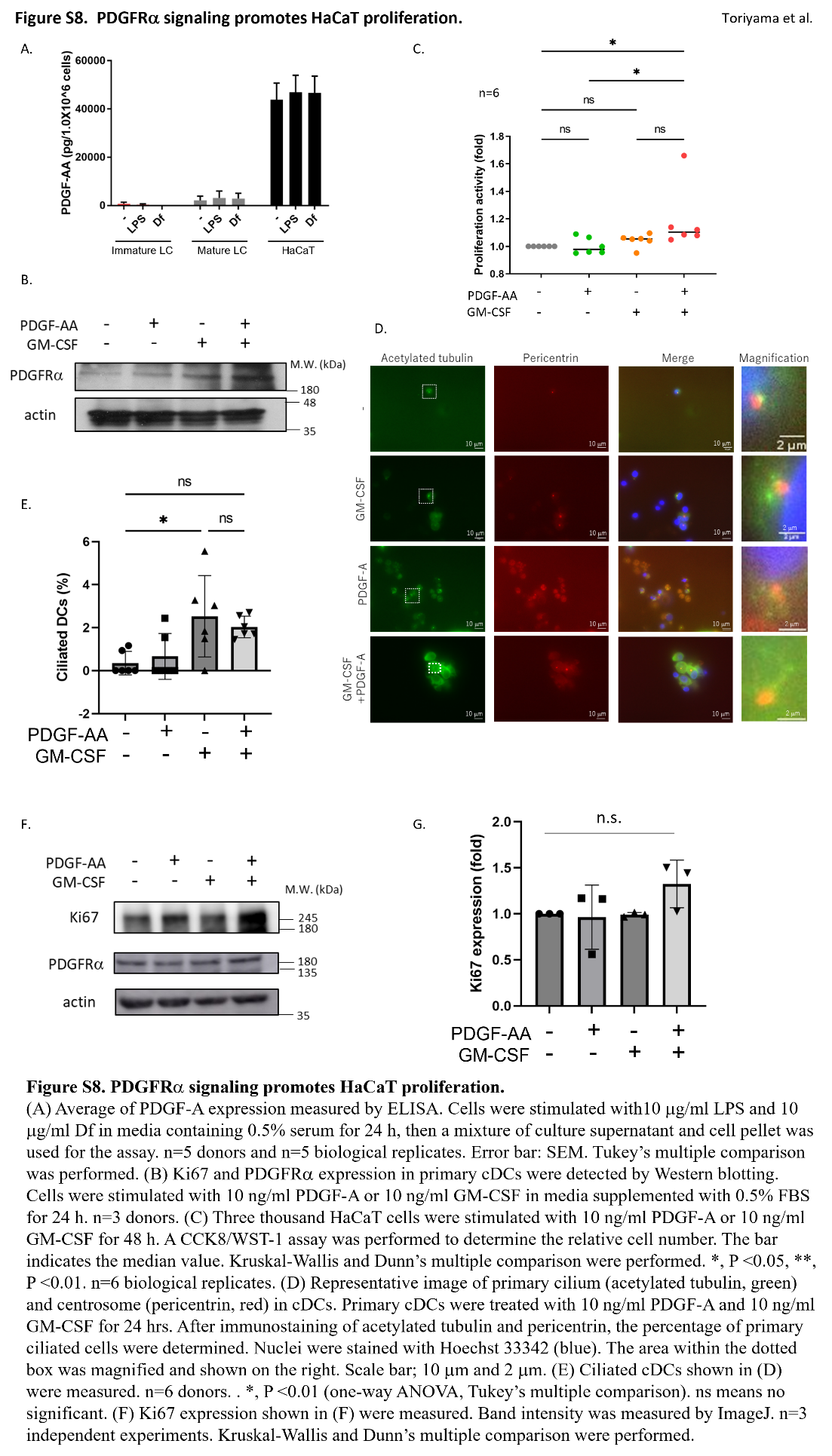


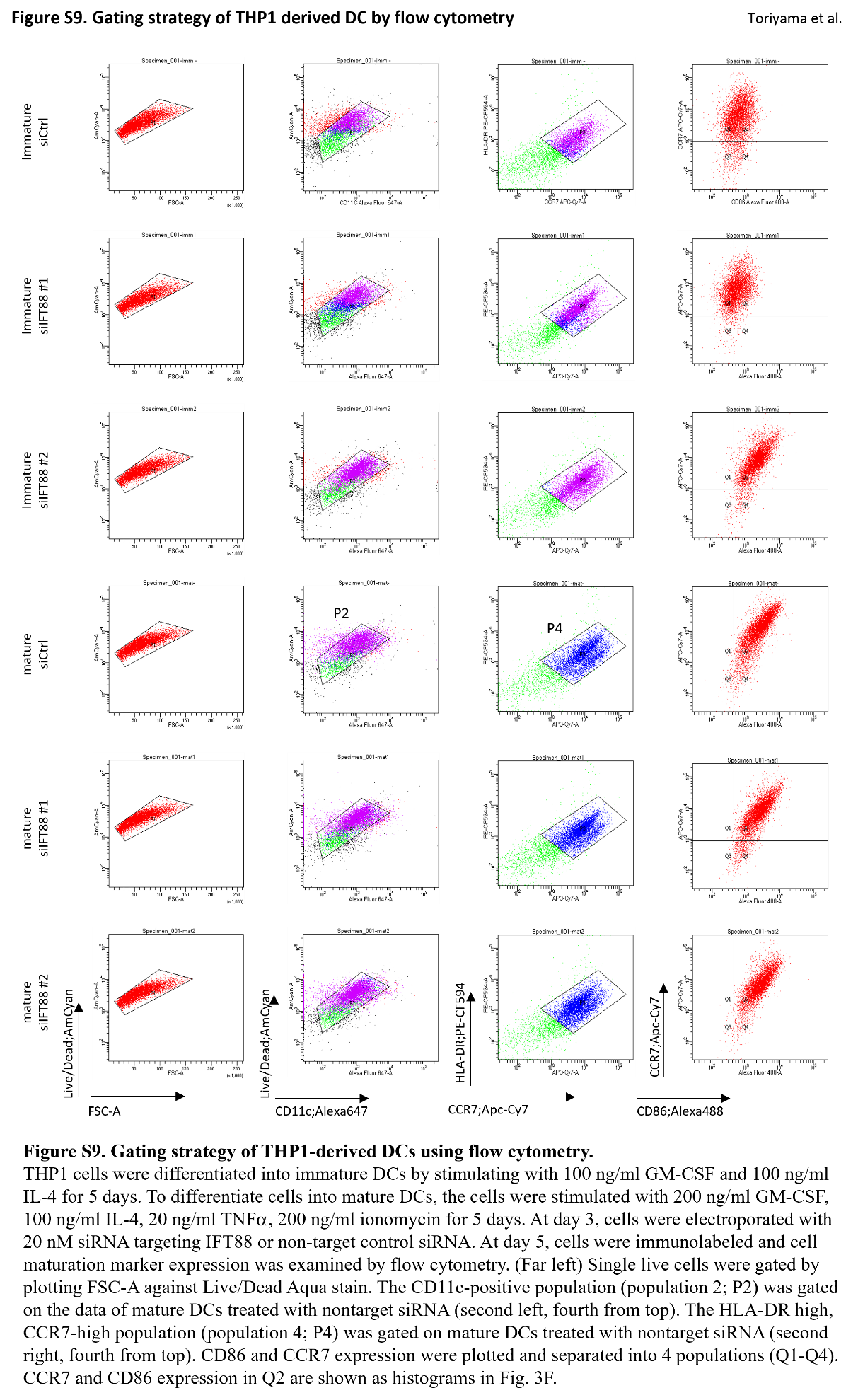


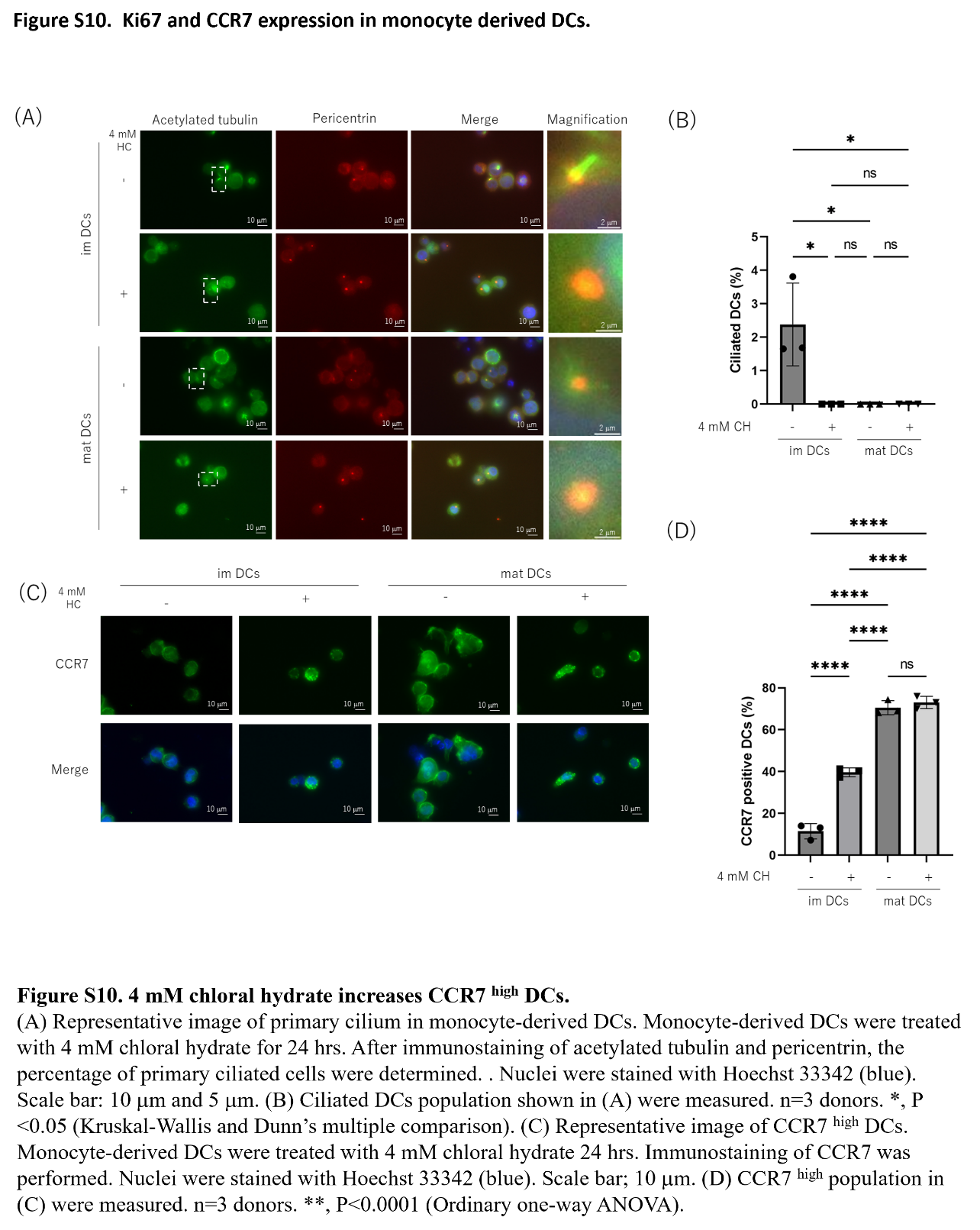


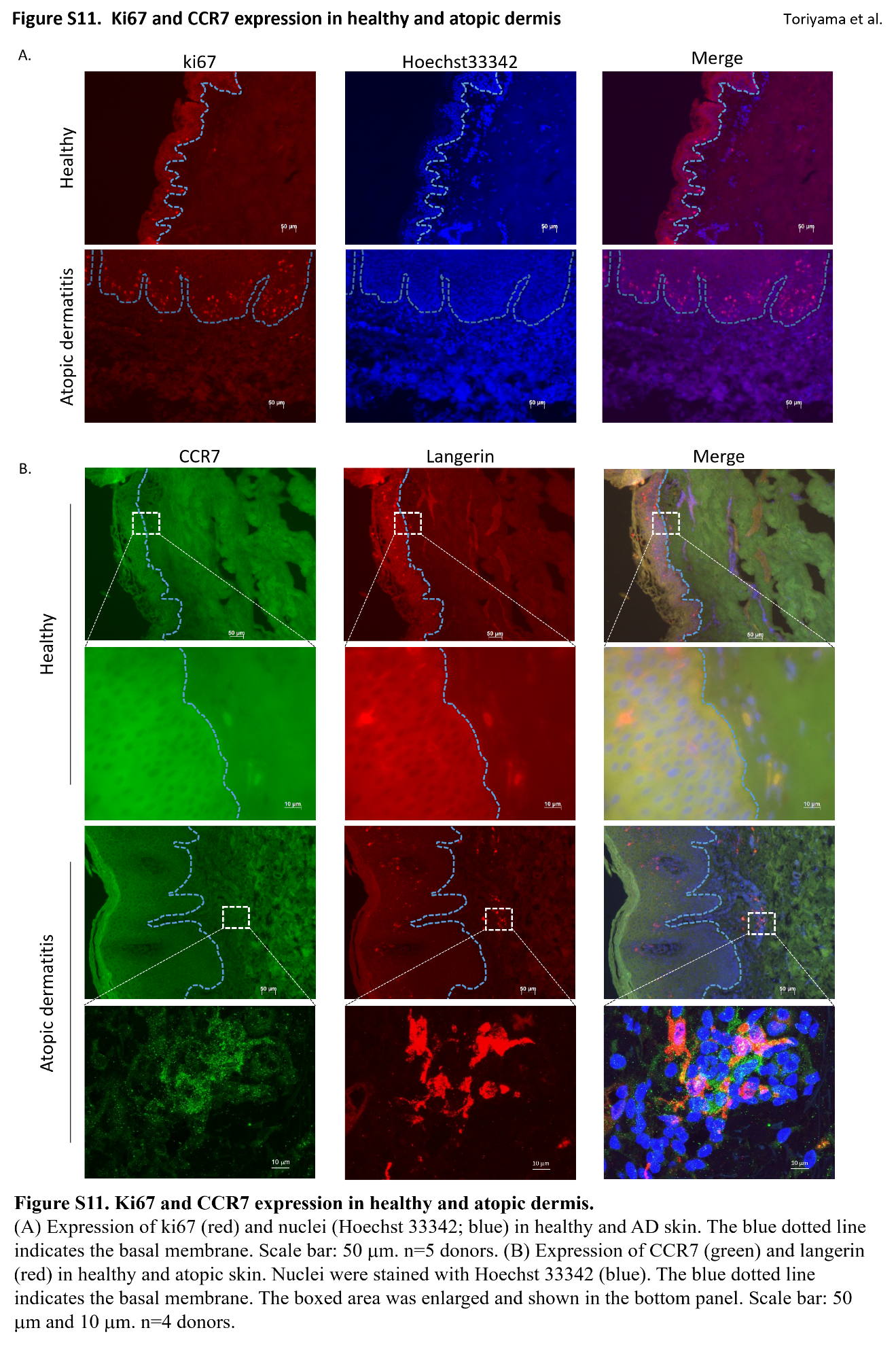


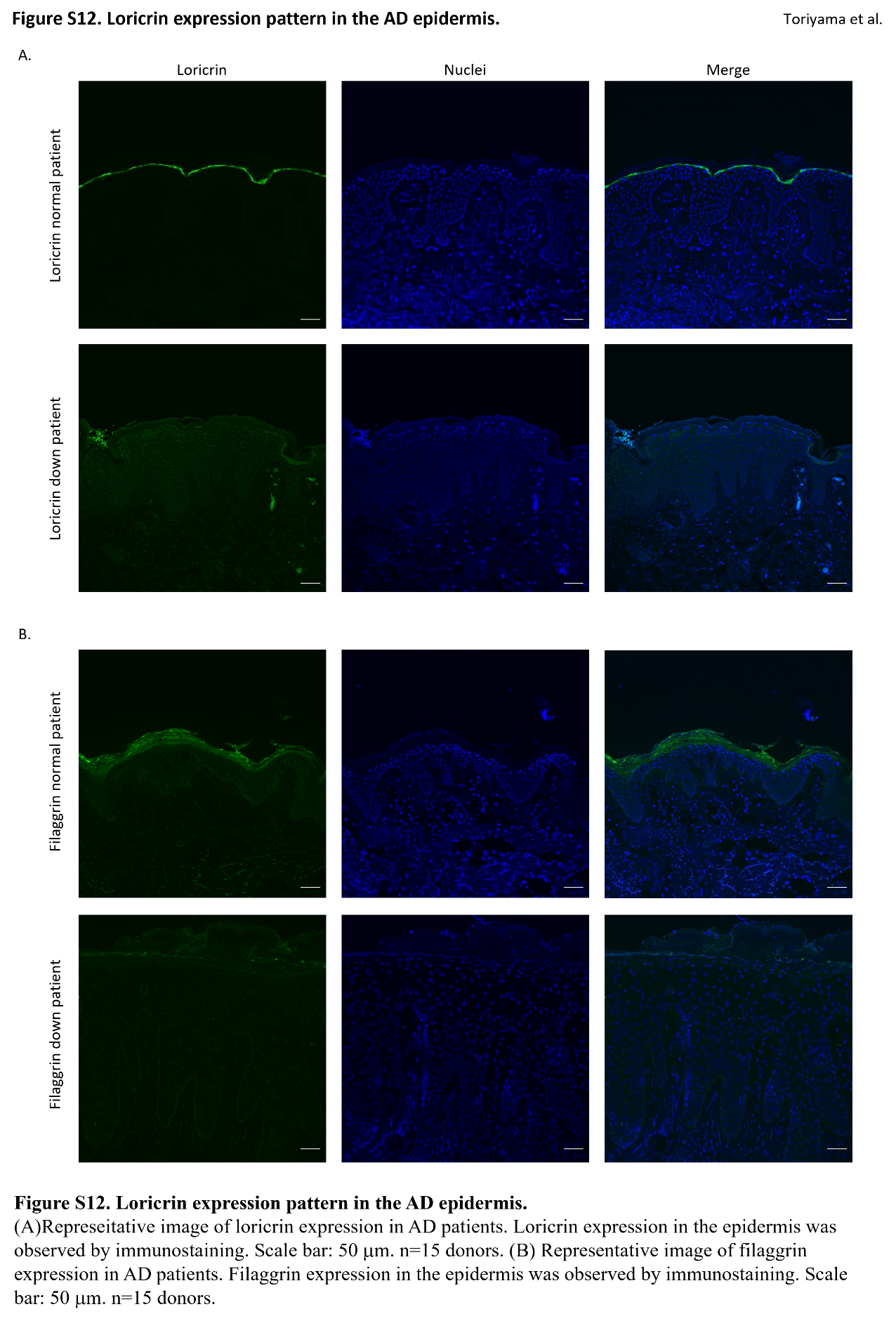


The authors apologize for this error and state that this does not change the scientific conclusions
