# Supplementary material for: Corrigendum: Dendritic cell proliferation by primary cilium in atopic dermatitis
Source: Front Mol Biosci. 2023 Aug 30;10:1215185. doi: 10.3389/fmolb.2023.1215185 (PMC10499438; doi:10.3389/fmolb.2023.1215185)
Supplement: Supplementary file 1 [file DataSheet1.docx]

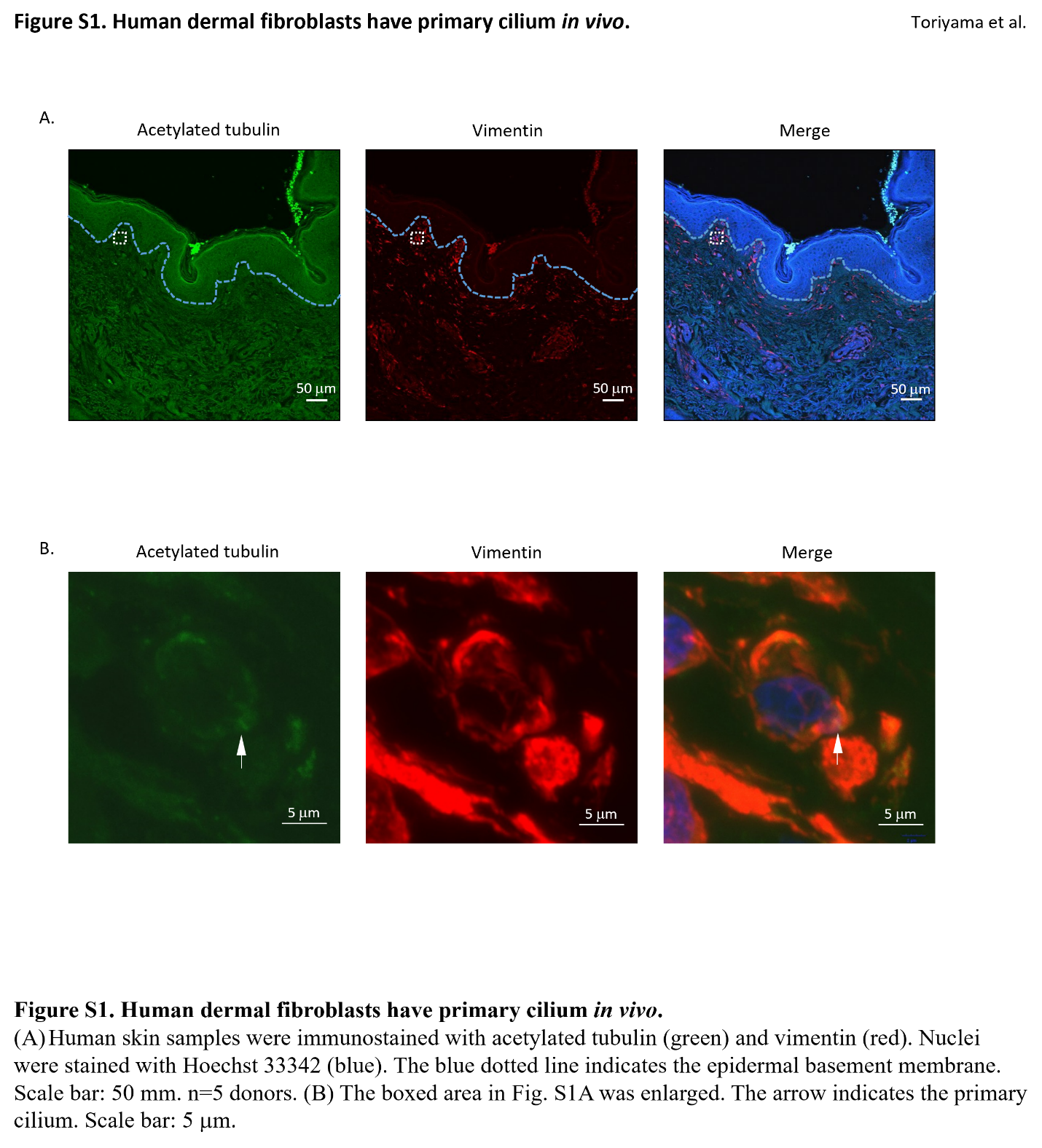


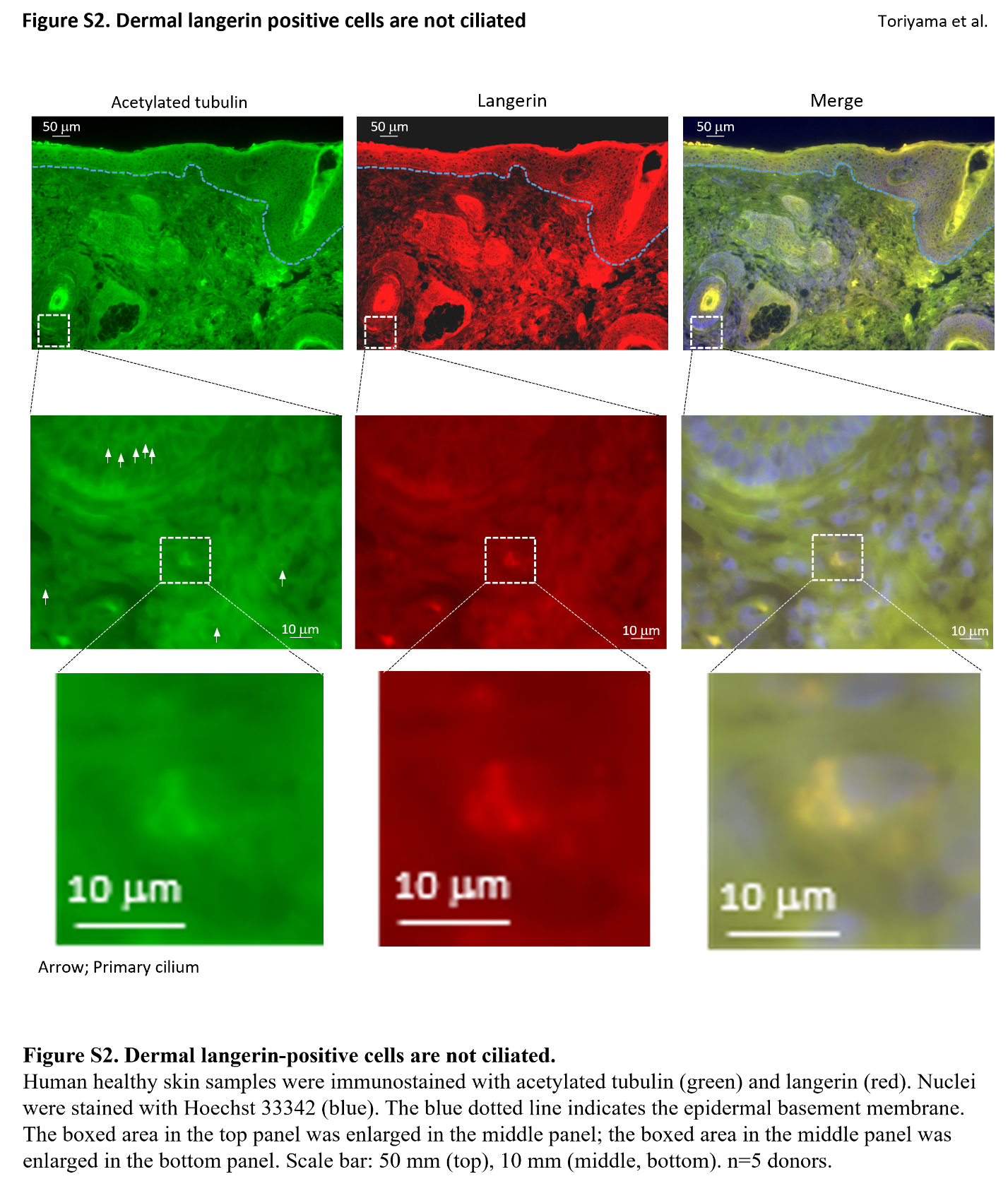


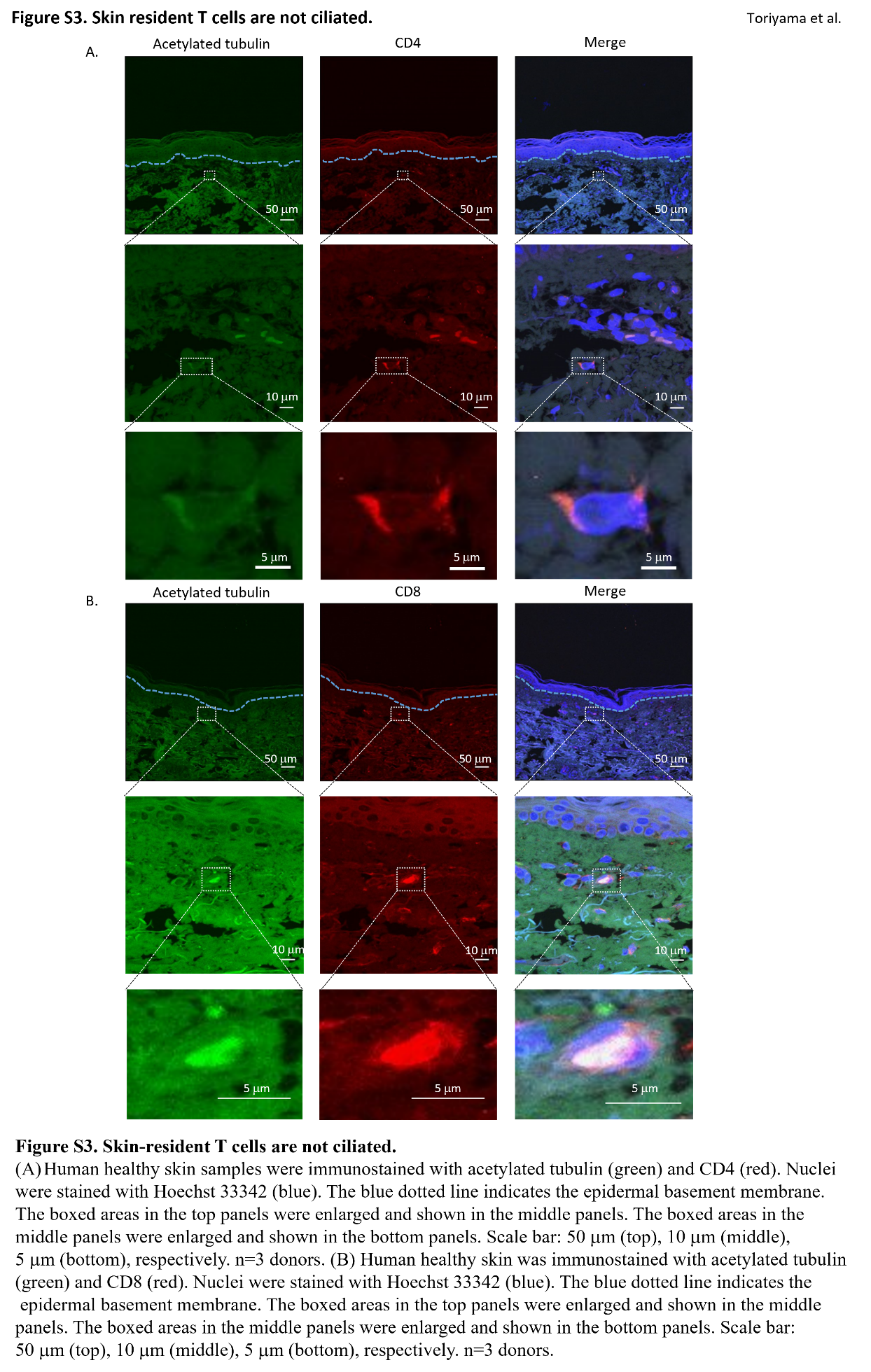


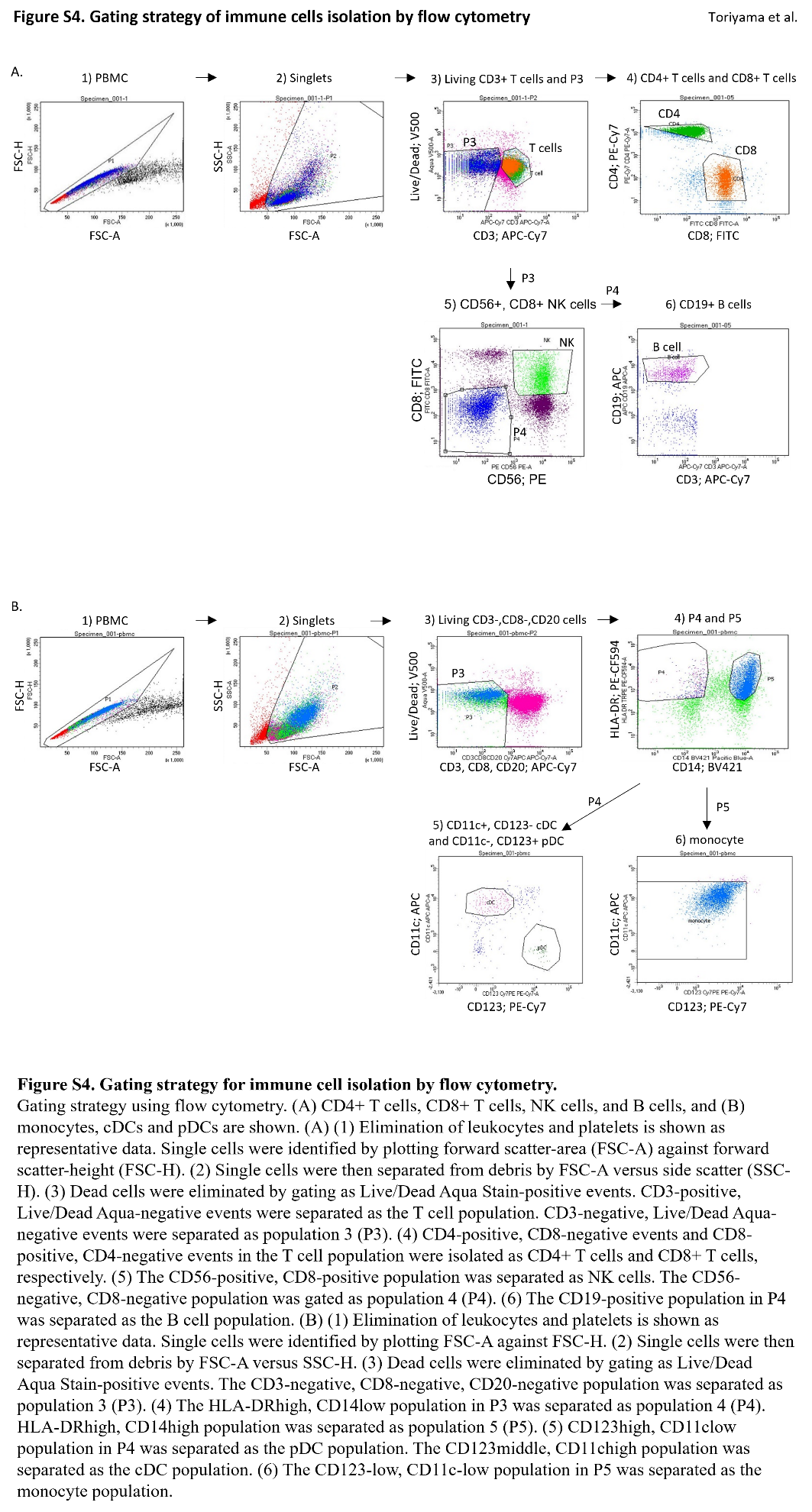


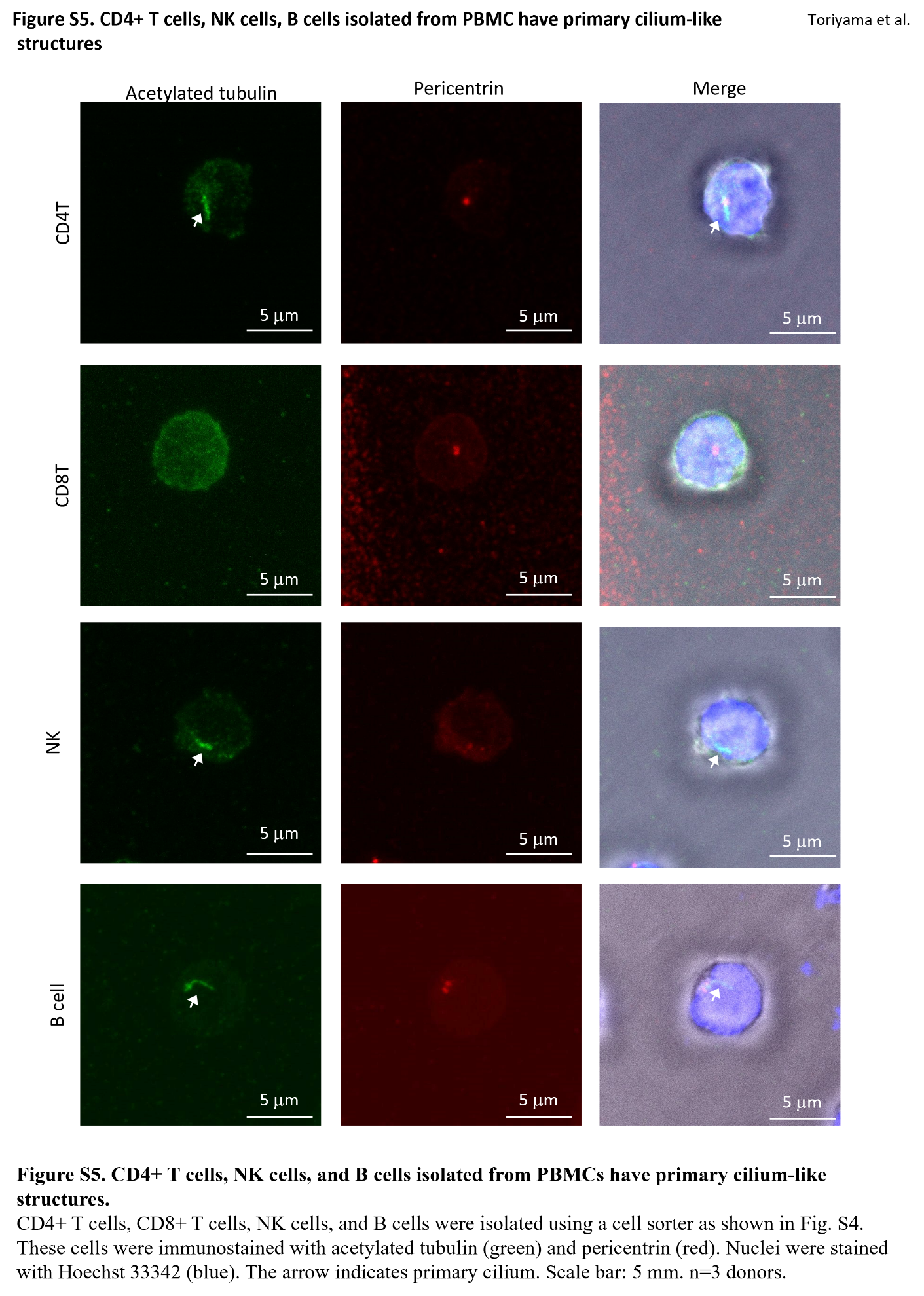


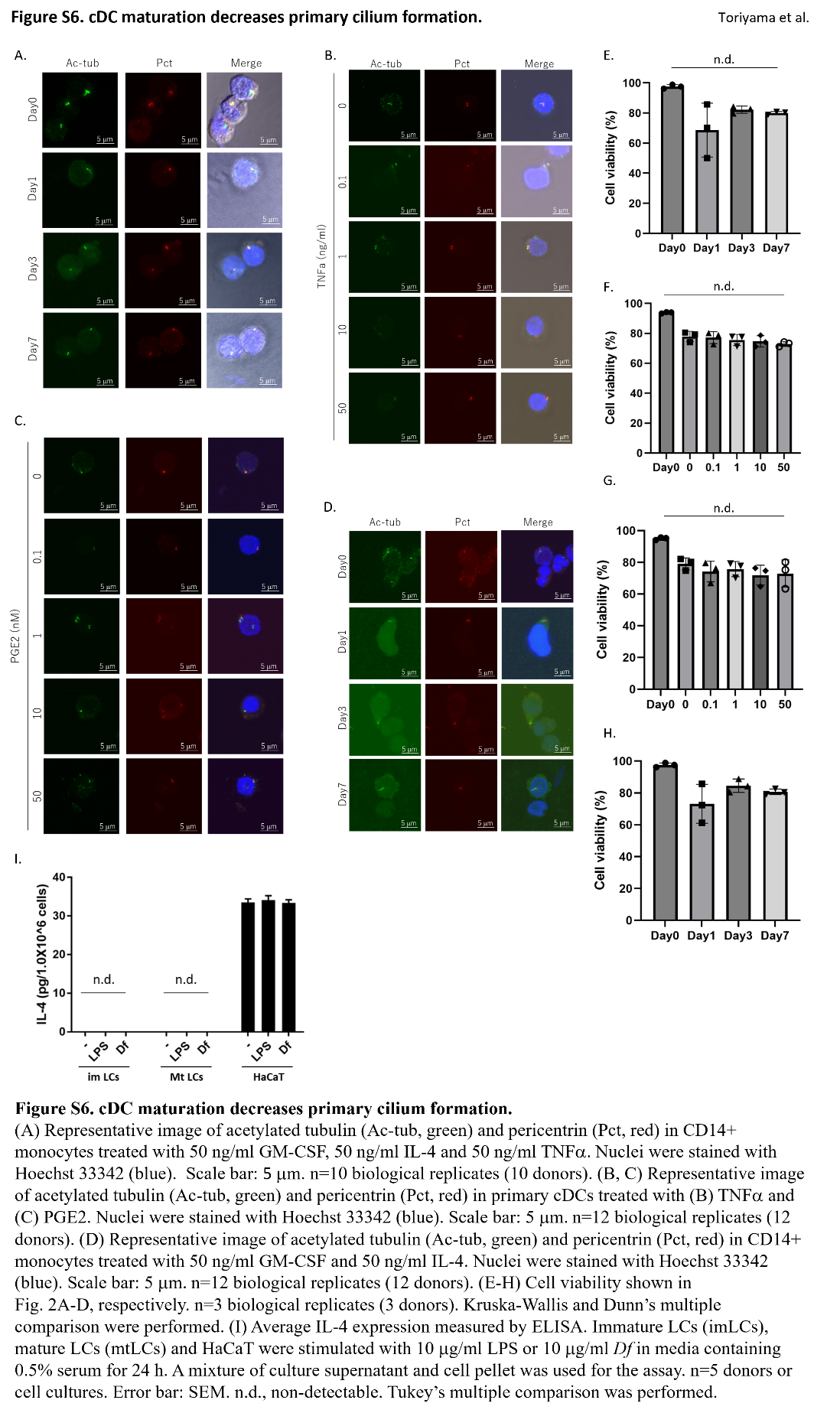


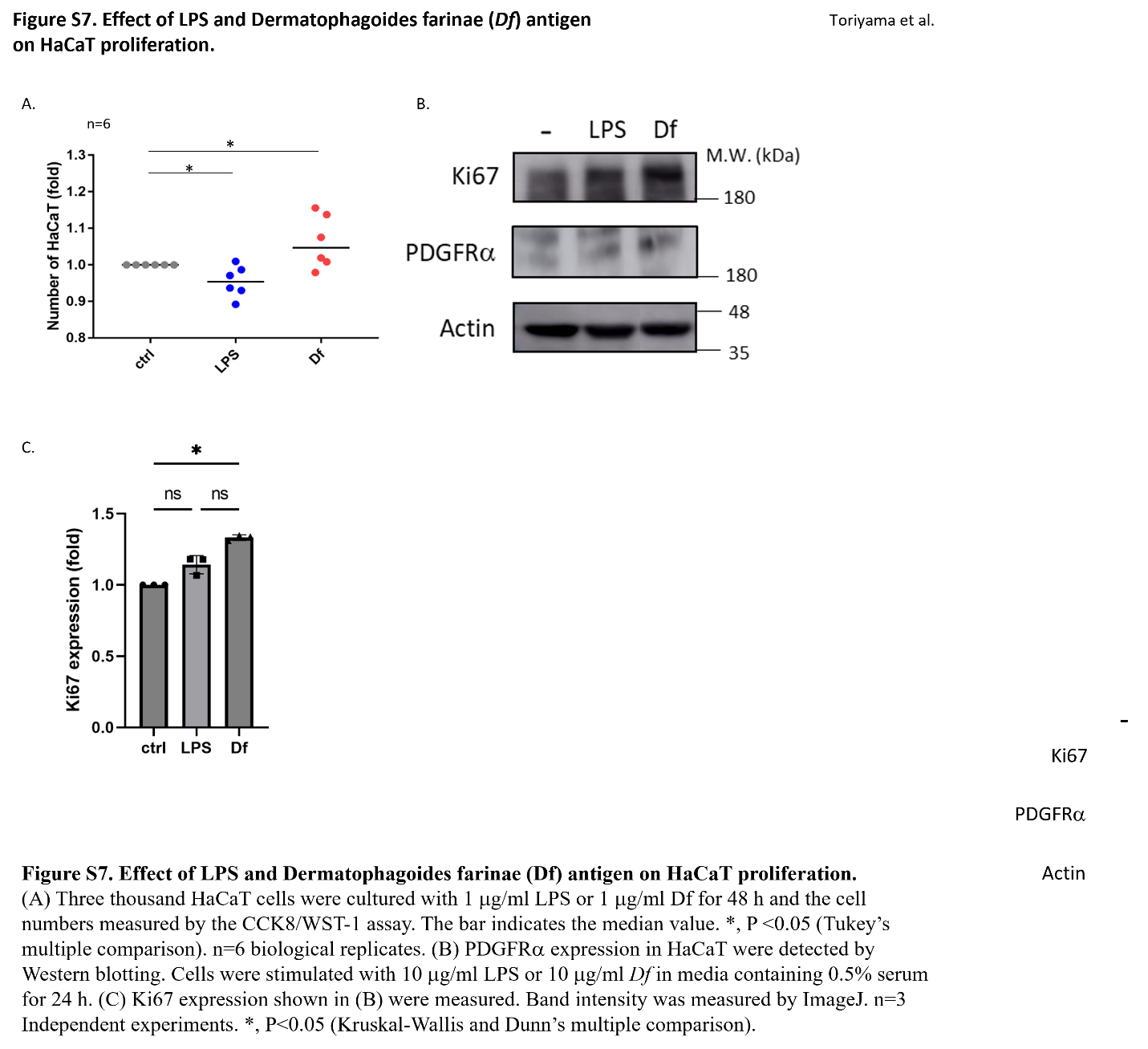


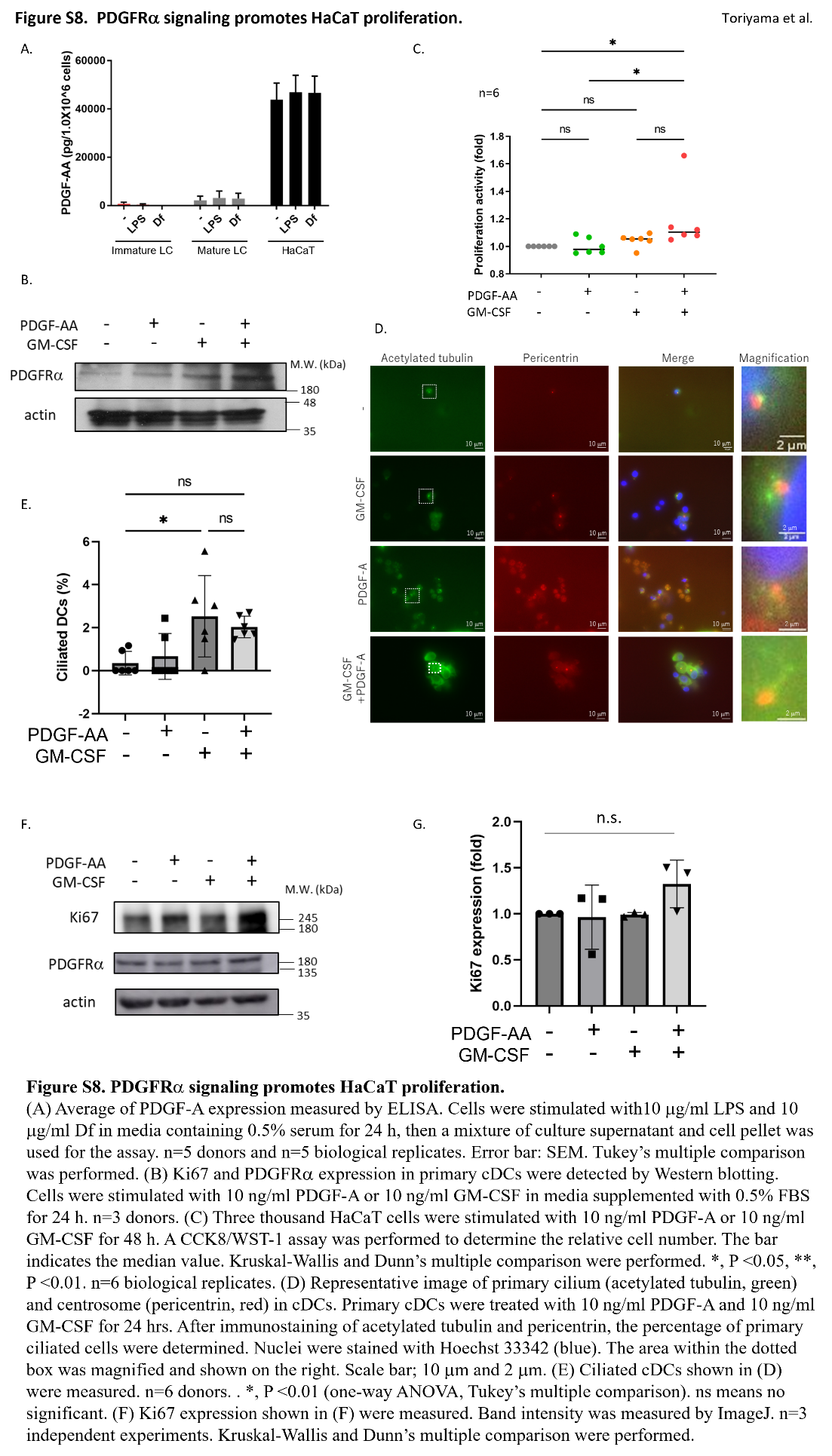


Figure S8. PDGFRα signaling promotes HaCaT proliferation.

(A) Average of PDGF-A expression measured by ELISA. Cells were stimulated with 10 μg/ml LPS and 10 μg/ml *Df* in media containing 0.5% serum for 24 h, then a mixture of culture supernatant and cell pellet was used for the assay. n=5 donors and n=5 biological replicates. Error bar: SEM. Tukey’s multiple comparison was performed. (B) Ki67 and PDGFRα expression in primary cDCs were detected by Western blotting. Cells were stimulated with 10 ng/ml PDGF-A or 10 ng/ml GM-CSF in media supplemented with 0.5% FBS for 24 h. n=3 donors. (C) Three thousand HaCaT cells were stimulated with 10 ng/ml PDGF-A or 10 ng/ml GM-CSF for 48 h. A CCK8/WST-1 assay was performed to determine the relative cell number. The bar indicates the median value. Kruskal-Wallis and Dunn’s multiple comparison were performed. *, P <0.05, **, P <0.01. n=6 biological replicates. (D) Representative image of primary cilium (acetylated tubulin, green) and centrosome (Pericentrin, red) in cDCs. Primary cDCs were treated with 10 ng/ml PDGF-A and 10 ng/ml GM-CSF for 24 hrs. After immunostaining of acetylated tubulin and Pericentrin, the percentage of primary ciliated cells was determined. Nuclei were stained with hoechst 33342 (blue). The area within the dotted box was magnified and shown on the right. Scale bar; 10 μm and 2 μm. (E) Ciliated cDCs shown in (D) were measured. n=6 donors. * P <0.01 (one-way ANOVA, Tukey’s multiple comparison). ns means no significant. (F) Ki67 and PDGFRα expression in HaCaT was detected by Western blotting. Cells were stimulated with 10 ng/ml PDGF-A or 10 ng/ml GM-CSF in media supplemented with 0.5% FBS for 24 h. n=3 donors. (G) Ki67 expression shown in (F) were measured. Band intensity was measured by image J. n=3 independent experiments. Kruskal-Wallis and Dunn’s multiple comparison were performed.


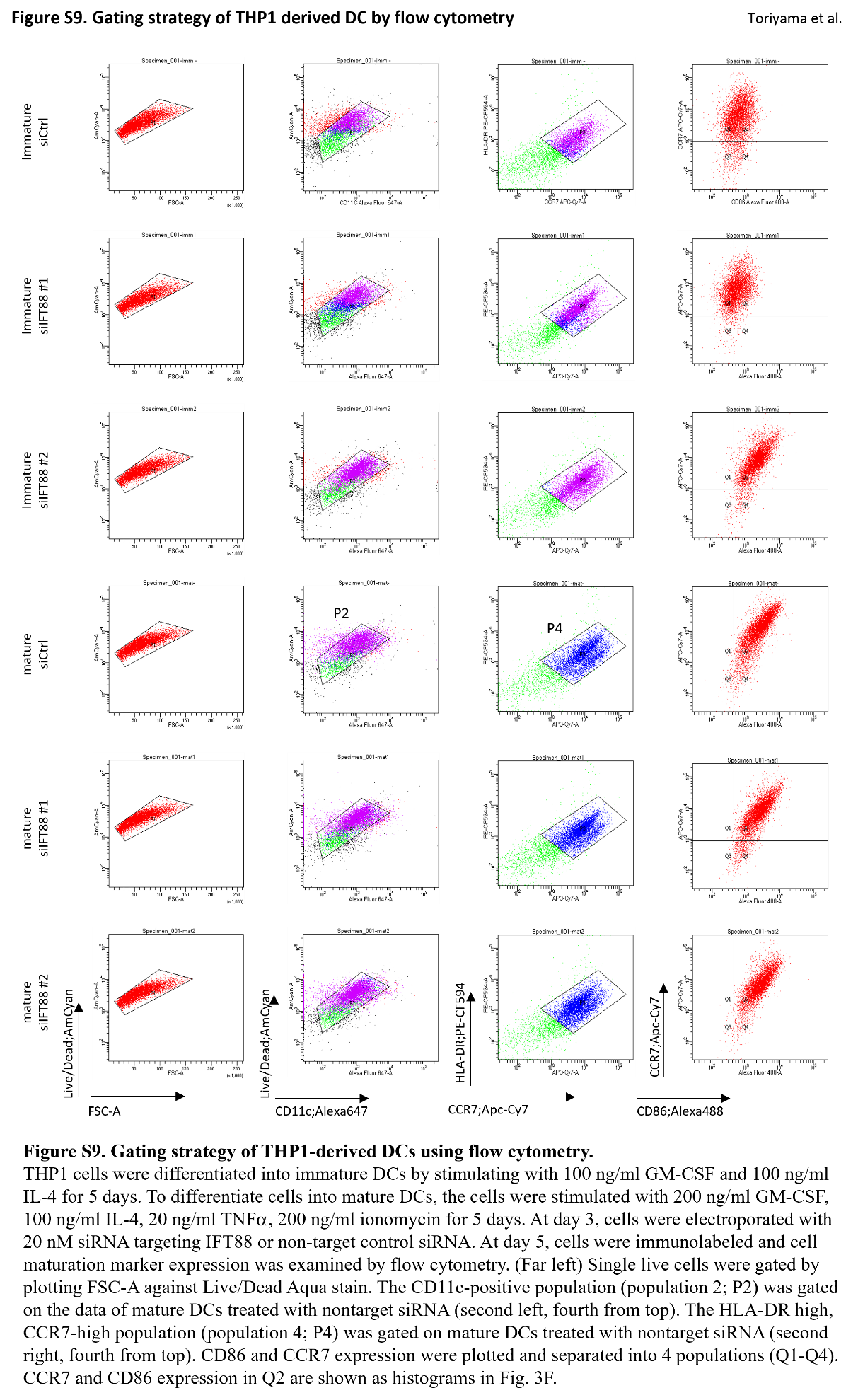


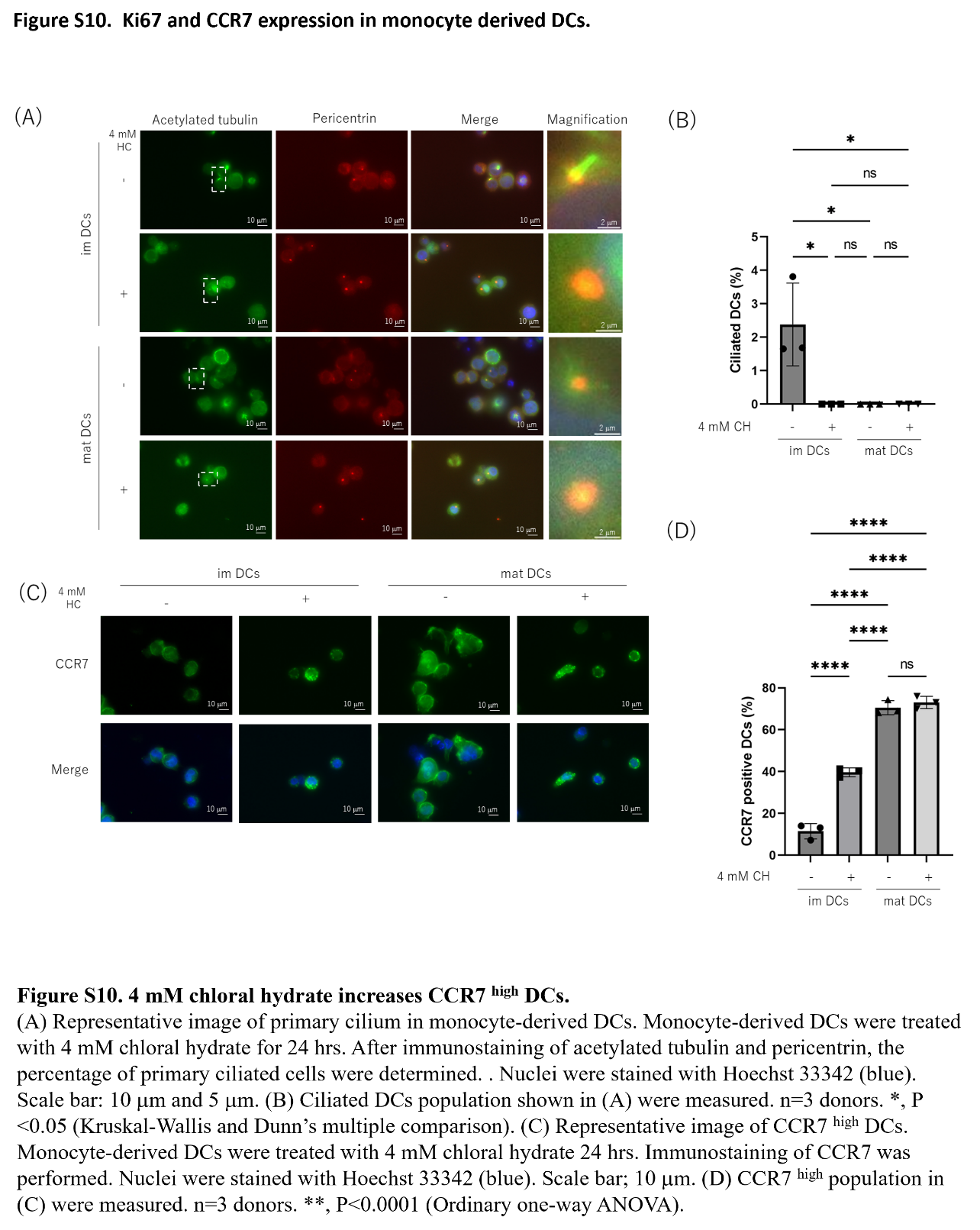


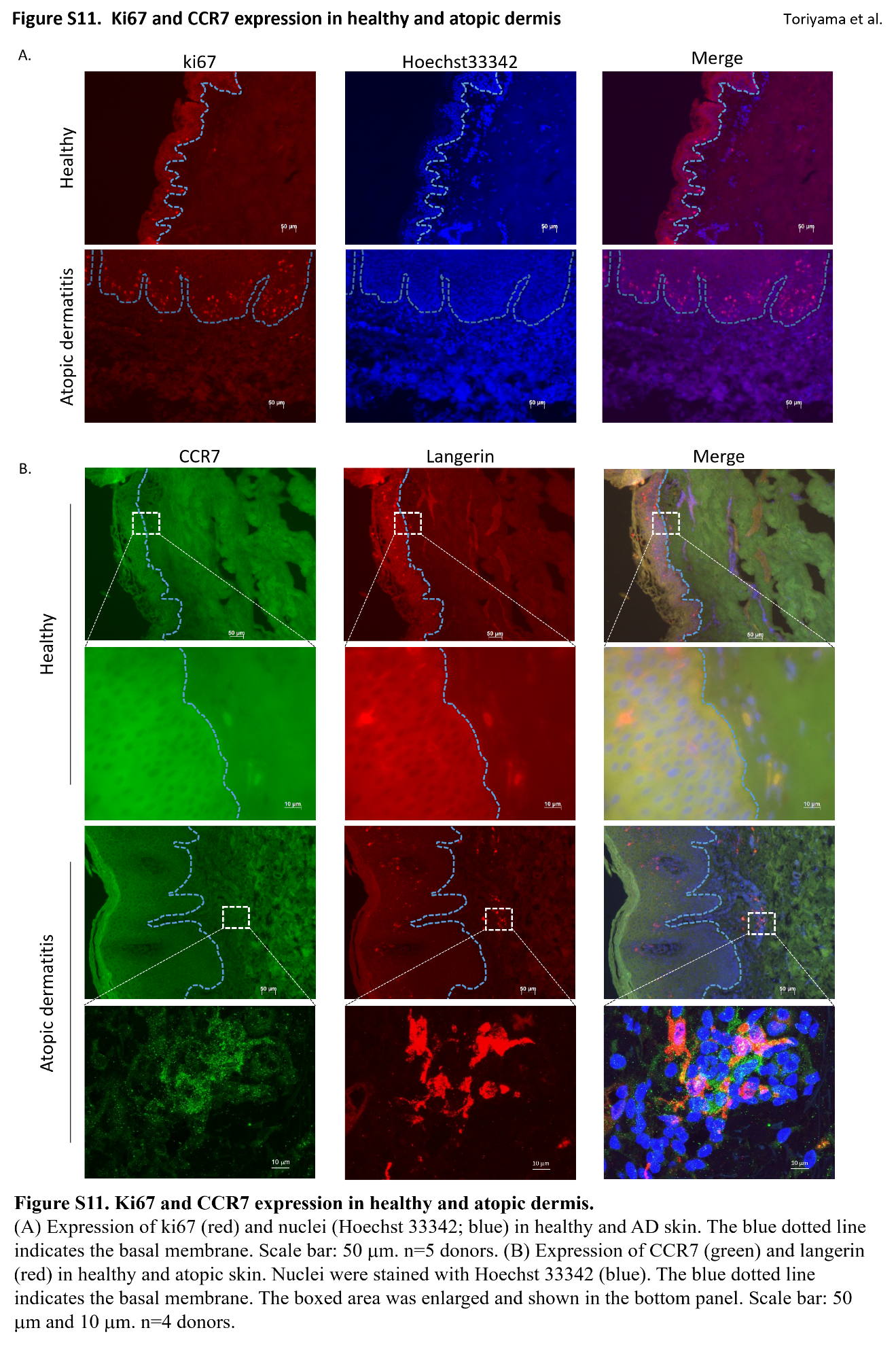


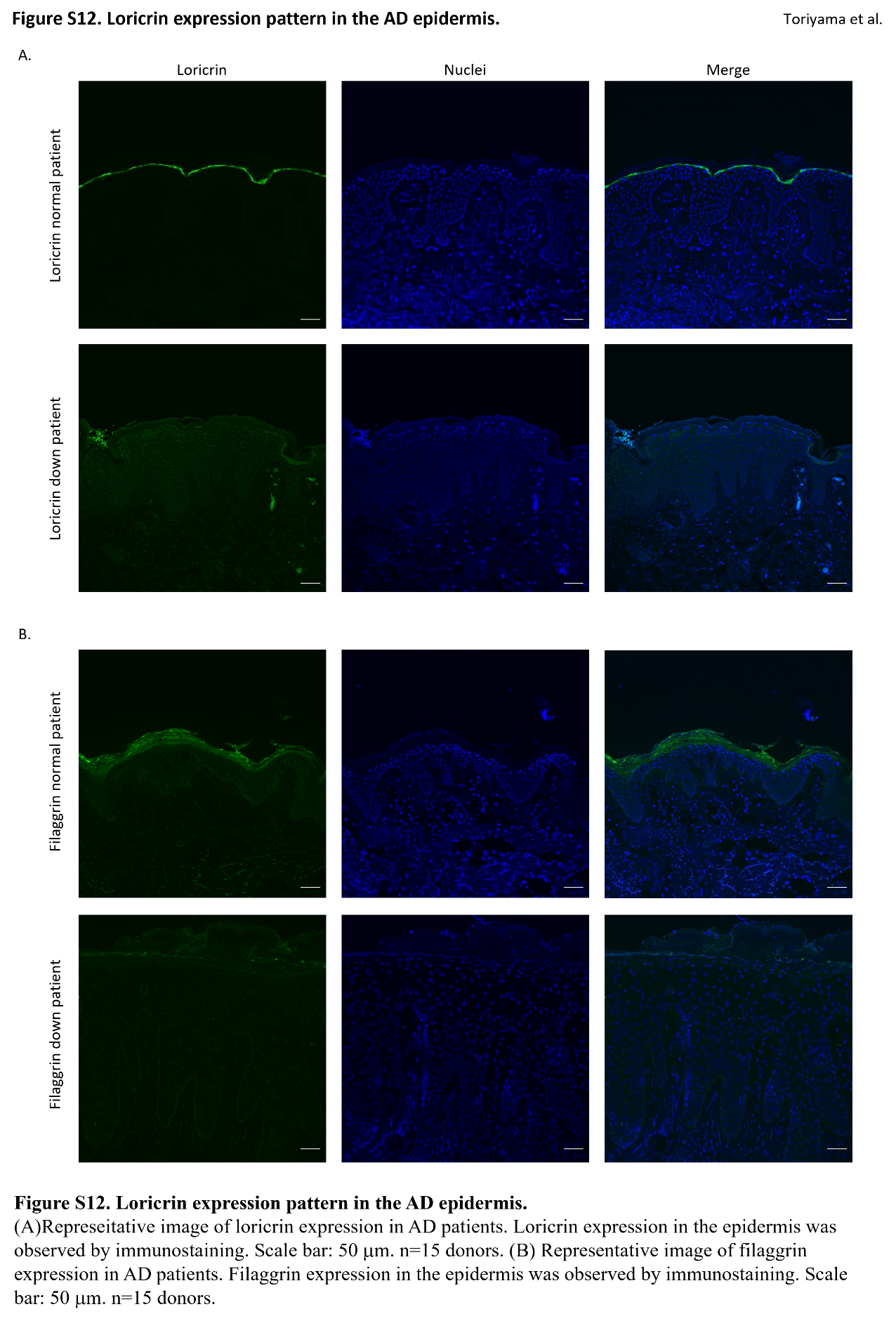


The authors apologize for this error and state that this does not change the scientific conclusions
